# Vericiguat attenuates cyclosporine A-induced nephropathy by targeting the NF-κB/TGF-β1 axis: an integrated network pharmacology, Mendelian randomization, and experimental study

**DOI:** 10.3389/fimmu.2025.1756582

**Published:** 2026-01-27

**Authors:** XuanKe Liu, MengJie Tang, JiaYing Lu, YiJing Kong, WenXuan Shen, ChunJiang Zhang, XiaoPing Yang

**Affiliations:** 1 Department of Nephrology, The First Affiliated Hospital of Shihezi University, Shihezi, China; 2 Department of Nephrology, National Health Commission (NHC) Key Laboratory of Prevention and Treatment of Central Asia High Incidence Diseases (Co-construction), Shihezi, China

**Keywords:** cyclosporine A nephropathy, Mendelian randomization, network pharmacology, NF-κB/TGF-β1 axis, renoprotection, vericiguat

## Abstract

**Background:**

Cyclosporine A (CsA)-induced nephrotoxicity is a significant cause of chronic kidney disease (CKD), primarily driven by aberrant activation of the NF-κB and TGF-β1 signaling axes. Vericiguat, a soluble guanylate cyclase (sGC) stimulator, has established cardioprotective effects, but its potential renoprotective role and underlying mechanisms in CsA-induced CKD remain unexplored.

**Methods:**

An integrative approach was employed. Network pharmacology identified common targets between vericiguat and CKD. Mendelian randomization (MR) analysis assessed the causal relationship between drug target genes and CKD risk. The therapeutic effects and mechanisms of vericiguat were subsequently validated *in vivo* using a CsA-induced mouse model and *in vitro* in human renal tubular epithelial (HK-2) cells.

**Results:**

Integrated computational analyses pinpointed the NF-κB/TGF-β1 axis as a core target of vericiguat. In mice, vericiguat treatment dose-dependently ameliorated CsA-induced renal dysfunction, proteinuria, renal inflammation, oxidative stress, and fibrosis. In HK-2 cells, vericiguat suppressed CsA-triggered inflammatory cytokine secretion, fibrotic marker expression, and reactive oxygen species production. Mechanistically, vericiguat inhibited the phosphorylation of the IKKβ/IκBα/NF-κB p65 pathway and the activation of TGF-β1/Smad signaling, thereby disrupting the inflammation-fibrosis vicious cycle. Genetic manipulation confirmed p65 as a crucial nodal point in this regulatory network.

**Conclusion:**

This study demonstrates that vericiguat exerts renoprotective, anti-inflammatory, and anti-fibrotic effects in CsA-induced CKD by modulating the NF-κB/TGF-β1 axis. These findings provide a novel scientific rationale for drug repurposing of vericiguat and highlight its potential therapeutic value for CKD.

## 1 Introduction

Chronic kidney disease (CKD) represents a pressing global health burden, affecting over 800 million individuals and contributing substantially to worldwide morbidity and mortality (1). Etiologies are varied, encompassing diabetes, hypertension, and, importantly, drug-induced nephrotoxicity (2–5). Cyclosporine A (CsA), a calcineurin inhibitor fundamental to organ transplantation and autoimmune disease management, epitomizes this iatrogenic concern (6). Despite its clinical utility, long-term CsA administration frequently leads to chronic nephropathy, marked by progressive tubular damage, interstitial inflammation, fibrosis, and eventual irreversible loss of renal function (7). The pathogenesis of CsA-induced CKD is multifactorial, with compelling evidence implicating aberrant activation of the Nuclear Factor Kappa B (NF-κB) and Transforming Growth Factor Beta 1 (TGF-β1) signaling pathways as central drivers of inflammation and fibrosis, respectively. NF-κB orchestrates a potent pro-inflammatory cascade (8, 9), while TGF-β1 acts as a master regulator of fibrosis, stimulating excessive extracellular matrix (ECM) deposition (10, 11). Critically, crosstalk between these pathways creates a self-sustaining vicious cycle of inflammation and maladaptive repair. Nonetheless, targeted therapies specifically interrupting this axis in CsA nephropathy represent a significant unmet medical need.

Vericiguat, a novel oral stimulator of soluble guanylate cyclase (sGC), demonstrated efficacy in reducing heart failure hospitalizations in the VICTORIA Phase III trial involving over 5,000 patients with reduced ejection fraction (12, 13). Its mechanism involves amplifying cyclic guanosine monophosphate (cGMP) signaling, a pathway renowned for its anti-inflammatory and anti-fibrotic properties (14, 15). Intriguingly, beyond its established cardioprotective role, vericiguat may harbor significant renoprotective potential. While preliminary clinical data indicate its safety in heart failure patients with concomitant CKD (16), and a single preclinical study suggested a direct renal benefit in a cardiorenal syndrome model (17), a systematic exploration of its direct effects and mechanisms in classic inflammatory–fibrotic CKD contexts—particularly CsA-nephropathy—is lacking. Given the shared pathobiological pathways underpinning cardiac and renal fibrosis, such as oxidative stress, inflammation, and fibrosis, we postulated that vericiguat may confer benefits in CKD by modulating the NF-κB/TGF-β1 axis—a hypothesis initially informed by integrated network pharmacology and Mendelian randomization screening. We selected vericiguat for this study based on several rationales: 1) its established role in enhancing cGMP signaling, which is implicated in anti-inflammatory and anti-fibrotic processes; 2) preliminary clinical data indicating its safety in heart failure patients with concomitant CKD; 3) shared pathophysiological pathways between cardiac and renal fibrosis, including oxidative stress, inflammation, and maladaptive remodeling; and 4) the lack of systematic investigation into its direct renoprotective effects in CsA-induced nephropathy, a classic inflammatory–fibrotic CKD model.

Modern drug discovery increasingly leverages computational approaches for hypothesis generation. Network pharmacology, by constructing system-level “drug–target–disease” networks, facilitates the prediction of polypharmacological effects and underlying mechanisms, moving beyond the conventional “one drug–one target” dogma (18, 19). This approach is particularly apt for vericiguat, given that its upstream sGC/cGMP action likely influences broad downstream networks. However, associations from such predictions or observational studies are prone to confounding and reverse causation. Mendelian randomization (MR) strengthens causal inference using genetic variants as instrumental variables, mimicking randomized allocation to assess the causal effect of an exposure (e.g., drug target gene expression) on an outcome (e.g., CKD risk) (20, 21). The confluence of network pharmacology (for hypothesis generation) and MR (for causal validation) embodies a powerful, cutting-edge paradigm for *in silico* drug repurposing and mechanistic deconvolution.

Therefore, this study was designed to implement an integrative strategy elucidating the therapeutic potential and mechanism of vericiguat in CsA-induced CKD. We first applied network pharmacology to predict vericiguat’s core targets in CKD, focusing preliminary insights on the NF-κB/TGF-β1 axis. We then innovatively employed large-scale human Genome-Wide Association Study/Studies (GWAS) data in a two-sample MR framework to genetically validate the causal relationship between sGC stimulation and CKD risk. Finally, we conducted rigorous *in vivo* and *in vitro* functional validation using a CsA-induced mouse model and human renal tubular cells to confirm the renoprotective efficacy of vericiguat and definitively establish its anti-inflammatory and anti-fibrotic actions via the precise modulation of the NF-κB/TGF-β1 axis (study design summarized in **Supplementary Figure 1**). Our work not only provides a novel scientific foundation for repurposing vericiguat but also highlights the utility of combining computational and experimental biology in advancing therapeutic discovery for CKD.

## 2 Materials and methods

### 2.1 Materials

#### 2.1.1 Databases and software

The following databases were used: DrugBank (https://go.drugbank.com/), SwissTarget (http://www.Swisstargetprediction.ch/), Super-PRED (https://prediction.charite.de/index), GEO (https://www.ncbi.nlm.nih.gov/geo/), PubChem (https://pubchem.ncbi.nlm.nih.gov/), UniProt (https://www.uniprot.org/), TTD (https://db.idrblab.net/ttd/), GeneCards (https://www.genecards.org/), OMIM (https://www.omim.org/), STRING (https://cn.String-db.org/), RCSB PDB (https://www.rcsb.org/), DAVID (https://davidbioinformatics.nih.gov/), Metacpae (https://metascape.org/), OpenGWAS (https://opengwas.io), and Bioinformatics (https://www.bioinformatics.com.cn/).

The following software applications were used: Cytoscape 3.9.1, AutoDockTools 1.5.7, Maestro 14.3, Ligplus^+^ 2.2.9, Pymol 3.0.3, R/Rstudio 4.5.1, TwoSampleMR 0.6.6, and MR-PRESSO 1.0.

#### 2.1.2 Animals

All animal procedures were approved by the Institutional Animal Care and Use Committee of Shihezi University (Approval No. A2025-841) and conducted in accordance with relevant guidelines. Sixty-five male C57BL/6J mice aged 4–6 weeks (Specific Pathogen Free (SPF) grade, 18 ± 2 g) were sourced from Beijing SPF Biotechnology Co., Ltd. (Beijing, China). Mice, Shihezi, Xinjiang Uygur Autonomous Region, China, were housed under standard conditions (22°C–26°C, 50%–70% humidity) with *ad libitum* access to food and water.

#### 2.1.3 Cells

The human renal proximal tubular epithelial cell line (HK-2) was obtained from Procell (Wuhan, China) and authenticated by Short Tandem Repeat (STR) profiling. The Research Resource Identifier (RRID) for HK-2 is “CVCL_0302”.

#### 2.1.4 Drugs and reagents

Clinical formulations of vericiguat (Victhor) and cyclosporine A (Xinsaisping) were used for *in vivo* studies. For *in vitro* work, cyclosporine A (HY-B0579) and vericiguat (HY-16774) were purchased from MedChemExpress (Monmouth Junction, New Jersey, USA).

### 2.2 Methods

#### 2.2.1 Integrated computational analyses: network pharmacology and Mendelian randomization

##### 2.2.1.1 Vericiguat target identification

Putative targets of vericiguat were identified by integrating three databases. Initial targets were retrieved from DrugBank. Subsequently, the SMILES notation of vericiguat (obtained from PubChem) was used to predict targets via SwissTargetPrediction and Super-PRED. All targets were standardized and deduplicated using UniProt.

##### 2.2.1.2 CKD target screening and intersection

Disease-associated targets for CKD were sourced from GeneCards (relevance score ≥30, excluding RNA genes), OMIM, and TTD using the keyword “chronic kidney disease”. The union of targets from these databases constituted the CKD target set. The intersection between the vericiguat target set and the CKD target set was defined as the potential therapeutic targets for vericiguat in CKD, visualized using a Venn diagram.

##### 2.2.1.3 Drug–target–disease network construction

The overlapping targets between vericiguat and CKD were used to construct a “drug–target–disease” interaction network using Cytoscape 3.9.1.

##### 2.2.1.4 Protein–protein interaction network and core target analysis

The overlapping targets were imported into the STRING database to generate a protein–protein interaction (PPI) network (species: *Homo sapiens*, confidence score > 0.8, hiding disconnected nodes). Core targets within the PPI network were identified using three Cytoscape plugins: 1) CytoNCA, performing topological analysis based on betweenness, closeness, degree, eigenvector, local average connectivity, and network centralities (22); 2) MCODE, identifying densely connected regions (parameters: Degree Cutoff = 2, Node Score Cutoff = 0.6, K-Core = 2) (23); and 3) CytoHubba, applying the Maximal Clique Centrality (MCC) algorithm (24). The consensus core targets identified by all three methods were considered the most critical.

##### 2.2.1.5 Data sources for Mendelian randomization

Genetic instruments for vericiguat’s target genes (exposure) were defined as *cis*-expression quantitative trait loci (*cis*-eQTLs), with summary statistics sourced from the IEU OpenGWAS database. Genetic associations for CKD (outcome) were obtained from three independent European-ancestry GWAS summary datasets within IEU OpenGWAS (IDs: ebi-a-GCST008026, ebi-a-GCST003374, and finngen_R12_N14_CHRONKIDNEYDIS).

##### 2.2.1.6 Two-sample Mendelian randomization analysis

A two-sample MR framework was employed to assess the causal relationship between the genetically predicted expression of vericiguat’s target genes and CKD risk. Instrumental variables (IVs) were selected as Single Nucleotide Polymorphisms (SNPs) significantly associated with exposure (p < 5 × 10^−6^) after clumping for linkage disequilibrium (r^2^ < 0.001 within a 10,000-kb window) using a European reference panel. IV strength was assessed using the F-statistic (F > 10). SNPs associated with the outcome (p < 1 × 10^−5^) were excluded to mitigate potential pleiotropy, and the Steiger test confirmed the correct causal direction. The primary analysis used the inverse variance weighted (IVW) method, supplemented by MR-Egger, weighted median, simple mode, and weighted mode approaches. Heterogeneity was assessed using Cochran’s Q test, and horizontal pleiotropy was evaluated using the MR-Egger intercept test. MR-PRESSO was used to identify and remove outlier SNPs, and leave-one-out sensitivity analysis was performed. Analyses were conducted using the “TwoSampleMR” and “MR-PRESSO” R packages (25).

##### 2.2.1.7 Integration of network pharmacology and MR targets

The core targets from network pharmacology (MCC algorithm) were integrated with the significant MR genes from the three CKD GWAS datasets. The intersection formed a final, high-confidence core gene set. The overlap was visualized using Venn diagrams and network maps in Cytoscape.

##### 2.2.1.8 Functional enrichment analysis

Gene Ontology (GO) enrichment analysis (biological process, cellular component, and molecular function) for the core gene set was performed using DAVID. Kyoto Encyclopedia of Genes and Genomes (KEGG) pathway enrichment was conducted using Metascape. Significance was defined by a false discovery rate (FDR) <0.05 (hypergeometric test). KEGG analysis was additionally performed and visualized using the ClueGO plugin in Cytoscape (p-value <0.05).

##### 2.2.1.9 Validation in human CKD transcriptomic data

The human CKD transcriptomic dataset GSE37171 was downloaded from the GEO database, comprising 20 healthy controls and 63 end-stage renal disease patients. After normalization, differential expression analysis was performed against the core gene set (|logFC| ≥ 1.5, adjusted p-value <0.05). Results were visualized as volcano plots and heatmaps (26).

##### 2.2.1.10 Molecular docking

Molecular docking was performed between vericiguat (ligand) and the five high-confidence core target proteins (receptors) using AutoDock Vina. The 3D structures of the proteins were obtained from the RCSB PDB, and the vericiguat structure was sourced from PubChem. Docking simulations were run 10 times for each pair. The conformation with the lowest binding energy was selected for visualization of 3D interactions (PyMOL) and 2D interaction diagrams (LigPlot^+^). A heatmap of all binding energies was generated.

##### 2.2.1.11 Molecular dynamics simulation

The two vericiguat–target complexes with the lowest binding energies (vericiguat–IKBKB and vericiguat–NFKB1) were subjected to 100-ns molecular dynamics (MD) simulations using the Desmond module in Schrödinger Suite (27). Each complex was solvated in an orthorhombic TIP3P water box, neutralized with 0.15 M Na^+^/Cl^−^ ions, and energy-minimized. Systems were equilibrated under NVT and NPT ensembles (50 ns each) with positional restraints on protein and ligand heavy atoms, followed by a 100-ns production run without restraints (300 K, 1 bar). Trajectories were analyzed for root mean square deviation (RMSD), root mean square fluctuation (RMSF), and specific protein–ligand interactions (hydrogen bonds, hydrophobic contacts, water bridges, π–π stacking).

#### 2.2.2 *In vivo* animal studies

##### 2.2.2.1 Model establishment, grouping, and drug administration

**Figure 1:**
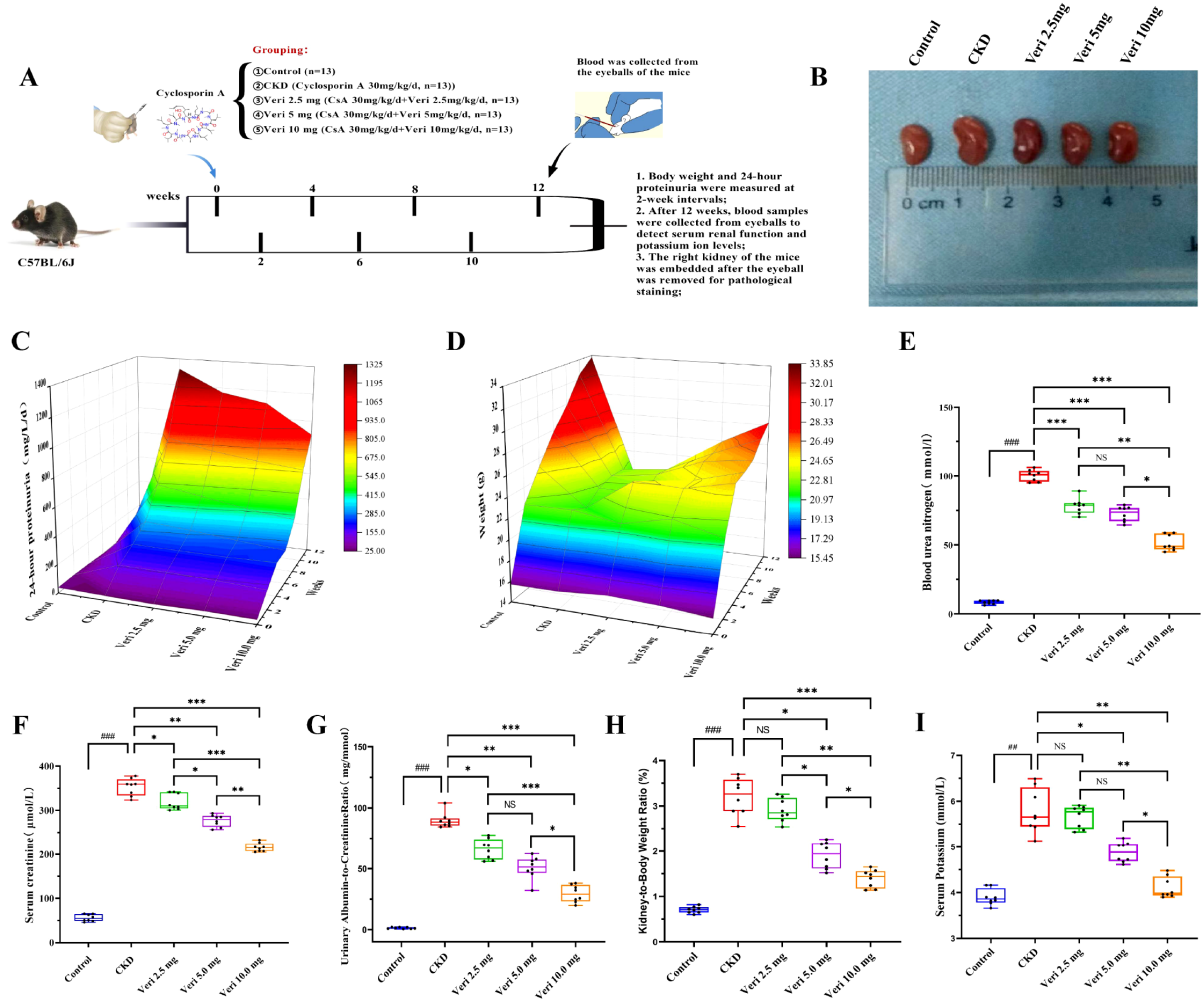
Vericiguat ameliorates renal function and pathological changes in a cyclosporine A (CsA)-induced chronic kidney disease mouse model (n = 8). **(A)** Schematic diagram of the experimental design for CsA-induced chronic kidney disease (CKD) and vericiguat treatment in mice. **(B)** Representative macroscopic images of kidneys from each group. **(C)** Levels of 24-h urinary protein measured at different timepoints. **(D)** Body weight changes over the 12-week experimental period. **(E–G)** Renal function biomarkers: serum urea nitrogen, serum creatinine, and urinary protein-to-creatinine ratio. **(H)** Kidney index (kidney weight/body weight ratio). **(I)** Serum potassium levels. The detailed numerical data corresponding to panels E–I (renal function and serum potassium measurements at the 12-week endpoint) are provided in **Supplementary Table 3.3**. Statistical analysis: data were analyzed using two-way ANOVA with Bonferroni *post-hoc* test for time-course data **(C, D)** and one-way ANOVA with Bonferroni *post-hoc* test for multiple group comparisons at endpoint **(E–I)**. Data are presented as mean ± SD; ^*^p < 0.05, ^**^p < 0.01, ^***^p < 0.001 vs. model group; ^*^p < 0.05, ^**^p < 0.01 vs. low-dose vericiguat group; ^#^p < 0.05, ^###^p < 0.001 vs. control group.

As schematized in **Figure 1A**, 65 male C57BL/6J mice (4–6 weeks old, 18 ± 2 g), were acclimatized for 1 week and then randomly assigned to either a healthy control group (n = 13) fed a standard diet or a modeling group (n = 52) fed a standard diet for 12 weeks. Controls received saline by gavage. The model group received CsA (30 mg kg^−1^ day^−1^ in olive oil) each morning. Based on human-to-mouse dose conversion, the modeling group was further divided into three vericiguat treatment groups receiving daily afternoon gavage of low-dose (2.5 mg/kg), medium-dose (5 mg/kg), or high-dose (10 mg/kg) vericiguat dissolved in saline while maintaining morning CsA administration. The doses of vericiguat were selected based on human-to-mouse dose conversion and prior preclinical studies demonstrating renal safety at comparable doses in rodents (17, 28). Body weight and 24-h urinary protein were monitored at weeks 0, 2, 4, 6, 8, 10, and 12. Post-intervention, blood was collected via ocular puncture, serum was separated, and kidneys were weighed to calculate the kidney index. A portion of the right renal cortex was fixed in 2.5% glutaraldehyde for transmission electron microscopy (TEM); the remainder was stored at −80°C. The left kidney was partly fixed in 4% paraformaldehyde for paraffin sectioning and partly snap-frozen for cryosectioning.

##### 2.2.2.2 Assessment of urinary protein, renal function, and serum potassium

At weeks 0, 2, 4, 6, 8, 10, and 12, mice were individually housed in metabolic cages for 24-h urine collection. At the endpoint (after 12 weeks of intervention), blood samples were collected via ocular puncture immediately before euthanasia. Serum samples were analyzed using commercial kits (Jiancheng Bioengineering, Nanjing, China) to measure urinary protein (Coomassie Brilliant Blue (CBB) method), serum creatinine (enzymatic), blood urea nitrogen (enzymatic), serum potassium (microplate), and urinary protein-to-creatinine ratio (CBB/enzymatic). Optical density (OD) values were converted to concentrations via standard curves. Three-dimensional color maps of 24-h urinary protein and body weight changes were generated using the Origin software.

##### 2.2.2.3 ELISA for renal inflammatory and fibrotic markers

Approximately 20–25 mg of right kidney tissue was homogenized. The levels of Interleukin-6 (IL-6), Tumor Necrosis Factor-α (TNF-α), α-Smooth Muscle Actin (α-SMA), and Epithelial-Cadherin (E-Cadherin) were quantified using mouse-specific ELISA kits (Jianglai Biotechnology, Shanghai, China) per the manufacturer’s protocol. Absorbance at 450 nm was measured (BioTek, Winooski, Vermont, USA), and concentrations were derived from standard curves.

##### 2.2.2.4 Renal histopathology

Left kidney tissues were fixed in 4% paraformaldehyde, paraffin-embedded, and sectioned at 3 μm for H&E, Masson’s trichrome, Sirius red, and Periodic Acid–Schiff (PAS) staining. Pathological changes in tubules, interstitium, and glomeruli were examined via light microscopy (ZEISS, Jena, Thuringia, Germany). Tubular luminal nuclei were quantified (29), and collagen deposition areas were measured using ImageJ.

##### 2.2.2.5 Transmission electron microscopy

Right renal cortex samples were fixed in 2.5% glutaraldehyde, post-fixed in 1% osmium tetroxide, dehydrated, and embedded in 812 resin. Ultrathin sections were stained with uranyl acetate and lead citrate and imaged under a transmission electron microscope (Leica, Vienna, Austria). Mitochondrial aspect ratio and cristae density were analyzed using ImageJ (30).

##### 2.2.2.6 DHE staining for renal reactive oxygen species

Left kidney cryosections (7 μm) were stained using a Dihydroethidium (DHE) Superoxide Anion Detection Kit (Beyotime, Shanghai, China). Fluorescence was captured via inverted fluorescence microscopy (Nikon, Tokyo, Japan), and relative intensity in tubules and interstitium was quantified using ImageJ.

##### 2.2.2.7 Immunofluorescence for renal inflammatory and fibrotic proteins

Paraffin sections were permeabilized, blocked, and incubated overnight with antibodies against IL-6, TNF-α, α-SMA, and E-Cadherin (1:200; Nos. 26404-1-AP, 17590-1-AP, 67735-1-Ig, and 20874-1-AP; Proteintech, Wuhan, China). After incubation with CoraLite594-conjugated (1:100; No. SA00013-3; Proteintech, Wuhan, China) secondary antibody, sections were counterstained with 4',6-diamidino-2-phenylindole (DAPI) and imaged. Fluorescence intensity was quantified using ImageJ.

##### 2.2.2.8 qRT-PCR for NF-κB/TGF-β1 axis gene expression

Total RNA was extracted from approximately 20–30 mg of right kidney tissue using an RNA extraction kit (OMEGA, Basel, Switzerland). cDNA was synthesized using a RevertAid RT Reverse Transcription Kit (Thermo, Waltham, Massachusetts, USA). qRT-PCR was performed on a real-time PCR system (Dongsheng Innovation, Beijing, China) using SYBR Green reagents (Servicebio, Wuhan, China). Primer sequences are listed in **Supplementary Table 4-1**. GAPDH was used as the endogenous reference gene, and relative mRNA expression was calculated using the 2^−ΔΔCt^ method, with the control group as the calibrator.

##### 2.2.2.9 Western blotting for NF-κB/TGF-β1 axis protein expression

Approximately 20–30 mg of renal tissue was lysed in Radioimmunoprecipitation assay (buffer) (RIPA) buffer and centrifuged (4°C, 12,000 rpm, 30 min), and protein concentration was determined using the bicinchoninic acid (BCA) method. Proteins were denatured, separated by electrophoresis, transferred to membranes, and blocked. Membranes were incubated overnight at 4°C with primary antibodies (1:1,000 dilution) against Nuclear Factor Kappa B Subunit p65 (NF-κB p65), Phosphorylated Nuclear Factor Kappa B Subunit p65 (NF-κB P-p65), Inhibitor of Kappa B Alpha (IκBα), Phosphorylated Inhibitor of Kappa B Alpha (p-IκBα), Smad2/3, p-Smad2/3, Smad4, Smad7, TGF-β1, Activin Receptor-Like Kinase 5 (ALK5) (Nos. WL01980, WL02169, WL01936, WL02495, WL01520, WL02305, WL02049, WL02975, WL03150, and WL02193; Wanlei Bio, Shenyang, China), Inhibitor of Nuclear Factor Kappa B Kinase Subunit Beta (IKKβ), and Phosphorylated Inhibitor of Nuclear Factor Kappa B Kinase Subunit Beta (p-IKKβ) (Nos. AF6009 and AF3013; Affinity, Cincinnati, Ohio, USA). After incubation with Horseradish Peroxidase (HRP)-conjugated secondary antibodies, blots were developed with Enhanced Chemiluminescence (ECL) reagent. Histone H3 or GAPDH (Nos. TA328045 and TA309157; Zhongshan Jinqiao, Beijing, China) was used as the loading control for nuclear or cytoplasmic proteins, respectively. Band density was quantified using the ImageJ software.

##### 2.2.2.10 Assessment of vericiguat monotherapy in healthy mice

To rigorously evaluate potential nephrotoxic or off-target systemic effects of vericiguat per se, an additional control cohort of healthy mice (n = 8) was administered vericiguat alone (10 mg kg^−1^ day^−1^, p.o.) for 12 weeks, concurrent with the main experimental groups. This group received the vehicle (olive oil) in the morning and vericiguat (10 mg/kg) in the afternoon, following the same dosing schedule and volume as the treatment groups.

#### 2.2.3 *In vitro* cell studies

##### 2.2.3.1 Cytotoxicity and IC_50_ assessment

The human renal proximal tubular epithelial cell line (HK-2) was selected for *in vitro* studies because renal tubular epithelial cells are primary targets of CsA-induced injury and play a central role in initiating and perpetuating inflammation and fibrosis in CKD. HK-2 cells retain key characteristics of proximal tubules and are widely used to model drug-induced nephrotoxicity, oxidative stress, and fibrotic responses. HK-2 cells were maintained in Dulbecco’s Modified Eagle Medium (DMEM) with 10% Fetal Bovine Serum (FBS) and 1% penicillin–streptomycin. To establish an *in vitro* inflammatory–fibrotic model, cells were treated with 5 μM CsA for 48 h. The CsA concentration and duration were selected based on previously established subtoxic inflammatory–fibrotic models, which induce marked pathological responses without significant cytotoxicity (31, 32). Vericiguat (Veri) was dissolved in Dimethyl Sulfoxide (DMSO) (final concentration ≤0.1%). Experimental groups included the following: control, CsA (5 μM), CsA + Veri low (8 μM), CsA + Veri medium (17 μM), and CsA + Veri high (25 μM). Cells were serum-starved for 12 h prior to CsA exposure; all treatments lasted 48 h.

##### 2.2.3.2 Vericiguat-alone treatment groups

To evaluate the independent effects of vericiguat on oxidative stress, inflammation, and fibrosis, additional control groups were included in which HK-2 cells were treated with vericiguat alone at low (8 μM), medium (17 μM), and high (25 μM) doses for 48 h, without CsA pre-treatment. These groups were subjected to 2′,7′-Dichlorodihydrofluorescein Diacetate (DCFH-DA) staining for reactive oxygen species (ROS) detection; ELISA for IL-6, TNF-α, α-SMA, and E-Cadherin, as described (in Sections 2.2.3.3 and 2.2.2.3), and Cell Counting Kit-8 (CCK-8) were used to assess the toxicity of vericiguat in the single-dose group at 48 h.

##### 2.2.3.3 Cytotoxicity and IC_50_ assay

HK-2 cells (1 × 10^4^ cells/well) in 96-well plates were treated with vericiguat (0–140 μM) for 24 or 48 h. Viability was assessed using CCK-8. Absorbance at 450 nm was measured, and cell viability and inhibition rates were calculated as follows:


Cell survival rate=[(experimental well−blank well)/(control well−blank well)]×100%,

Cell inhibition rate=[(control well−experimental well)/(control well−blank well)]×100%.

IC_50_ values and curves were generated from dose–response data.

##### 2.2.3.4 Intracellular ROS detection by DCFH-DA

HK-2 cells (2.5 × 10^5^ cells/well) in 24-well plates were treated as indicated. ROS levels were measured using a DCFH-DA assay kit (S0034S, Beyotime) per the manufacturer’s instructions. Fluorescence was visualized via inverted fluorescence microscopy, and mean fluorescence density was quantified using ImageJ.

##### 2.2.3.5 Immunofluorescence for inflammatory and fibrotic markers in HK-2 cells

HK-2 cells (2.5 × 10^5^ cells/well) grown on coverslips were fixed, permeabilized, blocked, and incubated overnight at 4°C with primary antibodies (1:200) against IL-6, TNF-α, α-SMA, P-NF-kB p65, and E-Cadherin. After washing, cells were incubated with CoraLite488-conjugated Goat Anti-Rabbit IgG(H+L) (SA00013-2, Proteintech), counterstained with DAPI, and imaged. Fluorescence intensity was analyzed using ImageJ.

##### 2.2.3.6 Western blotting in HK-2 cells

HK-2 cells (1.2 × 10^6^ cells/well) in 6-well plates were treated as described. Protein extraction and immunoblotting were performed as for renal tissues. Antibodies against NF-κB p65, P-p65, IκBα, p-IκBα, Smad2/3, P-Smad2/3, Smad4, Smad7, TGF-β1, ALK5, IKKβ, and p-IKKβ were used. Histone H3 and GAPDH served as loading controls.

##### 2.2.3.7 Lentiviral transduction for p65 modulation

**Figure 2:**
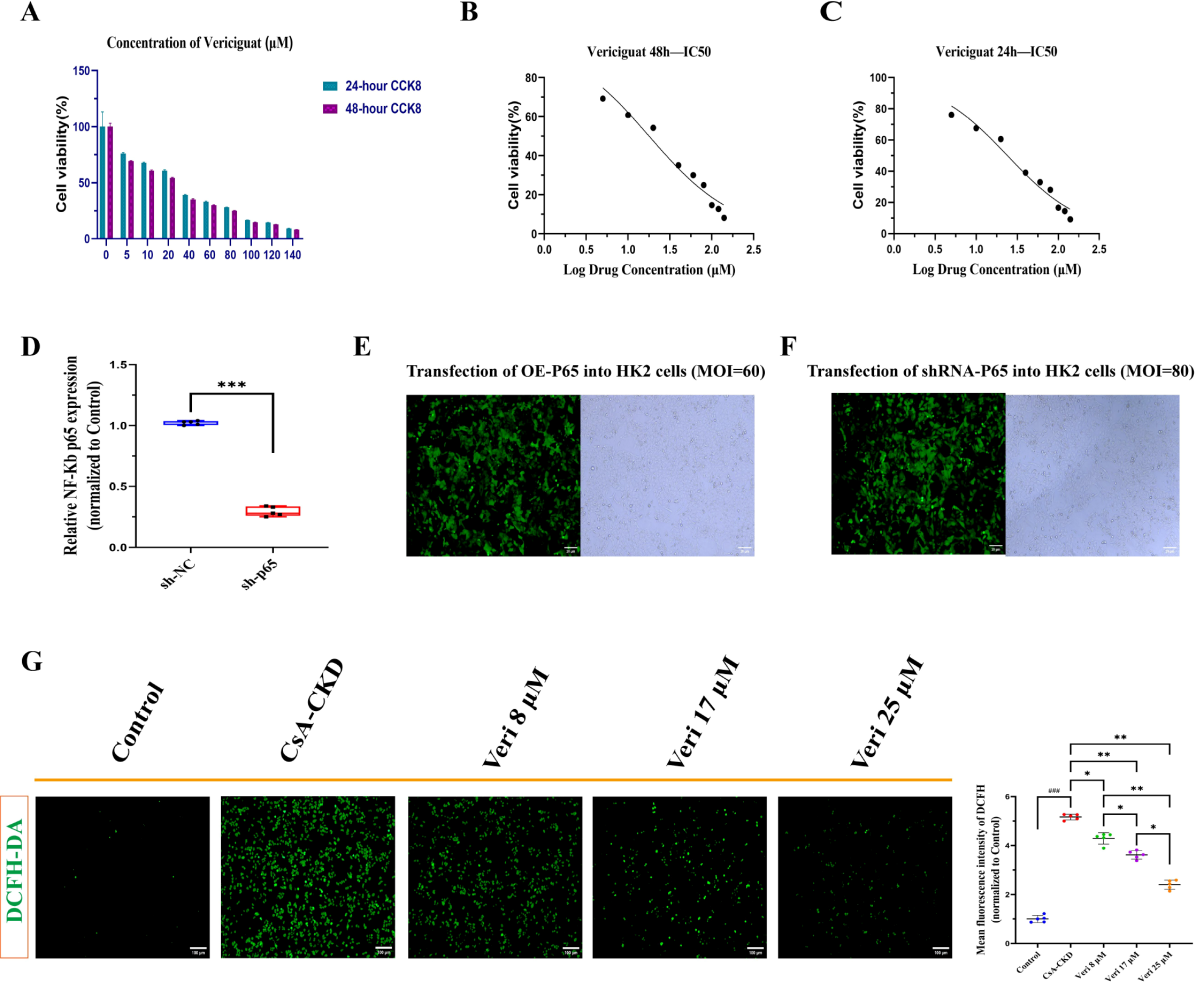
Cytotoxicity, lentiviral transduction efficiency, and oxidative stress in HK-2 cells treated with vericiguat. **(A–C)** Cytotoxicity and IC_50_ determination of vericiguat in HK-2 cells (n = 6): **(A)** cell viability after 24- and 48-h treatments with increasing concentrations of vericiguat (0–140 µM). **(B, C)** IC_50_ curves and values derived from cell viability data at 24 and 48 h. **(D)** qRT-PCR validation of p65-knockdown efficiency in HK-2 cells stably transduced with shRNA-p65 lentivirus (n = 5). Data are presented as mean ± SD; ^***^p < 0.001 vs. shRNA-NC group. **(E, F)** Optimization of lentiviral transduction conditions: MOI values for OE-p65 (MOI = 60) and shRNA-p65 (MOI = 80) were selected based on transfection efficiency and minimal cytotoxicity (scale bar, 50 μm). **(G)** Quantitative analysis of intracellular reactive oxygen species (ROS) levels detected via DCFH-DA staining in HK-2 cells under cyclosporine A (CsA) and vericiguat treatment (n = 5; scale bar, 50 μm). Statistical analysis: one-way ANOVA with Bonferroni *post-hoc* test was used for multiple group comparisons **(A, D, G)**. Data are presented as mean ± SD; ^*^p < 0.05, ^**^p < 0.01, ^***^p < 0.001 vs. CsA group; ^*^p < 0.05, ^**^p < 0.01 vs. low-dose vericiguat group; ^#^p < 0.05, ^###^p < 0.001 vs. control group.

Building on our earlier screening that pinpointed the NF-κB/TGF-β1 axis—and specifically p65 as a critical node—as central to vericiguat’s action in CKD, lentiviral constructs were designed for p65 knockdown (shRNA-p65) and overexpression (OE-p65) (Genomeditech, Suzhou, China). To achieve robust transfection with minimal cytotoxicity, Multiplicity of Infection (MOI) values were optimized in pilot studies: MOI = 60 for OE-p65 and MOI = 80 for shRNA-p65 (**Figures 2E, F**). Stable HK-2 cell lines were generated via lentiviral transduction followed by selection with puromycin (5 μg/mL). The experimental groupings were as follows:

- OE-NC, OE-NC + CsA, OE-NC + CsA + Veri (8 μM), OE-NC + CsA + Veri (25 μM), OE-p65, OE-p65 + CsA, OE-p65 + CsA + Veri (8 μM), and OE-p65 + CsA + Veri (25 μM); and
- shRNA-NC, shRNA-NC + CsA, shRNA-NC + CsA + Veri (8 μM), shRNA-NC + CsA + Veri (25 μM), shRNA-p65, shRNA-p65 + CsA, shRNA-p65 + CsA + Veri (8 μM), and shRNA-p65 + CsA + Veri (25 μM).

##### 2.2.3.8 qRT-PCR validation of p65 knockdown

Total RNA from stable shRNA-p65 HK-2 cells was reverse-transcribed and analyzed via qRT-PCR using SYBR Green. Primers are listed in **Supplementary Table 4-1**. GAPDH was the reference gene, and relative expression was calculated using the 2^−ΔΔCt^ method versus shRNA-NC.

##### 2.2.3.9 Western blotting after p65 knockdown

Stable shRNA-p65 HK-2 cells (1.2 × 10^6^ cells/well) were treated as designated. The expression of NF-κB/TGF-β1 axis proteins and inflammatory/fibrotic markers (IL-6, TNF-α, α-SMA, and E-Cadherin) was assessed via Western blotting.

##### 2.2.3.10 Western blotting after p65 overexpression

Stable OE-p65 HK-2 cells (1.2 × 10^6^ cells/well) were treated as indicated. The protein levels of NF-κB/TGF-β1 pathway components and inflammatory/fibrotic markers were analyzed via Western blotting.

### 2.3 Statistical analysis

Data are presented as mean ± standard deviation (X ± SD). The Shapiro–Wilk test was used to assess normality. One-way analysis of variance (ANOVA) was applied for multiple group comparisons. For time-course experiments involving repeated measurements, two-way ANOVA with Bonferroni *post-hoc* testing was utilized. Student’s t-test was employed for comparisons between two groups. All statistical analyses were conducted using SPSS 27.0, while GraphPad Prism 9.0 and Origin 2024 were used for graph generation. A p-value <0.05 was considered statistically significant (^*/#^<0.05,^**/##^<0.01, and^***/###^<0.001).

## 3 Results

### 3.1 Integrated network pharmacology and Mendelian randomization identify core targets of vericiguat in CKD

#### 3.1.1 Target profiling of vericiguat

We systematically identified vericiguat targets by integrating three pharmacological databases. Initial screening using DrugBank yielded two direct targets. Subsequent structure-based prediction via SwissTarget and SuperPred, using SMILES identifiers from PubChem, provided 150 additional candidates. After UniProt-based normalization and deduplication, a final set of 135 high-confidence targets was retained.

#### 3.1.2 CKD-related target screening

**Figure 3:**
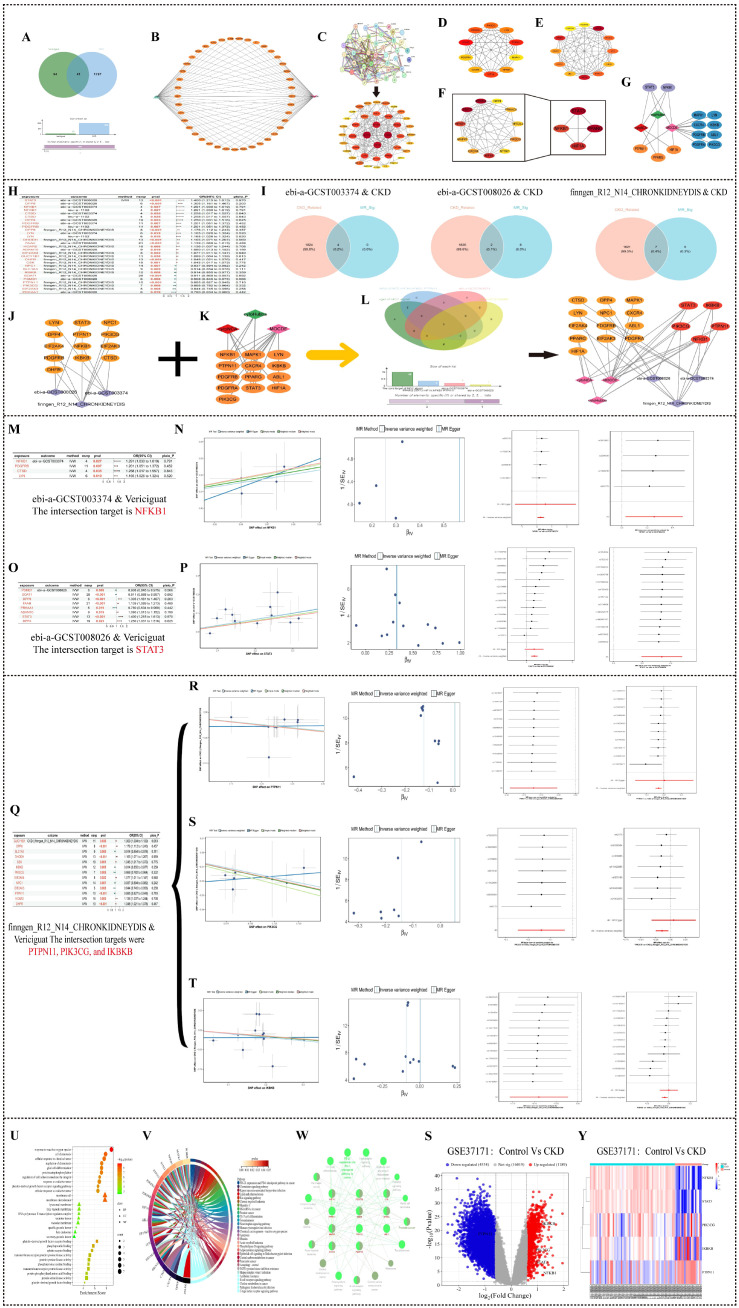
Identification of core targets and mechanisms of vericiguat for chronic kidney disease treatment via network pharmacology and Mendelian randomization. **(A)** Venn diagram showing the 42 overlapping targets between vericiguat and chronic kidney disease. **(B)** “Drug–target–disease” interaction network. **(C–G)** Screening of core targets from the protein–protein interaction network: **(C)** PPI network; **(D)** subnetwork identified by CytoNCA plugin; **(E)** core targets screened by CytoHubba using Maximal Clique Centrality (MCC) algorithm; **(F)** key module identified by MCODE plugin; **(G)** core targets STAT3 and NFKB1 common to all three methods. **(H)** Forest plot of Mendelian randomization results between drug target genes and three chronic kidney disease (CKD) GWAS datasets. **(I)** Venn diagram illustrating the overlap between core targets from network pharmacology and significant genes from Mendelian randomization. **(J)** Integrated “Network Pharmacology–Mendelian Randomization” target network. **(K)** Venn diagram identifying the five most core targets: STAT3, PTPN11, PIK3CG, IKBKB, and NFKB1. **(L)** Mendelian randomization scatter plot, SNP forest plot, leave-one-out forest plot, and funnel plot of MR analysis for NFKB1 (exposure) and ebi-a-GCST003374 (outcome). **(M)** Mendelian randomization scatter plot, SNP forest plot, leave-one-out forest plot, and funnel plot of MR analysis for STAT3 (exposure) and ebi-a-GCST008026 (outcome). **(N–P)** Mendelian randomization scatter plot, SNP forest plot, leave-one-out forest plot, and funnel plot of MR analysis for PTPN11, PIK3CG, and IKBKB (exposures) with finngen_R12_N14_CHRONKIDNEYDIS (outcome). **(Q–S)** Validation of core gene expression in the GEO dataset GSE37171: **(Q)** volcano plot of differentially expressed genes; **(R)** heatmap displaying expression patterns of core genes in healthy controls versus CKD patients; **(S)** box plots showing expression levels of core genes. **(T–V)** Functional enrichment analysis of core targets: **(T)** bubble plot of GO enrichment analysis (biological process, cellular component, and molecular function); **(U)** chord plot of Kyoto Encyclopedia of Genes and Genomes (KEGG) pathway enrichment analysis; **(V)** network of KEGG pathways analyzed using the ClueGO plugin.

CKD-associated genes were collated from GeneCards (score ≥ 30), OMIM, and TTD, yielding 1,265, 384, and 181 targets, respectively. Intersection with the 135 vericiguat targets revealed 42 shared genes, as visualized in a Venn diagram (**Figure 3A**).

#### 3.1.3 Construction of the “drug–target–disease” network

A bipartite network linking vericiguat, the 42 overlapping targets, and CKD was constructed using Cytoscape, illustrating potential mechanistic connections (**Figure 3B**).

#### 3.1.4 Protein–protein interaction network and core target identification

STRING-derived PPI networks of the 42 targets were analyzed using three Cytoscape plugins: CytoNCA (topological filtering), CytoHubba (MCC algorithm), and MCODE (module detection). Integration of results identified 13 core targets, with Signal Transducer and Activator of Transcription 3 (STAT3) and NFKB1 consistently highlighted across all methods (**Figures 3C–G**).

#### 3.1.5 GWAS data curation

We used 135 vericiguat target genes as exposures, with eQTL data sourced from IEU OpenGWAS. Three European CKD GWAS datasets served as outcomes: ebi-a-GCST008026, ebi-a-GCST003374, and finngen_R12_N14_CHRONKIDNEYDIS (sample sizes, 20,920–493,235; **Supplementary Table 1-1**).

#### 3.1.6 Causal inference via Mendelian randomization

MR analysis was performed using the TwoSampleMR package. Among the 135 drug target genes, 112 had GWAS data, and 93 had more than three IVs. A total of 25 significant MR results (p < 0.05) were identified across the three outcome datasets. Forest plots were used to display these results (**Figure 3H**). Overlap analysis between MR-significant genes and network pharmacology core targets identified key genes such as NFKB1, STAT3, Phosphoinositide 3-Kinase Gamma (PIK3CG), IKBKB, and PTPN11 (**Figure 3I**). For NFKB1 (exposure) and ebi-a-GCST003374 (outcome), the IVW method showed a significant positive effect (OR > 1), indicating that NFKB1 is a risk factor for CKD. Sensitivity analyses showed no heterogeneity or pleiotropy (**Figures 3L–N**). Similar analyses were performed for STAT3 (with ebi-a-GCST008026) and PTPN11, PIK3CG, and IKBKB (with finngen_R12_N14_CHRONKIDNEYDIS), confirming their causal roles (**Figures 3M–T**). Detailed MR analysis results for all target genes are provided in **Supplementary Tables 1** and **2**.

#### 3.1.7 Sensitivity analyses for Mendelian randomization

Heterogeneity, pleiotropy, and leave-one-out validation: NFKB1 (exposure) and ebi-a-GCST003374 (outcome)—MR analysis using the IVW random-effects model revealed a significant causal effect of NFKB1 on CKD risk (OR > 1; p < 0.05), supported by four instrumental SNPs. Forest plot visualization confirmed a consistent risk-increasing effect (**Figure 3N**). Sensitivity analyses robustly supported these findings: Cochran’s Q test indicated no significant heterogeneity (p > 0.05), justifying the use of a fixed-effects IVW model. MR-Egger regression detected no evidence of horizontal pleiotropy (intercept p > 0.05). Furthermore, leave-one-out analysis demonstrated that the causal estimate remained stable upon iterative removal of individual SNPs, confirming the result’s robustness.

STAT3 (exposure) and ebi-a-GCST008026 (outcome): The IVW random-effects model indicated a significant positive causal relationship between STAT3 expression and CKD risk (OR > 1; p < 0.05) based on 13 instrumental SNPs. The forest plot illustrated an overall positive effect size (**Figure 3O**). Sensitivity analyses affirmed reliability: heterogeneity was absent (p > 0.05), no horizontal pleiotropy was detected (MR-Egger intercept p > 0.05), and leave-one-out analysis confirmed the stability of the causal estimate.

PTPN11, PIK3CG, IKBKB (exposures), and finngen_R12_N14_CHRONKIDNEYDIS (outcome): MR analyses for these targets were conducted using the IVW random-effects model. PIK3CG and IKBKB were identified as risk factors (OR > 1; p < 0.05), whereas PTPN11 was a protective factor (OR < 1; p < 0.05). Forest plots visualized these associations (**Figures 3R–T**). For all three genes, sensitivity analyses confirmed the absence of heterogeneity (p > 0.05) and horizontal pleiotropy (p > 0.05). Leave-one-out analyses further validated the stability and reliability of the causal inferences (**Supplementary Tables 1-3**, **1-4**).

#### 3.1.8 Construction of the “network pharmacology–Mendelian randomization” target network

The 13 core targets from network pharmacology and the 13 MR-significant targets were integrated, resulting in 19 non-redundant targets. The five most central targets—STAT3, PTPN11, PIK3CG, IKBKB, and NFKB1—were identified and visualized using Cytoscape and Venn diagrams (**Figures 3K, L**).

#### 3.1.9 Functional enrichment of core targets

GO analysis of the 19 core genes revealed enrichment in biological processes such as “response to reactive oxygen species”, “cellular response to chemical stress”, and “cell chemotaxis”; cellular components including “membrane raft” and “lysosomal membrane”; and molecular functions such as “protein tyrosine kinase activity” (**Figure 3U**). KEGG analysis highlighted pathways like “Pathways in cancer”, “Chemokine signaling pathway”, and “Ras signaling pathway”, many of which are related to inflammation, fibrosis, and oxidative stress (**Figures 3V, W**).

#### 3.1.10 Transcriptomic validation in human CKD

Analysis of the GSE37171 dataset (20 healthy controls vs. 63 end-stage renal disease patients) showed that STAT3, PIK3CG, IKBKB, and NFKB1 were upregulated in CKD patients, while PTPN11 was downregulated, suggesting their roles in CKD progression (**Figures 3S–Y**).

#### 3.1.11 Molecular docking with core targets

**Figure 4:**
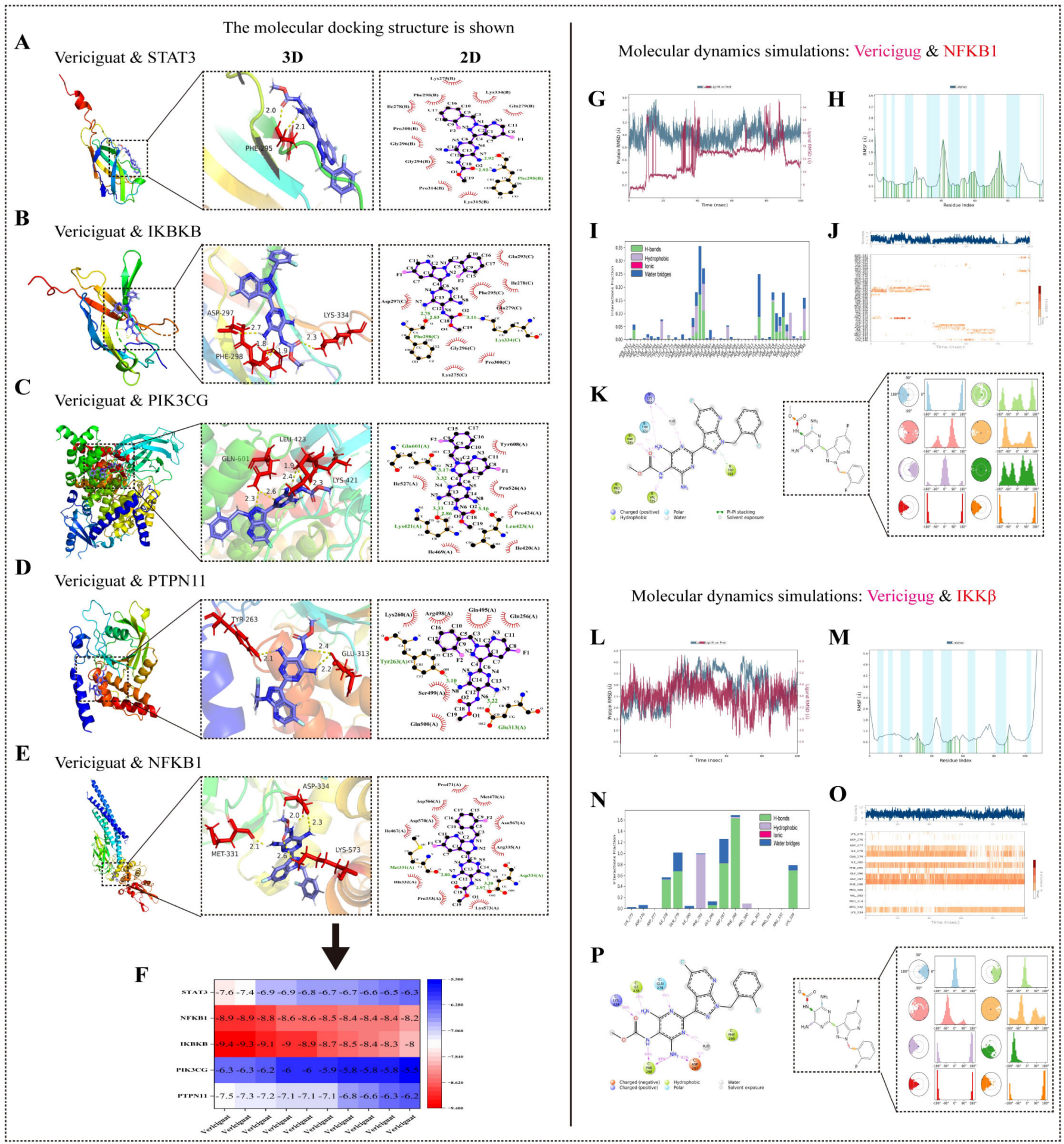
Molecular docking and dynamics simulation analysis of vericiguat with core targets NFKB1 and IKBKB. **(A–F)** Molecular docking results of vericiguat with the five core targets: **(F)** heatmap of docking binding energies between vericiguat and the five protein receptors. **(A–E)** The best docking pose 3D diagrams and corresponding 2D interaction diagrams of vericiguat with STAT3, PTPN11, PIK3CG, IKBKB, and NFKB1; 100-ns molecular dynamics simulation analysis of vericiguat with NFKB1 and IKBKB: **(G, L)** root mean square deviation of the protein–ligand complexes. **(H, M)** Root mean square fluctuation of protein residues. **(I, N)** Protein–ligand interaction fingerprints depicting interaction types and frequencies with specific residues over simulation time. **(J, O)** Energy contribution plots of binding site residues. **(K, P)** Detailed 2D interaction diagrams of the final simulated pose between the ligand and protein.

Vericiguat was docked with the five core targets. Binding energies below −5 kcal/mol indicated stable binding, with the lowest energies observed for IKBKB and NFKB1 (<−8 kcal/mol), suggesting strong inhibition of the NF-κB pathway (**Figures 4A–F**).

#### 3.1.12 Molecular dynamics simulations

To assess the binding stability and dynamic interaction profiles of vericiguat with the core targets NFKB1 and IKKβ, we performed 100-ns MD simulations for the lowest binding energy complexes identified in Section 3.1.10. Trajectories were analyzed for RMSD, RMSF, and specific protein–ligand interactions. For the vericiguat–NFKB1 complex, the RMSD plateaued after ~90 ns with minimal fluctuations, indicating conformational stability and binding equilibrium in the simulation’s later phase. RMSF analysis revealed moderate flexibility in residue regions 38–45 and 70–80, although overall fluctuations remained low (RMSF < 3 Å), suggesting that ligand binding did not induce major structural perturbations. Vericiguat engaged in multiple non-covalent interactions with NFKB1 Chain B residues TRP292, GLU293, PHE295, LYS315, VAL319, THR322, and TYR348, including hydrogen bonds, water bridges, and π–π stacking. Among these, LYS315, THR322, PHE295, and VAL319 sustained hydrogen bonding for >5% of the simulation time, while TRP292 contributed via stable π–π stacking. In the vericiguat–IKKβ complex, RMSD similarly stabilized after ~90 ns, reflecting system equilibrium. The RMSF profile indicated heightened local flexibility within segments 40–50, 70–85, and 85–95, potentially related to conformational adjustments upon ligand binding. Vericiguat formed a robust interaction network with IKKβ Chain C residues ILE278, GLN279, PHE295, ASP297, PHE298, and LYS334, dominated by hydrogen bonding. Notably, GLN279, ILE278, LYS334, PHE298, and ASP297 maintained hydrogen bond occupancy >30%, with ASP297 further stabilizing the complex via water bridges (**Figures 4G–P**). Molecular Mechanics/Generalized Born Surface Area (MM-GBSA) calculations confirmed high binding stability, with vericiguat–IKBKB and vericiguat–NFKB1 values of −44.51 and −44.58 kcal/mol, respectively (**Supplementary Table 2-1**), consistent with the strong binding affinities predicted by molecular docking.

### 3.2 *In vivo* efficacy of vericiguat in a CsA-induced CKD mouse model

#### 3.2.1 Vericiguat ameliorates proteinuria and body weight loss

CsA administration induced a significant increase in 24-h urinary protein excretion starting at week 4 compared to controls (p < 0.001; **Figure 1C**; **Supplementary Table 3-1**). Vericiguat treatment attenuated this increase in a dose- and time-dependent manner. Significant reduction in proteinuria versus the model group was observed after 8 weeks of treatment (p < 0.05), with medium and high doses showing superior efficacy to the low dose (p < 0.05), and the high dose being most effective (p < 0.05). Body weight progression was impaired in the model group from week 6 onward (p < 0.05; **Figure 1D**; **Supplementary Table 3-2**). Vericiguat treatment at medium and high doses, but not the low dose, significantly mitigated this weight loss (p < 0.05), demonstrating a dose-dependent protective effect on overall health.

#### 3.2.2 Vericiguat improves renal function and morphology

At the study endpoint (after 12 weeks of intervention), mice were euthanized, and blood samples were collected for assessment of renal function and electrolyte balance. As shown in **Figure 1B**, when the eyeball blood was taken from each group, and the bilateral kidneys were weighed, the external structure of the kidneys of each group was displayed. CsA-induced nephropathy was characterized by significantly elevated levels of blood urea nitrogen (BUN), serum creatinine (SCr), and the urinary protein-to-creatinine ratio (UPCR) (p < 0.001; **Figures 1E–G**; **Supplementary Table 3-3**). Vericiguat treatment dose-dependently ameliorated these functional impairments. The kidney index, indicative of compensatory hypertrophy, was significantly increased in the model group (p < 0.001; **Figure 1H**) and was effectively normalized by medium- and high-dose vericiguat (p < 0.05). Hyperkalemia, a complication of CKD, was also significantly elevated in the model group (p < 0.01; **Figure 1I**) and was dose-dependently attenuated by vericiguat, confirming its renal protective role without adverse effects on serum potassium.

#### 3.2.3 Vericiguat monotherapy exhibits no adverse renal effects in healthy mice

To rigorously exclude any potential nephrotoxic or systemic side effects of vericiguat itself, a cohort of healthy mice received vericiguat monotherapy (10 mg kg^−1^ day^−1^) for 12 weeks alongside the control and CsA-treated groups. Comprehensive assessment revealed that the vericiguat-alone treatment did not induce any significant alterations in renal function parameters (serum creatinine, BUN, and UPCR), serum potassium levels, or kidney index compared to the healthy control group (all p > 0.05; **Supplementary Figures 2A–D**; **Supplementary Table 3-4**). Similarly, ELISA analysis showed no marked changes in IL-6, TNF-α, α-SMA, or E-Cadherin expression upon vericiguat monotherapy (**Supplementary Figures 3E–H**). These data unequivocally demonstrate that the renoprotective effects observed in CsA-treated mice are attributable to vericiguat’s specific modulation of disease pathways rather than any confounding influence of drug-induced toxicity.

#### 3.2.4 Vericiguat suppresses renal inflammation and fibrosis

**Figure 5:**
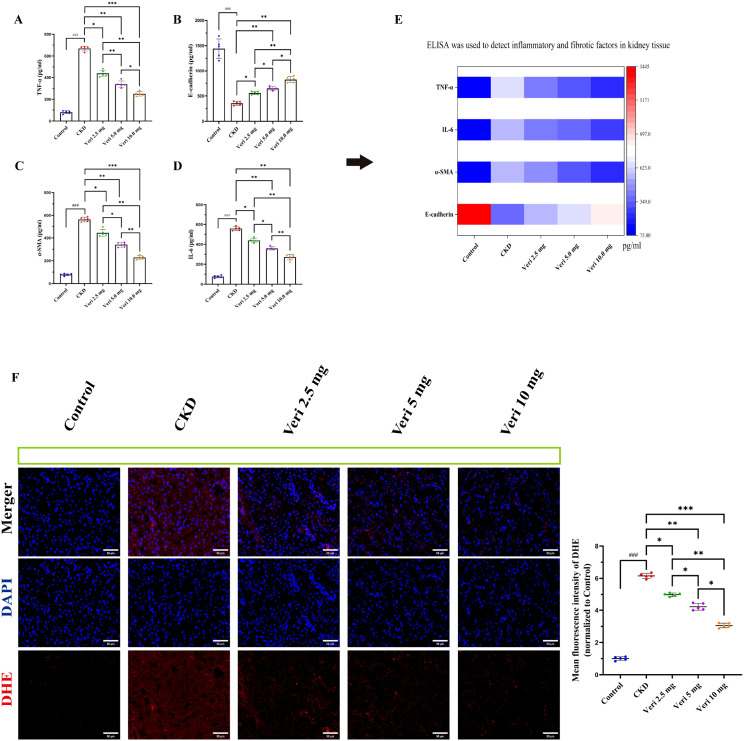
Vericiguat attenuates inflammation, fibrosis, and oxidative stress in cyclosporine A (CsA)-induced chronic kidney disease (CKD) mice (n = 5; scale bar, 50 μm). **(A–D)** ELISA analysis of renal tissue levels of IL-6, TNF-α, α-SMA, and E-Cadherin. **(E)** Heatmap summarizing expression levels of inflammatory and fibrotic markers across groups. **(F)** DHE staining and quantitative analysis of reactive oxygen species (ROS) in renal tissues. Statistical analysis: one-way ANOVA with Bonferroni *post-hoc* test was used for multiple group comparisons **(A–D, F)**. Data are presented as mean ± SD; ^*^p < 0.05, ^**^p < 0.01, ^***^p < 0.001 vs. model group; ^*^p < 0.05, ^**^p < 0.01 vs. low-dose vericiguat group; ^#^p < 0.05, ^###^p < 0.001 vs. control group.

ELISA analysis revealed a marked upregulation of pro-inflammatory (IL-6 and TNF-α) and pro-fibrotic (α-SMA) markers, alongside the downregulation of the epithelial integrity marker E-Cadherin, in model group kidneys (p < 0.001; **Figures 5A–D**). Vericiguat treatment significantly reversed these changes in a dose-dependent manner (p < 0.05), with the high dose exerting the most potent anti-inflammatory and anti-fibrotic effects (p < 0.05 vs. medium dose). A composite heatmap visually summarizes these improvements across treatment groups (**Figure 5E**).

#### 3.2.5 Vericiguat attenuates renal oxidative stress

Dihydroethidium staining showed a substantial increase in renal reactive oxygen species in the model group (p < 0.001; **Figure 5F**). Vericiguat treatment significantly quenched this oxidative burst in a dose-dependent manner (p < 0.05), with the high dose providing the strongest antioxidant effect (p < 0.05).

#### 3.2.6 Vericiguat mitigates renal histopathological damage

**Figure 6:**
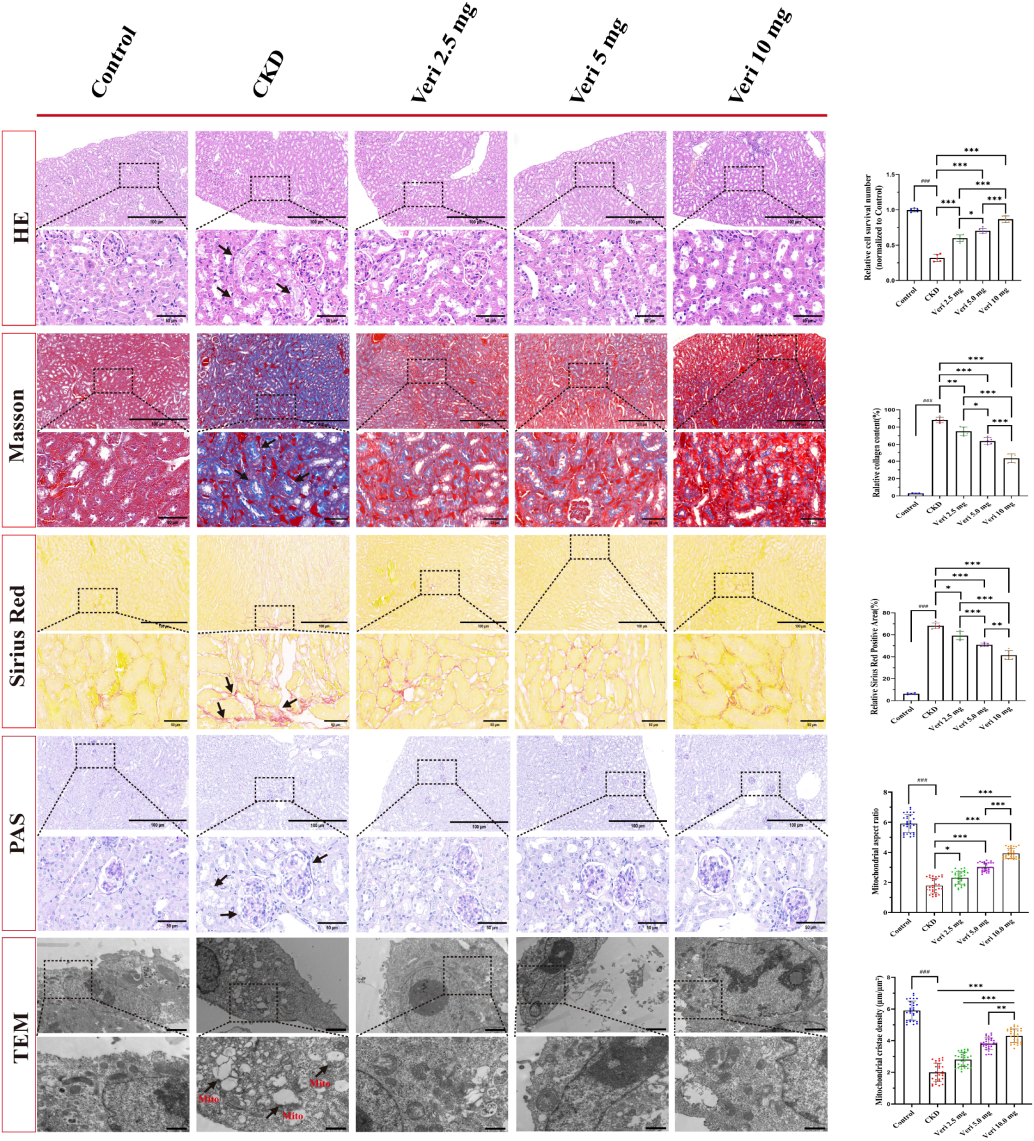
Histopathological and ultrastructural evaluation of renal tissues in cyclosporine A (CsA)-induced chronic kidney disease (CKD) mice treated with vericiguat (n = 5; scale bar, 100/50 μm, TEM; scale bar, 2/5 μm). Representative images of renal sections stained with H&E, Masson’s trichrome, Sirius red, and PAS, and transmission electron micrographs (TEM) of renal tubules. Quantitative analysis of relative nuclear count in H&E staining, collagen fiber area in Masson and Sirius red staining, mitochondrial aspect ratio, and cristae density in TEM. Statistical analysis: one-way ANOVA with Bonferroni *post-hoc* test was used for all quantitative comparisons. Data are presented as mean ± SD; ^*^p < 0.05, ^**^p < 0.01, ^***^p < 0.001 vs. model group; ^*^p < 0.05, ^**^p < 0.01 vs. low-dose vericiguat group; ^#^p < 0.05, ^###^p < 0.001 vs. control group.

Histological assessment (**Figure 6**) confirmed severe renal injury in the model group, featuring tubular dilation, vacuolization, nuclear dropout, interstitial inflammatory infiltration, and a significant decrease in relative nuclear count (p < 0.001, H&E). Masson’s trichrome and Sirius red staining revealed extensive collagen deposition (fibrosis) in the model group (p < 0.001), which was significantly reduced by vericiguat treatment in a dose-dependent manner (p < 0.01, p < 0.05). PAS staining indicated glomerular basement membrane thickening and brush border loss in model mice, changes that were subjectively improved by vericiguat.

#### 3.2.7 Vericiguat preserves mitochondrial ultrastructure

TEM (**Figure 6**) unveiled profound mitochondrial damage in model group tubules, characterized by swelling (increased aspect ratio) and cristae loss (decreased density) (p < 0.001). Vericiguat treatment dose-dependently preserved mitochondrial integrity, reducing the aspect ratio and increasing cristae density (p < 0.001), with the high dose being most effective (p < 0.01 vs. medium dose). These ultrastructural findings corroborated the light microscopy observations.

#### 3.2.8 Immunofluorescence confirms dose-dependent anti-inflammatory and anti-fibrotic effects

**Figure 7:**
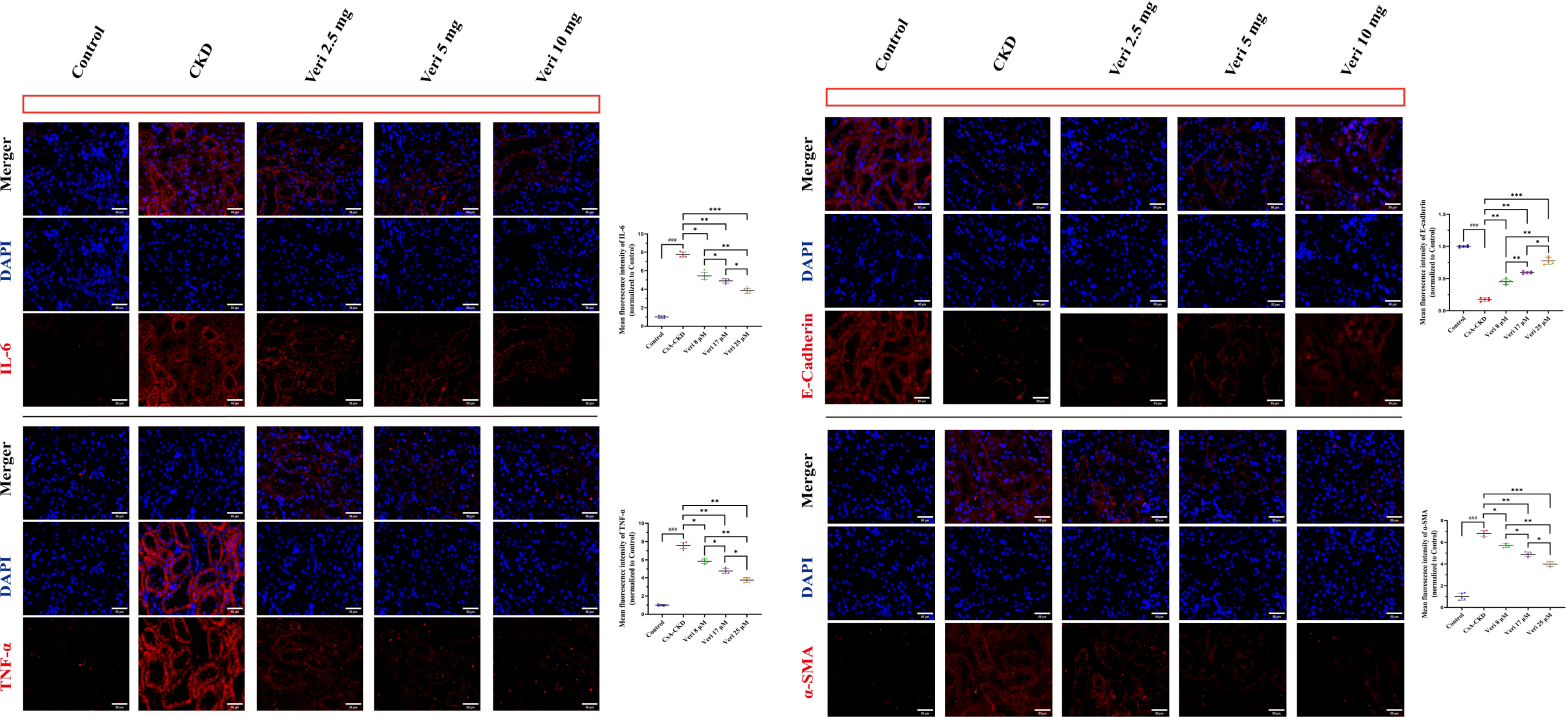
Immunofluorescence analysis of inflammatory and fibrotic protein expression in renal tissues (n = 5; scale bar, 50 μm). Representative immunofluorescence images and quantitative analysis of IL-6, TNF-α, α-SMA, and E-Cadherin expression in kidney sections. Statistical analysis: one-way ANOVA with Bonferroni *post-hoc* test was used for multiple group comparisons. Data are presented as mean ± SD; ^*^p < 0.05, ^**^p < 0.01, ^***^p < 0.001 vs. model group; ^*^p < 0.05, ^**^p < 0.01 vs. low-dose vericiguat group; ^#^p < 0.05, ^###^p < 0.001 vs. control group.

Immunofluorescence analysis (**Figure 7**) quantitatively confirmed the dose-dependent reduction in IL-6, TNF-α, and α-SMA fluorescence intensity and the restoration of E-Cadherin signal by vericiguat (p < 0.05 vs. model group; p < 0.05–0.001 for dose comparisons), reinforcing its potent therapeutic actions at the tissue level.

#### 3.2.9 Vericiguat modulates the NF-κB/TGF-β1 axis at the transcriptional level

**Figure 8:**
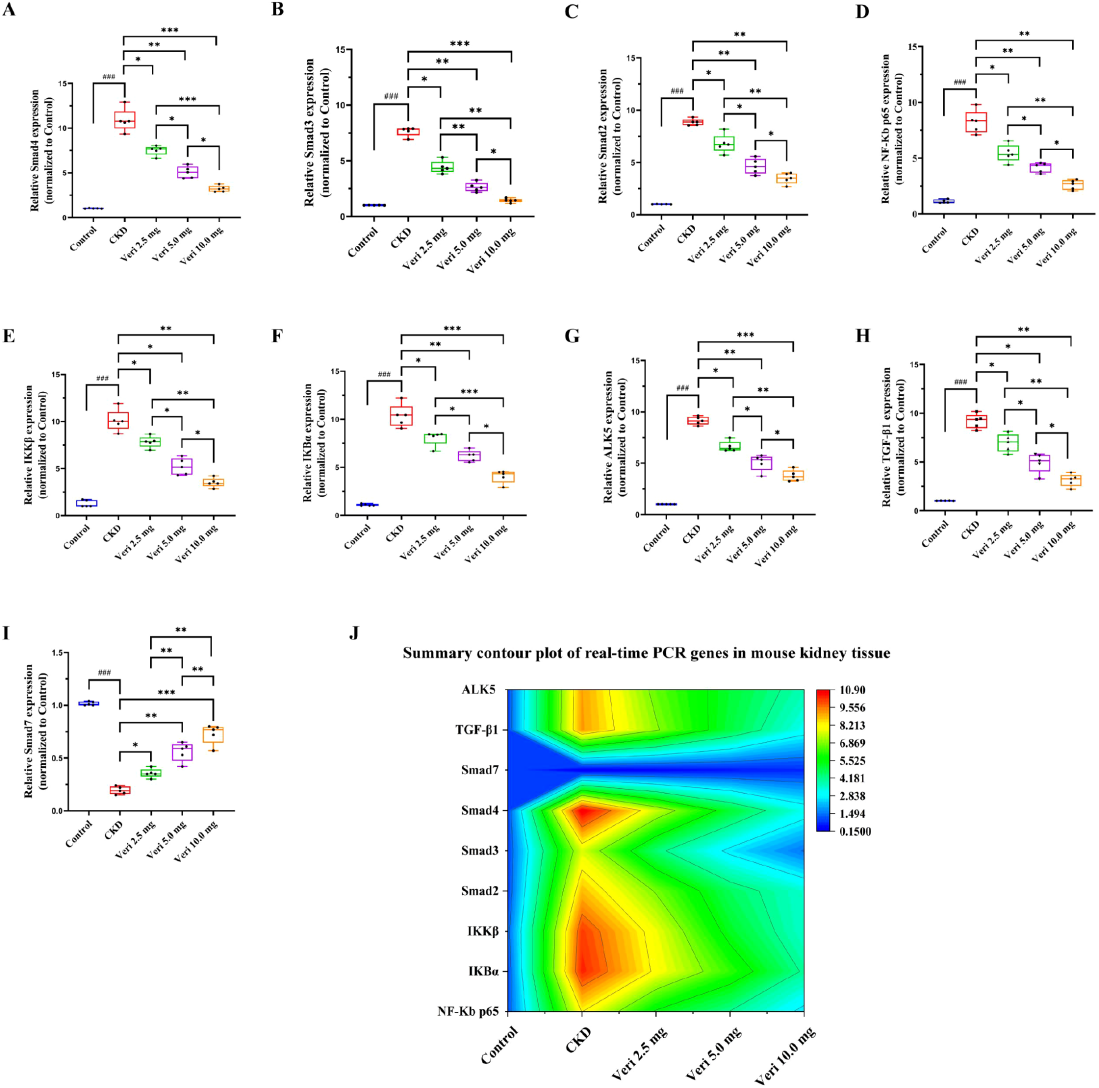
Vericiguat modulates mRNA expression of NF-κB/TGF-β1 axis-related genes in renal tissues (n = 5). **(A–I)** qRT-PCR analysis of mRNA levels of p65, IKBα, IKKβ, Smad2, Smad3, Smad4, TGF-β1, ALK5, and Smad7. **(J)** Ridge line plot summarizing the expression trends of NF-κB/TGF-β1 axis-related genes across groups. Statistical analysis: one-way ANOVA with Bonferroni *post-hoc* test was used for multiple group comparisons **(A–I)**. Data are presented as mean ± SD; ^*^p < 0.05, ^**^p < 0.01, ^***^p < 0.001 vs. model group; ^*^p < 0.05, ^**^p < 0.01 vs. low-dose vericiguat group; ^#^p < 0.05, ^###^p < 0.001 vs. control group.

qRT-PCR analysis (**Figures 8A–I**) demonstrated that CsA challenge significantly upregulated the mRNA expression of key NF-κB (p65, IKBα, and IKKβ) and TGF-β1/Smad (Smad2, Smad3, Smad4, TGF-β1, and ALK5) pathway components while downregulating inhibitory Smad7 (p < 0.001). Vericiguat treatment counteracted these changes in a dose-dependent manner (p < 0.05), effectively suppressing the pro-inflammatory and pro-fibrotic transcriptional program. A ridge line plot visually integrates the expression trends of all axis-related genes across groups (**Figure 8J**).

#### 3.2.10 Vericiguat inhibits NF-κB and TGF-β1/Smad signaling at the protein level

**Figure 9:**
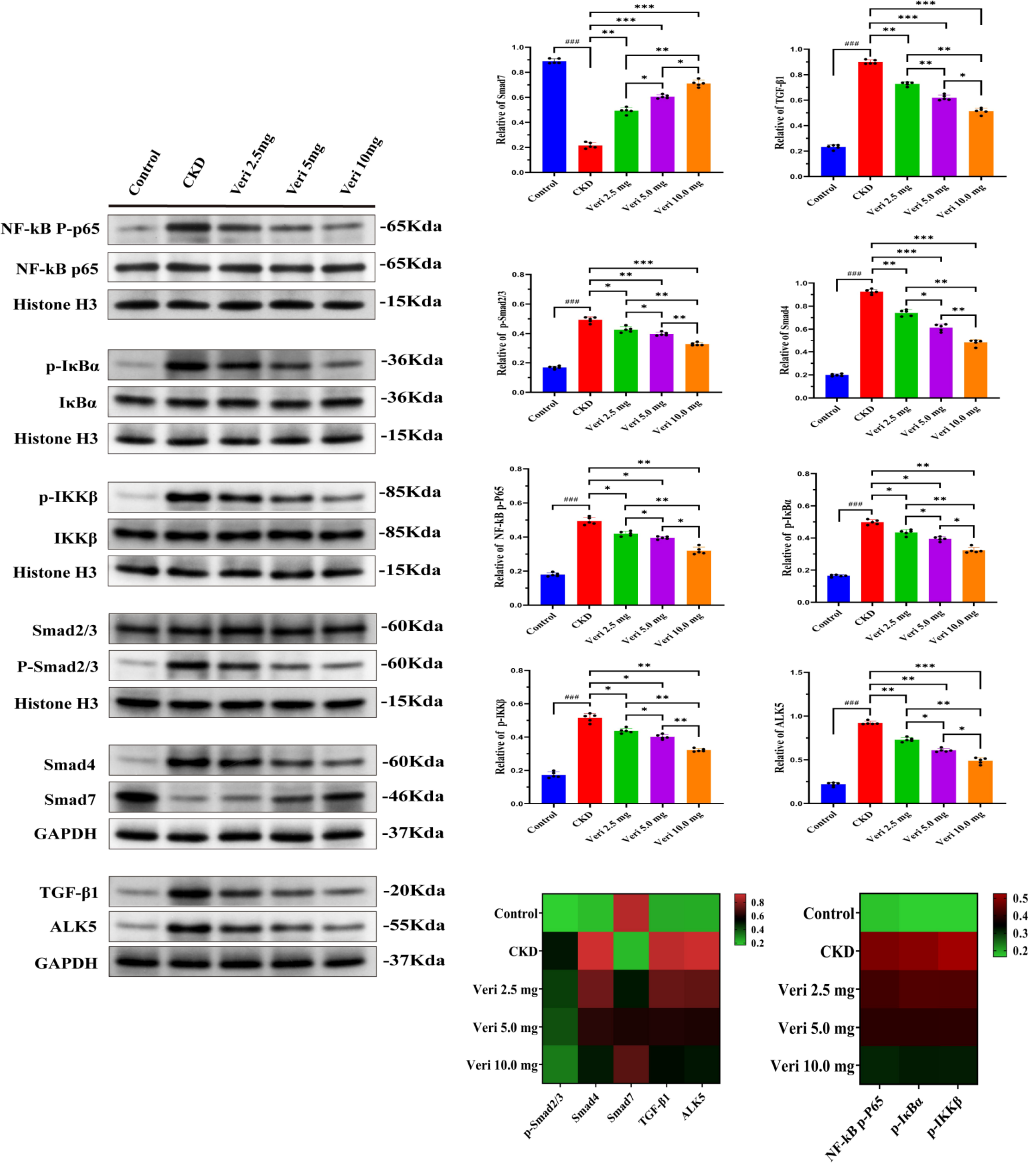
Vericiguat regulates protein expression of the NF-κB/TGF-β1 axis in renal tissues (n = 5). Western blotting analysis and quantitative densitometry of phosphorylated and total protein levels of NF-κB p65, IKBα, IKKβ, Smad2/3, Smad4, Smad7, TGF-β1, and ALK5. A heatmap summarizes the expression patterns of these proteins across experimental groups. Statistical analysis: one-way ANOVA with Bonferroni *post-hoc* test was used for multiple group comparisons. Data are presented as mean ± SD; ^*^p < 0.05, ^**^p < 0.01, ^***^p < 0.001 vs. model group; ^*^p < 0.05, ^**^p < 0.01 vs. low-dose vericiguat group; ^#^p < 0.05, ^###^p < 0.001 vs. control group.

Western blotting analysis (**Figure 9**) confirmed the activation of the NF-κB/TGF-β1 axis in model kidneys, evidenced by the increased phosphorylation of p65, IKBα, IKKβ, and Smad2/3; elevated levels of Smad4, TGF-β1, and ALK5; and decreased Smad7 (p < 0.001). Vericiguat treatment dose-dependently reversed these alterations (p < 0.05), demonstrating robust inhibition of both signaling pathways at the protein level. A corresponding heatmap provides a comprehensive view of the dose-responsive modulation of this core signaling network (**Figure 9**).

### 3.3 Cell-based functional and mechanistic validation

#### 3.3.1 Cytotoxicity and IC_50_ determination of vericiguat

CCK-8 assays demonstrated that vericiguat treatment for 24 or 48 h significantly reduced HK-2 cell viability at concentrations exceeding 20 µM (**Figures 2A–C**). Dose–response analysis yielded IC_50_ values of 24.08 µM at 24 h and 17.86 µM at 48 h. Notably, while the highest concentration tested (25 µM) exceeded the 48-h IC_50_ and reduced cell viability by >50%, it was deliberately included to establish a full concentration–response profile and to assess the maximal pharmacodynamic effect of vericiguat on the NF-κB/TGF-β1 axis. Importantly, the lower concentrations (8 and 17 µM) exhibited no significant cytotoxicity (cell viability >85% at 48 h) yet still exerted substantial anti-inflammatory, anti-fibrotic, and antioxidant activities. Based on these data, we selected three concentrations for subsequent mechanistic studies: 8 µM (low, sub-cytotoxic), 17 µM (medium, approximating the 48 h IC_50_), and 25 µM (high, supra-cytotoxic) to comprehensively characterize the therapeutic window and mechanism of action.

#### 3.3.2 Vericiguat attenuates CsA-induced oxidative stress

CsA stimulation markedly increased intracellular ROS levels in HK-2 cells (p < 0.001 vs. control). Vericiguat co-treatment significantly reduced ROS in a concentration-dependent manner (p < 0.05 vs. CsA alone), with the high dose showing superior efficacy to the medium and low doses (p < 0.05; **Figure 2G**).

#### 3.3.3 Independent effects of vericiguat on cytotoxicity, ROS, inflammation, and fibrosis in HK-2 cells

To rigorously exclude confounding effects of cytotoxicity on downstream readouts, we assessed the standalone effects of vericiguat. ELISA analysis showed no marked changes in IL-6, TNF-α, α-SMA, or E-Cadherin expression upon vericiguat monotherapy (p > 0.05; **Supplementary Figures 3A–D**). Similarly, DCFH-DA staining revealed that vericiguat alone (8–25 μM) did not significantly alter intracellular ROS levels compared to the control group (**Supplementary Figure 3F**). CCK-8 assays indicated that cell viability remained above 50% in the low- and medium-dose groups (8 and 17 μM), whereas viability fell below 50% in the high-dose group (25 μM), suggesting that higher concentrations of vericiguat may exert cytotoxic effects on HK-2 cells (**Supplementary Figure 3E**). These results confirm that the anti-inflammatory and anti-fibrotic effects observed in the CsA + vericiguat groups are attributable to the specific modulation of the CsA-activated NF-κB/TGF-β1 axis rather than non-specific cytotoxicity. Moreover, the robust protective effects achieved at the non-cytotoxic low and medium doses (8 and 17 μM) highlight a therapeutically relevant concentration range that effectively disrupts pathological signaling without compromising cell viability.

#### 3.3.4 Suppression of inflammatory and fibrotic markers by vericiguat

**Figure 10:**
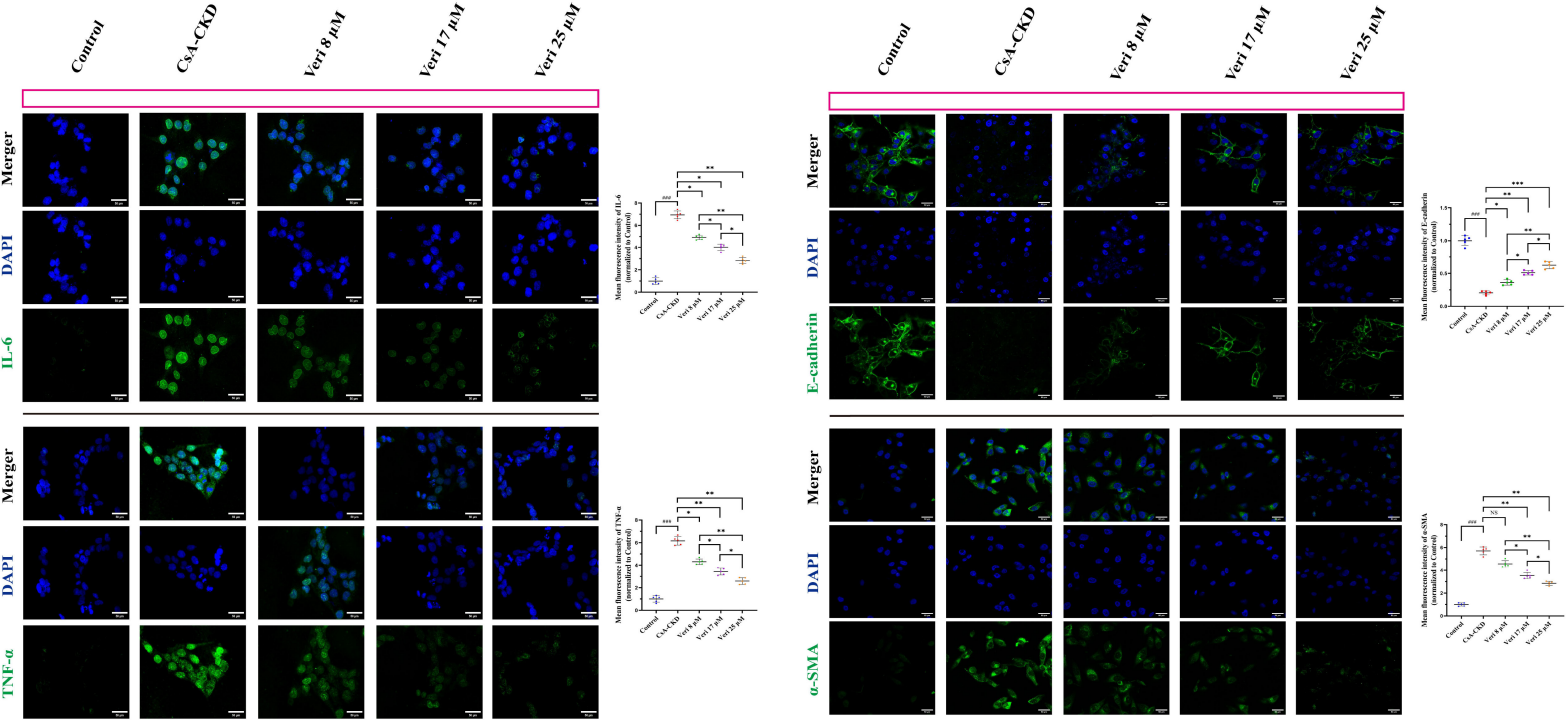
Vericiguat reduces inflammatory and fibrotic protein expression in cyclosporine in (CsA)-stimulated HK-2 cells (n = 5; scale bar, 50 μm). Representative immunofluorescence images and quantitative analysis of IL-6, TNF-α, α-SMA, and E-Cadherin expression in HK-2 cells treated with CsA and varying doses of vericiguat. Statistical analysis: one-way ANOVA with Bonferroni *post-hoc* test was used for multiple group comparisons. Data are presented as mean ± SD; ^*^p < 0.05, ^**^p < 0.01, ^***^p < 0.001 vs. CsA group; ^*^p < 0.05, ^**^p < 0.01 vs. low-dose vericiguat group; ^#^p < 0.05, ^###^p < 0.001 vs. control group.

Immunofluorescence analysis confirmed that CsA robustly upregulated IL-6, TNF-α, and α-SMA while downregulating E-Cadherin (p < 0.001 vs. control; **Figure 10**). Vericiguat dose-dependently reversed these effects, significantly reducing IL-6 and TNF-α (all doses, p < 0.05) and restoring E-Cadherin. The anti-fibrotic effect on α-SMA was significant only at medium and high doses (p < 0.05 vs. low dose), with the high dose being the most effective (p < 0.05 vs. medium dose). These findings align with the *in vivo* data, confirming dose-dependent anti-inflammatory and anti-fibrotic actions.

#### 3.3.5 Vericiguat inhibits the NF-κB/TGF-β1 axis in HK-2 cells

**Figure 11:**
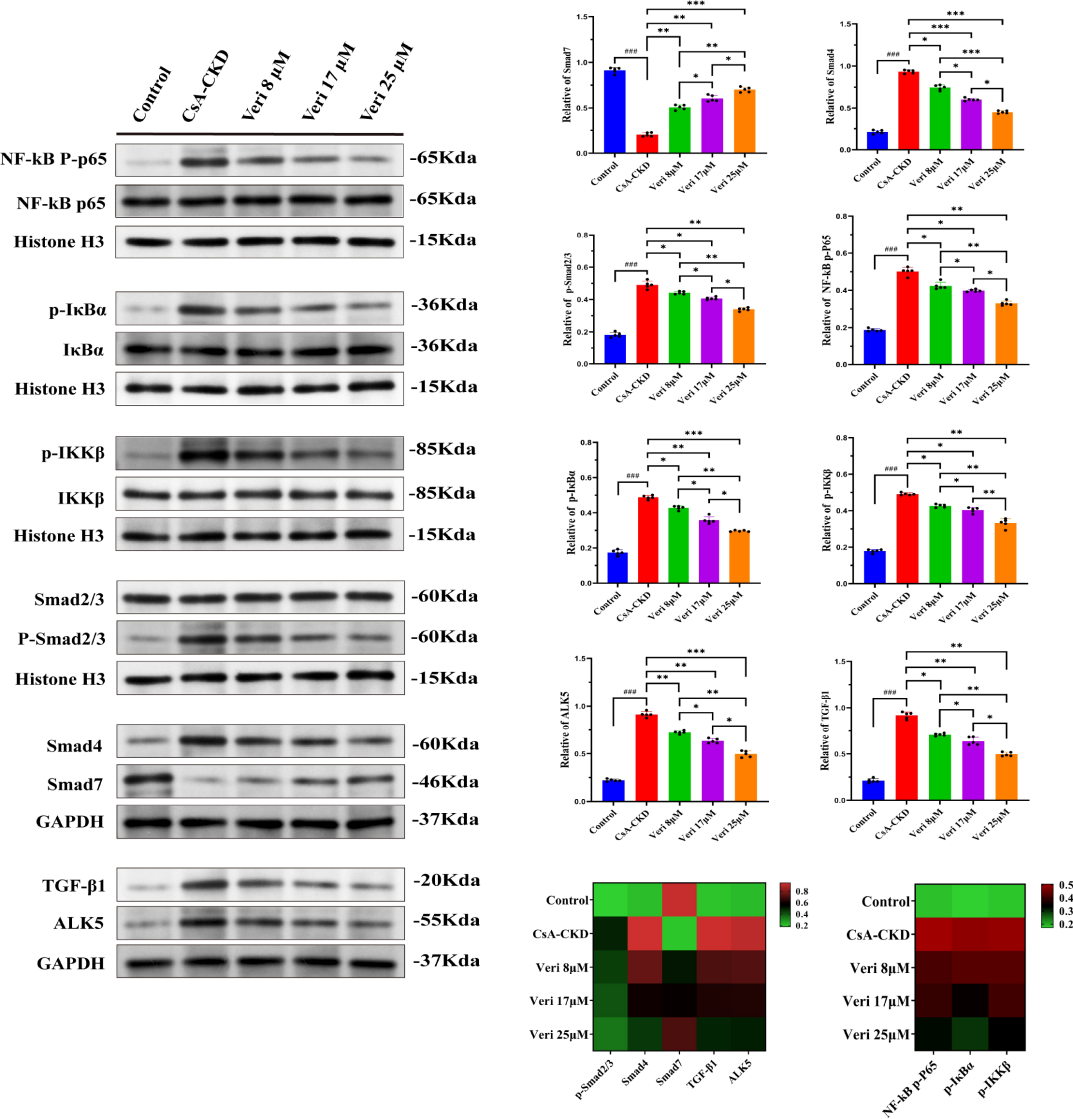
Vericiguat modulates the NF-κB/TGF-β1 axis in cyclosporine A (CsA)-induced HK-2 cells (n = 5). Western blotting analysis and quantitative densitometry of phosphorylated and total protein levels of NF-κB p65, IKBα, IKKβ, Smad2/3, Smad4, Smad7, TGF-β1, and ALK5 in HK-2 cells. A heatmap summarizes the expression patterns of these proteins across experimental groups. Statistical analysis: one-way ANOVA with Bonferroni *post-hoc* test was used for multiple group comparisons. Data are presented as mean ± SD; ^*^p < 0.05, ^**^p < 0.01, ^***^p < 0.001 vs. CsA group; ^*^p < 0.05, ^**^p < 0.01 vs. low-dose vericiguat group; ^#^p < 0.05, ^###^p < 0.001 vs. control group.

Western blotting analysis demonstrated that CsA activated the NF-κB pathway (increased p-IKKβ, p-IκBα, and p-p65) and the TGF-β1/Smad pathway (increased TGF-β1, ALK5, p-Smad2/3, and Smad4 and decreased Smad7) (p < 0.001 vs. control; **Figure 11**). Vericiguat treatment dose-dependently suppressed the phosphorylation of IKKβ, IκBα, p65, and Smad2/3; reduced Smad4 and TGF-β1/ALK5 levels; and restored Smad7 expression (p < 0.05 vs. CsA; greater effect with medium/high doses vs. low dose, p < 0.05). A heatmap visualization consolidated these dose-dependent inhibitory effects on the NF-κB/TGF-β1 axis.

#### 3.3.6 Validation of p65-knockdown efficiency

qRT-PCR confirmed highly efficient knockdown of p65 in HK-2 cells stably transduced with shRNA-p65 lentivirus compared to the shRNA-NC control (p < 0.001; **Figure 2D**).

#### 3.3.7 p65 knockdown potentiates Vericiguat’s effects

**Figure 12:**
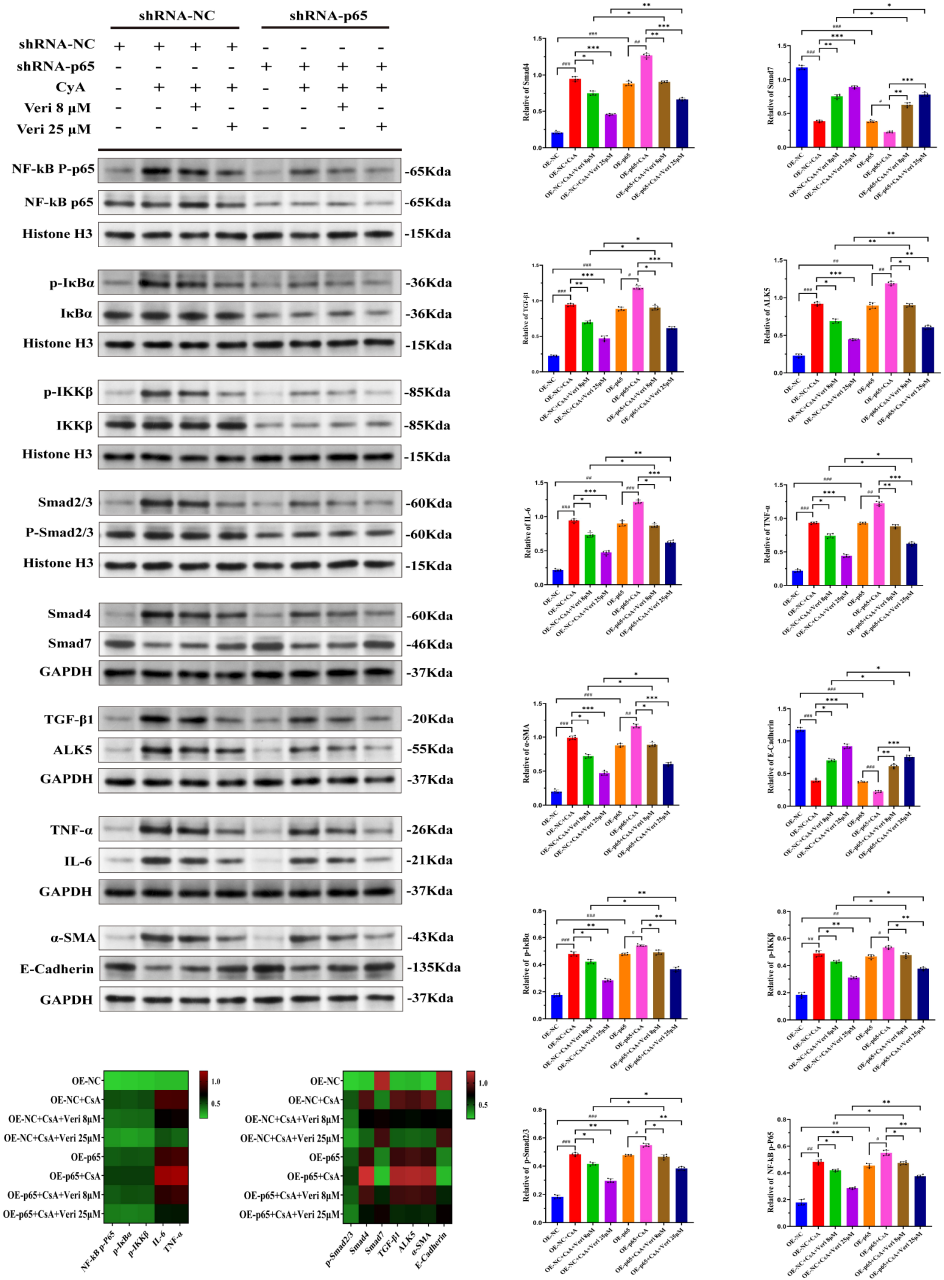
Knockdown of p65 attenuates cyclosporine A (CsA)-induced activation of the NF-κB/TGF-β1 axis and enhances the anti-inflammatory and anti-fibrotic effects of vericiguat (n = 5). Western blotting analysis of key proteins in the NF-κB/TGF-β1 axis and related inflammatory/fibrotic markers in HK-2 cells with stable p65 knockdown (shRNA-p65) under CsA and vericiguat treatment. A heatmap summarizes protein expression across groups. Statistical analysis: one-way ANOVA with Bonferroni *post-hoc* test was used for multiple group comparisons. Data are presented as mean ± SD; ^*^p < 0.05, ^**^p < 0.01, ^***^p < 0.001 vs. shRNA-NC + CsA/ShRNA-p65 + CsA groups; ^*^p < 0.05, ^**^p < 0.01 vs. shRNA-NC + CsA/ShRNA-p65 + CsA low-dose vericiguat group; ^#^p < 0.05, ^###^p < 0.001 vs. shRNA-NC/ShRNA-p65 group.

Stable p65 knockdown alone reduced the activation of the NF-κB/TGF-β1 axis and downstream inflammatory/fibrotic effectors (p < 0.05 vs. shRNA-NC; **Figure 12**). As expected, CsA treatment induced a robust activation of this axis in control cells (shRNA-NC + CsA). Notably, CsA-induced pathway activation was markedly blunted in p65-knockdown cells (shRNA-p65 + CsA). Vericiguat’s ability to further inhibit this axis and mitigate the CsA-induced protein changes was significantly enhanced in the p65-knockdown background compared to the control background, and this enhanced effect was dose-dependent. These results genetically validate p65 as a critical target node through which vericiguat exerts its effects.

#### 3.3.8 p65 overexpression partially rescues vericiguat’s protection

**Figure 13:**
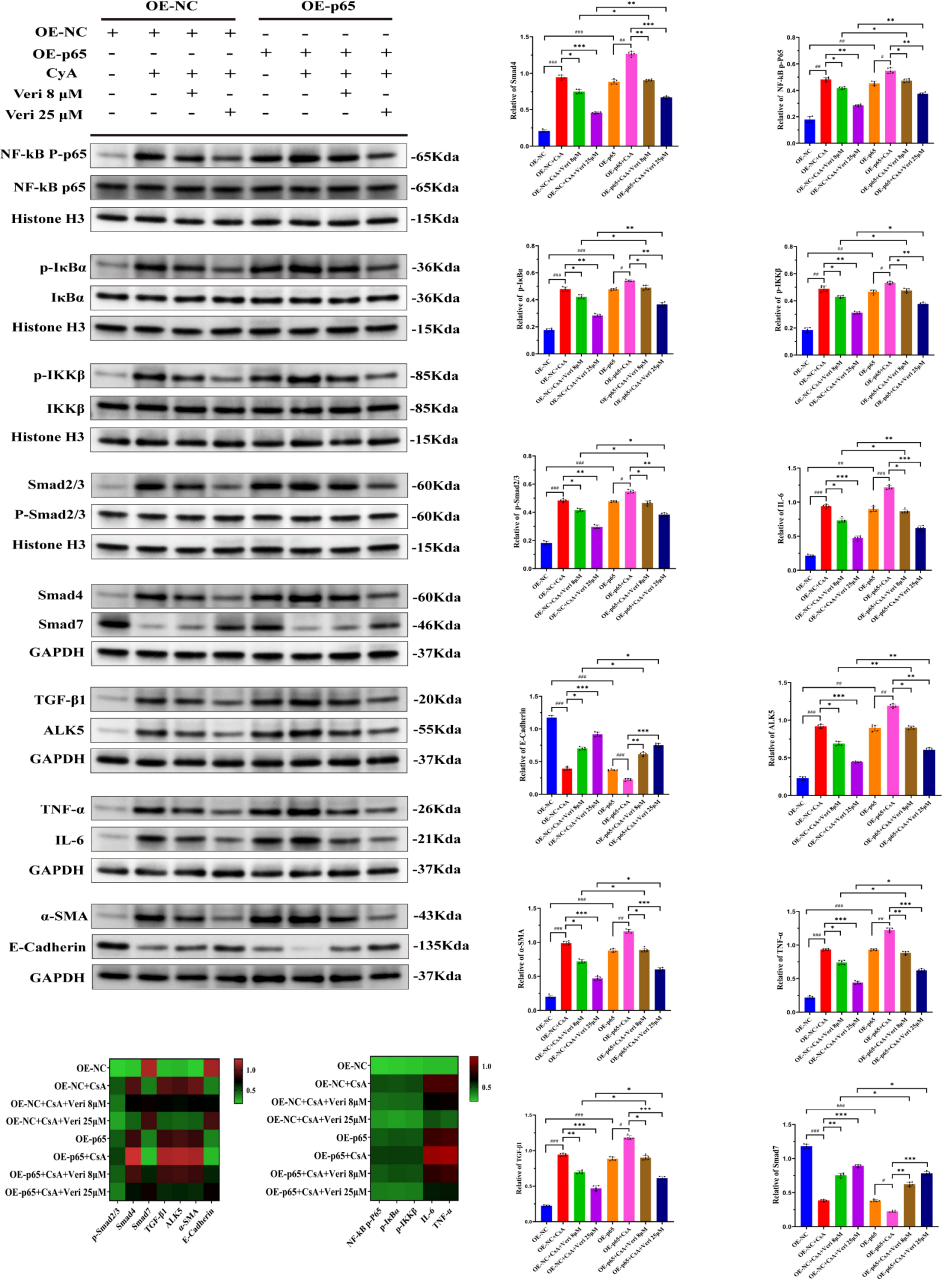
Overexpression of p65 exacerbates cyclosporine A (CsA)-induced activation of the NF-κB/TGF-β1 axis and partially reverses the protective effects of vericiguat (n = 5). Western blotting analysis of NF-κB/TGF-β1 axis proteins and inflammatory/fibrotic markers in HK-2 cells with stable p65 overexpression (OE-p65) under CsA and vericiguat treatment. A heatmap summarizes expression patterns across groups. Statistical analysis: one-way ANOVA with Bonferroni *post-hoc* test was used for multiple group comparisons. Data are presented as mean ± SD; ^*^p < 0.05, ^**^p < 0.01, ^***^p < 0.001 vs. OE-NC + CsA/OE-p65 + CsA groups; ^*^p < 0.05, ^**^p < 0.01 vs. OE-NC + CsA/OE-p65 + CsA low-dose vericiguat group; ^#^p < 0.05, ^###^p < 0.001 vs. OE-NC/OE-p65 group.

Conversely, p65 overexpression (OE-p65) exacerbated the CsA-induced activation of the NF-κB/TGF-β1 axis and the expression of inflammatory/fibrotic markers (p < 0.01 vs. OE-NC; **Figure 13**). While vericiguat still demonstrated efficacy in OE-p65 cells, its protective effects—including the suppression of pathway activation and the reversal of marker expression—were significantly attenuated compared to its effects in control OE-NC cells (p < 0.05). This attenuation was observed across vericiguat doses. Together with the knockdown data, these complementary gain-of-function experiments solidify the central role of p65 in mediating the renoprotective mechanism of vericiguat (**Figure 13**).

## 4 Discussion and outlook

CKD represents a growing global health burden, projected to become the fifth leading cause of mortality by 2040, driven largely by aging populations and the rising prevalence of diabetes and hypertension (33). Molecularly, CKD progression is fueled by a self-perpetuating cycle of inflammation, fibrosis, and oxidative stress (34). Renal tubular epithelial cells are now recognized as active contributors to disease pathogenesis, beyond being passive injury targets—a concept central to the “tubulocentric” hypothesis (35, 36). Under persistent insults such as drug toxicity, these cells undergo cell cycle arrest, senescence, mitochondrial dysfunction, and partial epithelial–mesenchymal transition (EMT). They subsequently secrete pro-fibrotic mediators like TGF-β1 and Connective Tissue Growth Factor (CTGF), fostering a pro-inflammatory microenvironment that activates fibroblasts and promotes excessive ECM deposition, ultimately culminating in renal interstitial fibrosis—a final common pathway in CKD progression (37–39).

CsA-induced nephropathy exemplifies such injury, recapitulating key features of human CKD. CsA impairs mitochondrial function, inducing permeability transition pore opening, electron transport chain failure, ROS overproduction, and ATP depletion. These events trigger endoplasmic reticulum stress and DNA damage and promote apoptosis and cellular senescence (40). CsA also potentiates TGF-β1 signaling and synergizes with the calcineurin–NFAT and NF-κB pathways to amplify fibrosis and inflammation (41, 42). Thus, the CsA model faithfully mirrors the transition from mitochondrial damage to inflammatory–fibrotic escalation, providing a robust platform for investigating interventions.

**Figure 14:**
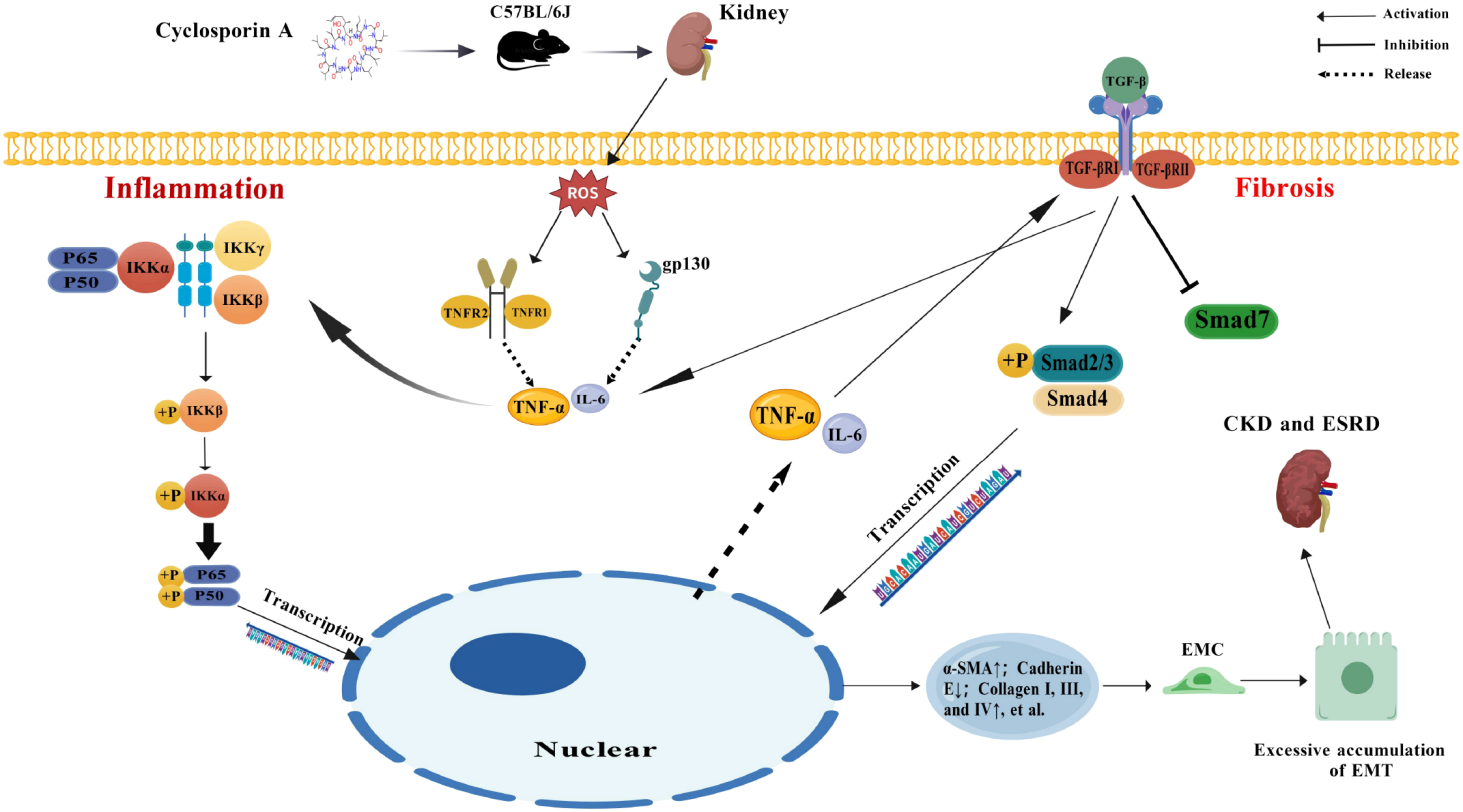
Proposed molecular mechanism of cyclosporine A-induced renal injury and the protective role of vericiguat via the ROS/NF-κB/TGF-β1/Smad signaling axis. Cyclosporine A (CsA) enters renal tubular epithelial cells and induces mitochondrial dysfunction, leading to excessive reactive oxygen species (ROS) production. ROS activates the IKK complex, resulting in IκBα phosphorylation and degradation, followed by nuclear translocation of NF-κB p65 and transcription of pro-inflammatory cytokines (e.g., TNF-α and IL-6). Concurrently, ROS promotes TGF-β1 signaling through ALK5-mediated phosphorylation of Smad2/3, complex formation with Smad4, and suppression of Smad7. This drives epithelial–mesenchymal transition (EMT), extracellular matrix deposition, and renal fibrosis. Vericiguat, as an sGC stimulator, interferes with this vicious cycle by inhibiting both NF-κB activation and TGF-β1/Smad signaling, thereby exerting anti-inflammatory, anti-fibrotic, and antioxidant effects in CsA-induced chronic kidney disease (CKD). ROS, reactive oxygen species; IKK, IκB Kinase; IκBα, Inhibitor of Nuclear Factor Kappa B Alpha; NF-κB, Nuclear Factor Kappa B; TNF-α, Tumor Necrosis Factor Alpha; IL-6, Interleukin-6; TGF-β1, Transforming Growth Factor Beta 1; ALK5, Activin Receptor-Like Kinase 5; Smad, Mothers Against Decapentaplegic Homolog (from *Drosophila*); α-SMA, Alpha-Smooth Muscle Actin; EMT, epithelial–mesenchymal transition; ECM, extracellular matrix.

Recent mechanistic insights into CsA nephrotoxicity highlight mitochondrial dysfunction and ROS overproduction as initiating events (**Figure 14**). CsA uncouples mitochondrial oxidative phosphorylation in tubular cells, leading to ROS bursts (43). ROS then acts as a second messenger, activating the IKK complex, which phosphorylates and degrades IκBα, liberating NF-κB (typically p65/p50) for nuclear translocation and transcription of pro-inflammatory genes (e.g., TNF-α and IL-6) (44, 45). Concurrently, ROS enhances TGF-β1 signaling via ALK5 receptor activation, leading to Smad2/3 phosphorylation, complex formation with Smad4, nuclear translocation, and transcriptional upregulation of fibrotic genes (e.g., α-SMA and collagen), alongside repression of inhibitory Smad7 (46). Critically, the NF-κB and TGF-β1 pathways engage in significant crosstalk: NF-κB upregulates TGF-β1 expression, while TGF-β1 reinforces NF-κB activity, creating a vicious cycle that perpetuates inflammation and fibrosis (47–49). This ROS/NF-κB/TGF-β1/Smad network drives tubular EMT, ECM accumulation, and interstitial fibrosis, culminating in end-stage renal disease.

Vericiguat, as a novel stimulator of sGC, enhances intracellular cGMP signaling, which is known to exert anti-inflammatory, anti-fibrotic, and antioxidant effects in various tissues. In the context of renal injury, cGMP/PKG signaling has been shown to inhibit NF-κB activation by suppressing IKKβ phosphorylation and IκBα degradation, thereby reducing the transcription of pro-inflammatory cytokines. Concurrently, cGMP signaling modulates TGF-β1/Smad pathway activity, potentially through Smad7 upregulation and Smad2/3 phosphorylation inhibition, leading to attenuated fibrotic responses. Our integrated analyses and experimental validation consistently support that vericiguat disrupts the ROS/NF-κB/TGF-β1/Smad axis, breaking the vicious cycle of inflammation and fibrosis in CsA-induced nephropathy.

In this study, we first identified 42 shared targets between vericiguat and CKD using network pharmacology. Topological analysis of protein–protein interaction networks pinpointed STAT3 and NFKB1 as core targets. Mendelian randomization provided genetic evidence supporting a causal role for NFKB1, STAT3, PIK3CG, and IKBKB in CKD risk, with NFKB1 and STAT3 emerging as significant risk factors. Molecular docking and dynamics simulations demonstrated high-affinity, stable binding of vericiguat to IKBKB and NFKB1, suggesting direct engagement with the NF-κB pathway. *In vivo*, vericiguat dose-dependently ameliorated CsA-induced renal dysfunction, reducing proteinuria, serum creatinine, and urea nitrogen, while attenuating histological damage, including tubular vacuolization, inflammatory infiltration, and collagen deposition. Vericiguat also suppressed the renal expression of IL-6, TNF-α, and α-SMA and restored E-Cadherin levels. Mechanistically, it inhibited the phosphorylation of IKKβ, IκBα, NF-κB p65, and Smad2/3, blunting both NF-κB and TGF-β1/Smad signaling. *In vitro*, vericiguat reduced ROS production, inflammatory and fibrotic protein expression, and pathway phosphorylation in CsA-stimulated HK-2 cells. Importantly, our cytotoxicity assessments confirmed that vericiguat alone did not compromise cell viability at the doses employed, thereby ensuring that the observed attenuation of ROS, inflammation, and fibrosis in CsA-treated cells reflects the genuine modulation of the NF-κB/TGF-β1 axis rather than artifacts stemming from cell loss. Our *in vitro* data delineate a clear therapeutic window for vericiguat: while the highest concentration (25 μM) elicited the strongest suppression of oxidative stress and inflammatory/fibrotic markers, it also reduced cell viability by >50% at 48 h. In contrast, the lower, non-cytotoxic concentrations (8 and 17 μM) still provided significant protection against CsA-induced injury, effectively attenuating ROS production, cytokine release, and fibrotic protein expression without impairing cellular integrity. This biphasic response underscores the importance of dose optimization for clinical translation. The low and medium doses likely represent a more physiologically and clinically meaningful therapeutic range, wherein vericiguat exerts its renoprotective effects primarily through pathway modulation rather than cytotoxicity. Future preclinical studies should focus on these subtoxic concentrations to better predict efficacious and safe dosing regimens in patients. Crucially, p65 knockdown augmented, while its overexpression attenuated, the protective effects of vericiguat, establishing p65 as a critical node in its mechanism of action and confirming the centrality of the NF-κB/TGF-β1 axis.

In conclusion, our integrative approach—combining computational prediction, genetic epidemiology, and experimental validation—establishes that vericiguat mitigates CsA-induced CKD by concurrently suppressing the NF-κB and TGF-β1 pathways, thereby exerting anti-inflammatory, anti-fibrotic, and renoprotective effects. These findings provide a compelling rationale for repurposing vericiguat for CKD. Future clinical trials should explore its potential, either as monotherapy or in combination with standard-care agents like RAAS inhibitors or SGLT2 inhibitors, to achieve multi-targeted control of CKD progression and improve patient outcomes (50).

## 5 Limitations

While this integrated study—combining network pharmacology, Mendelian randomization, and experimental validation—provides systematic evidence that vericiguat protects against CsA-induced CKD by targeting the NF-κB/TGF-β1 axis, several limitations should be acknowledged.

First, the clinical relevance of our findings warrants further validation. Our conclusions are drawn primarily from preclinical models, including CsA-induced mice and HK-2 cells. We lacked access to clinical cohorts or human kidney tissues to directly assess the expression of core targets like IKBKB and NFKB1, or to evaluate vericiguat’s efficacy in patients with CKD, particularly those with CsA-related nephrotoxicity. Given interspecies differences in drug metabolism and human disease heterogeneity, future prospective clinical studies are essential to confirm the translational potential of our results.

Second, due to practical constraints, the time-dependent effects of vericiguat were not fully characterized. Although 12 weeks of treatment in mice sufficed to demonstrate functional and histological improvements, it remains unclear whether earlier intervention or prolonged therapy could enhance efficacy or reverse established fibrosis. Similarly, fixed 48-h treatments in cell models may not capture slower adaptive responses. Studies with varied treatment initiation timepoints and extended durations are needed to identify the optimal therapeutic window.

Third, although the NF-κB/TGF-β1 axis emerged as a central mechanism, vericiguat likely acts through a broader network. Network pharmacology and MR implicated additional genes (e.g., PIK3CG and STAT3) whose contributions were not experimentally dissected. A more comprehensive mechanistic understanding could be achieved through CRISPR-based screening or multi-omics profiling.

Fourth, the CsA-induced nephrotoxicity model, while relevant for calcineurin inhibitor-related CKD, does not fully mirror the multifactorial pathogenesis of human CKD, which often includes diabetic and hypertensive nephropathy. Thus, the generalizability of our findings to other CKD subtypes requires validation. Moreover, the involvement of the sGC/cGMP pathway—vericiguat’s primary target—in modulating the NF-κB/TGF-β1 axis was inferred indirectly; genetic or pharmacological perturbation of sGC/cGMP signaling would provide more direct evidence.

Finally, we focused on vericiguat monotherapy. Its potential synergistic or additive effects with standard-of-care agents (e.g., RAAS inhibitors or SGLT2 inhibitors) remain unexplored and represent an important direction for future combination therapy studies.

## Data Availability

The original contributions presented in the study are included in the article/**Supplementary Material**. Further inquiries can be directed to the corresponding authors.
