# Supplementary material for: Vericiguat attenuates cyclosporine A-induced nephropathy by targeting the NF-κB/TGF-β1 axis: an integrated network pharmacology, Mendelian randomization, and experimental study
Source: Front Immunol. 2026 Jan 27;16:1756582. doi: 10.3389/fimmu.2025.1756582 (PMC12886507; doi:10.3389/fimmu.2025.1756582)

1. Animal kidney tissue protein

P-IκBα	IκBα	Histone H3
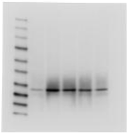	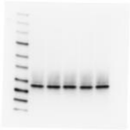	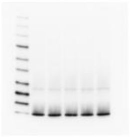
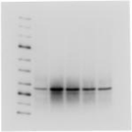	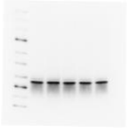	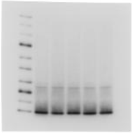
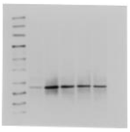	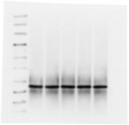	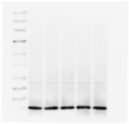
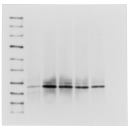	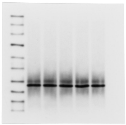	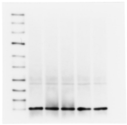
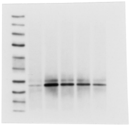	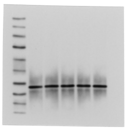	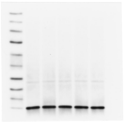
P-IKKβ 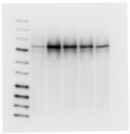	IKKβ 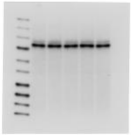	Histone H3 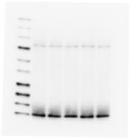

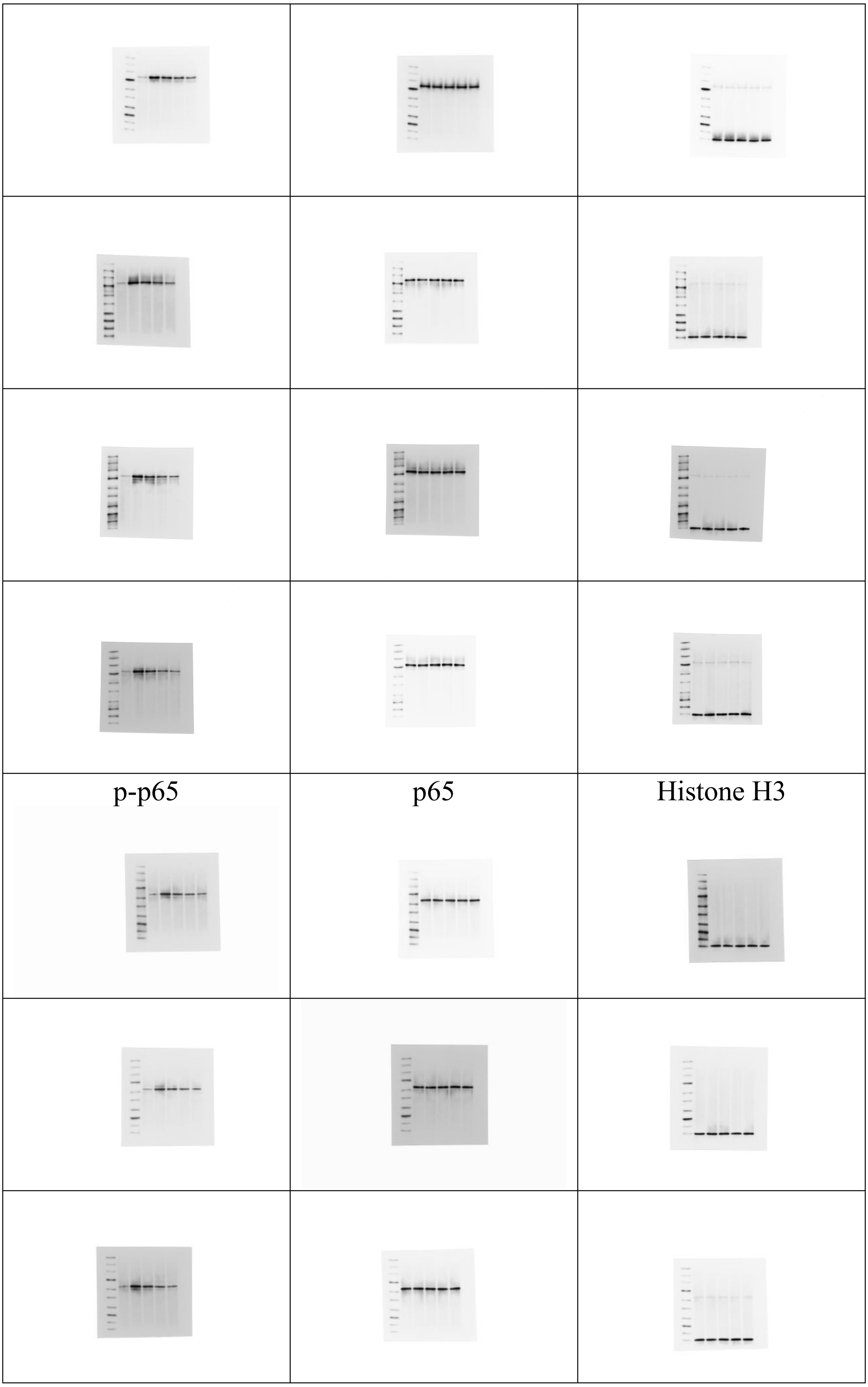

p-p65
p65
Histone H3

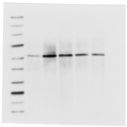	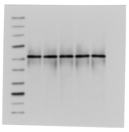	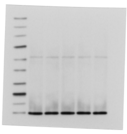
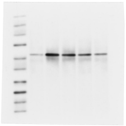	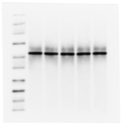	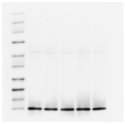
P-smad2/3<br>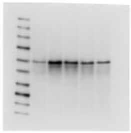	smad2/3<br>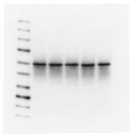	Histone H3<br>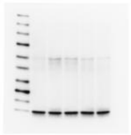
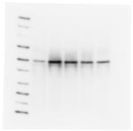	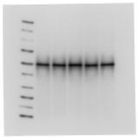	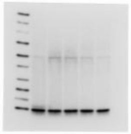
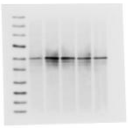	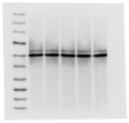	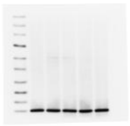
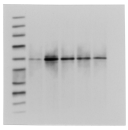	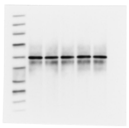	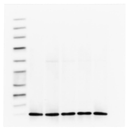
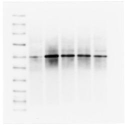	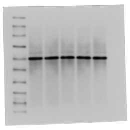	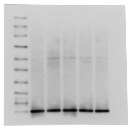

Smad4	Smad7	GAPDH
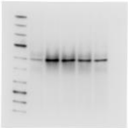	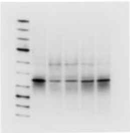	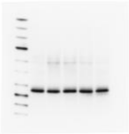
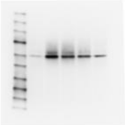	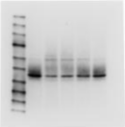	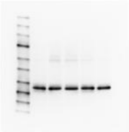
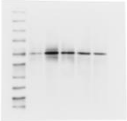	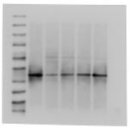	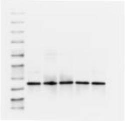
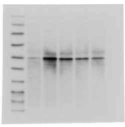	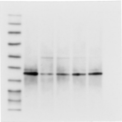	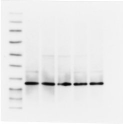
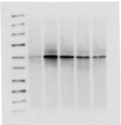	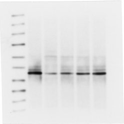	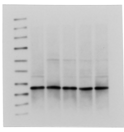
TGF-β1	ALK5	GAPDH
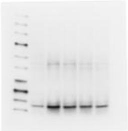	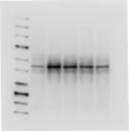	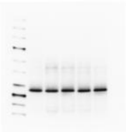
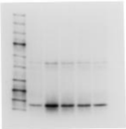	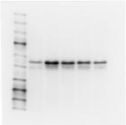	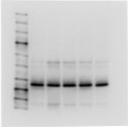

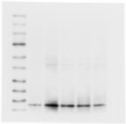	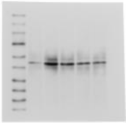	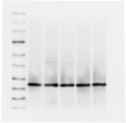
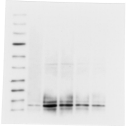	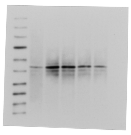	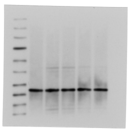
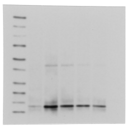	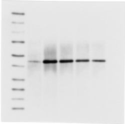	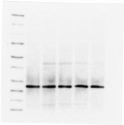

2. HK2 cell protein

P-IκBα	IκBα	Histone H3
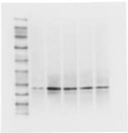	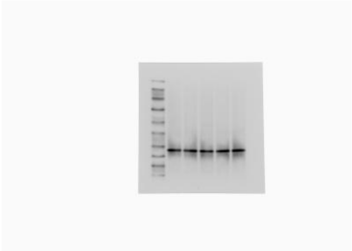	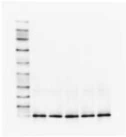
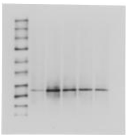	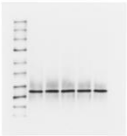	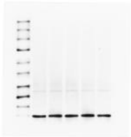
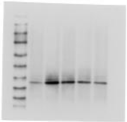	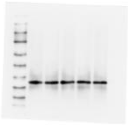	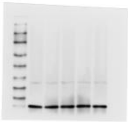

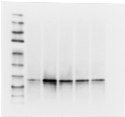	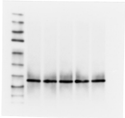	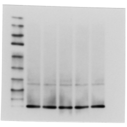
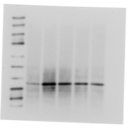	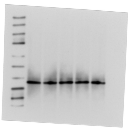	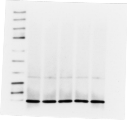
P-IKKβ<br>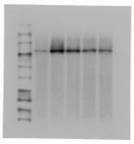	IKKβ<br>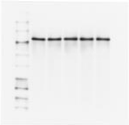	Histone H3<br>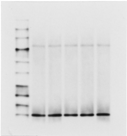
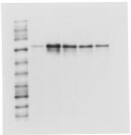	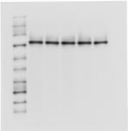	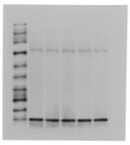
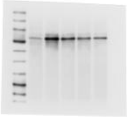	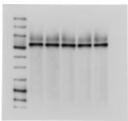	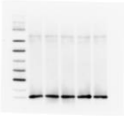
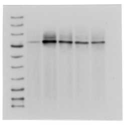	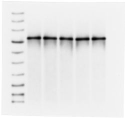	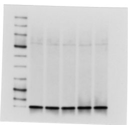
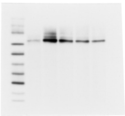	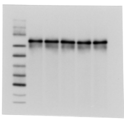	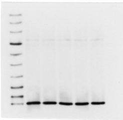

p-p65	p65	Histone H3
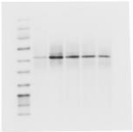	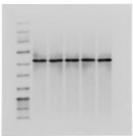	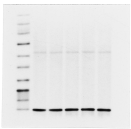
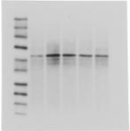	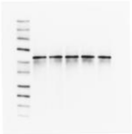	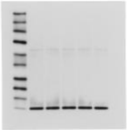
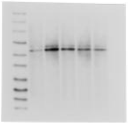	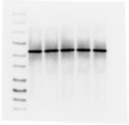	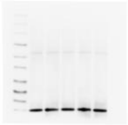
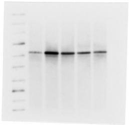	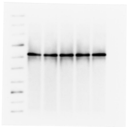	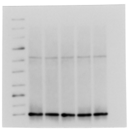
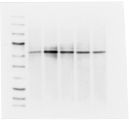	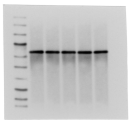	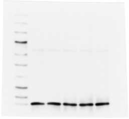
P-smad2/3	smad2/3	Histone H3
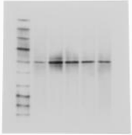	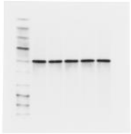	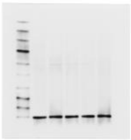
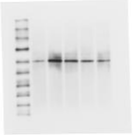	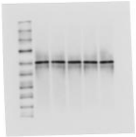	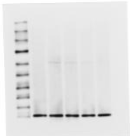

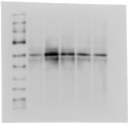	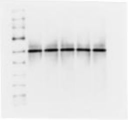	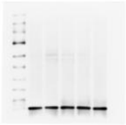
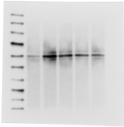	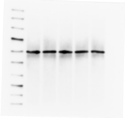	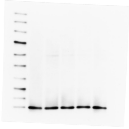
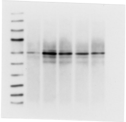	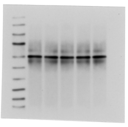	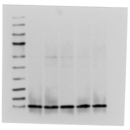
Smad4<br>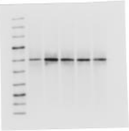	Smad7<br>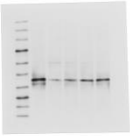	GAPDH<br>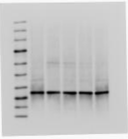
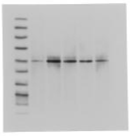	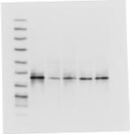	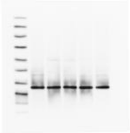
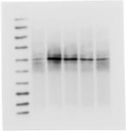	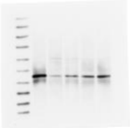	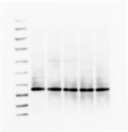
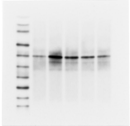	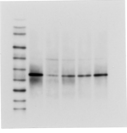	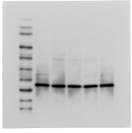

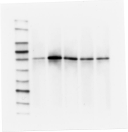	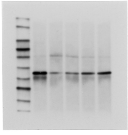	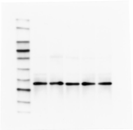
TGF-β1<br>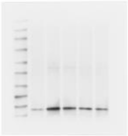	ALK5<br>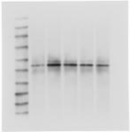	GAPDH<br>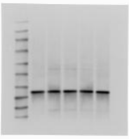
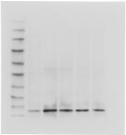	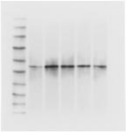	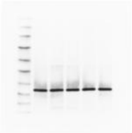
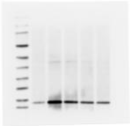	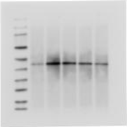	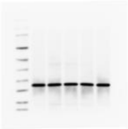
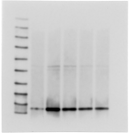	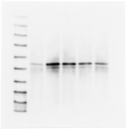	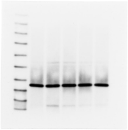
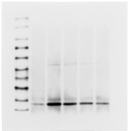	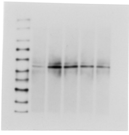	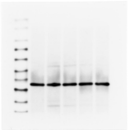

3. shRNA-p65 stably rotating HK2 protein

P-IκBα	IκBα	Histone H3

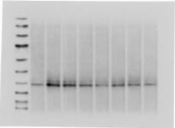	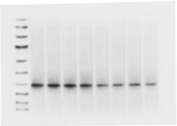	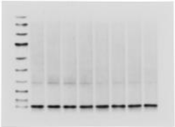
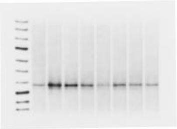	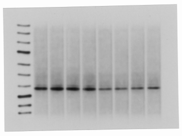	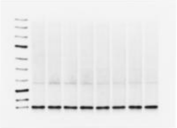
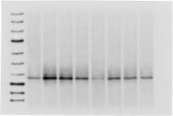	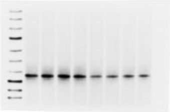	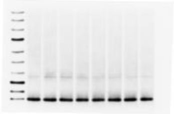
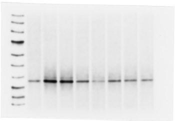	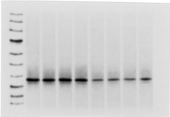	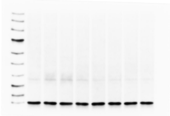
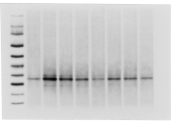	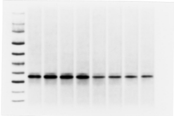	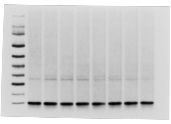
P-IKKβ 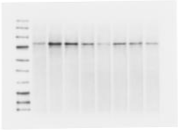	IKKβ 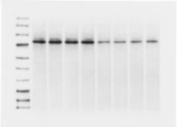	Histone H3 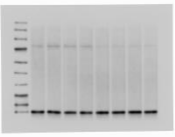
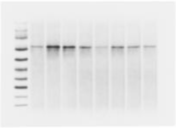	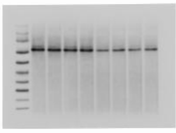	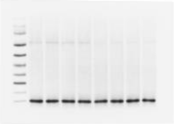

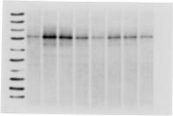	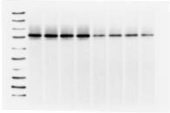	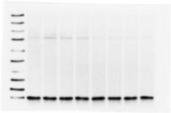
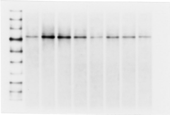	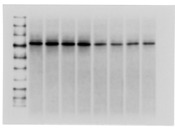	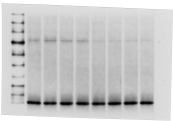
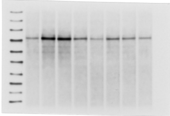	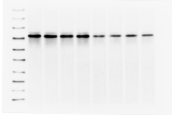	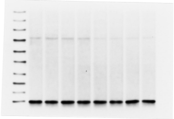
p-p65<br>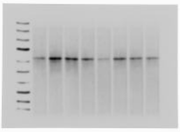	p65<br>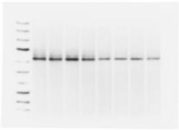	Histone H3<br>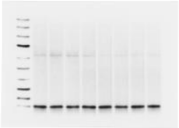
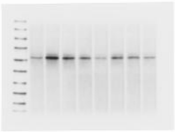	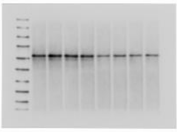	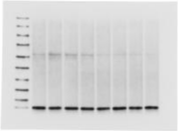
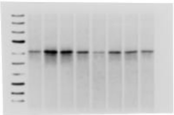	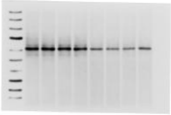	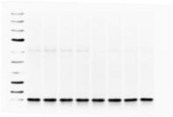
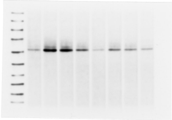	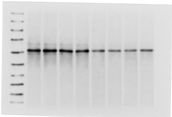	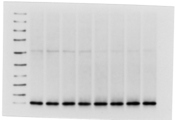

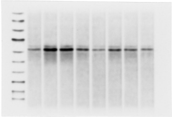	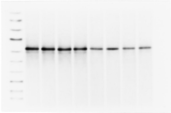	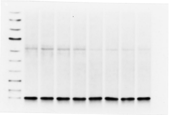
P-smad2/3<br>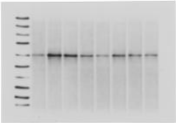	smad2/3<br>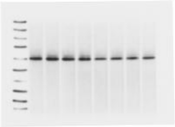	Histone H3<br>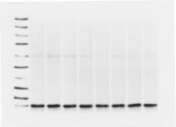
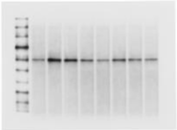	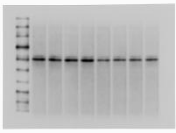	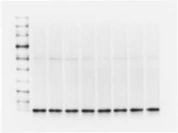
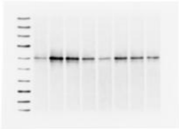	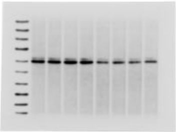	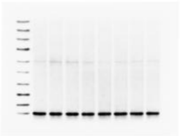
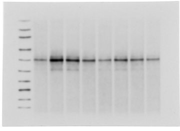	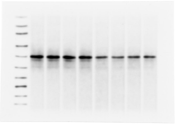	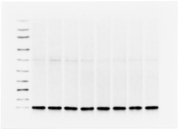
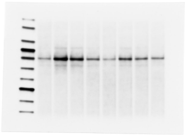	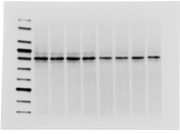	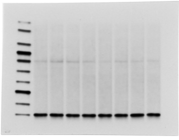
Smad4<br>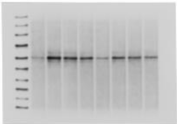	Smad7<br>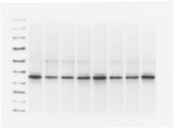	GAPDH<br>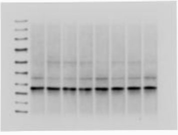

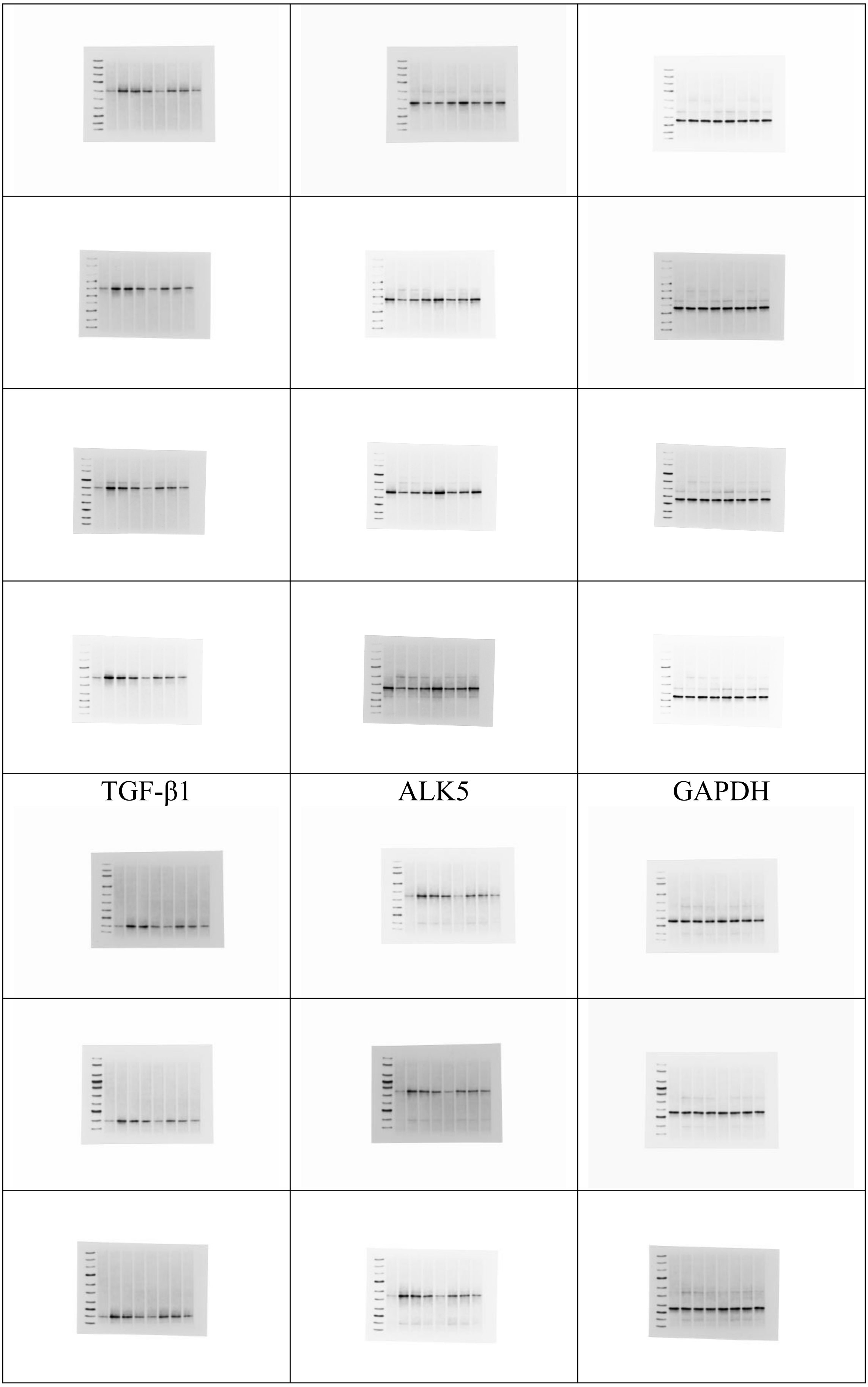
TGF-β1
ALK5
GAPDH

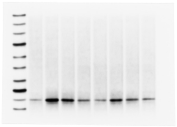	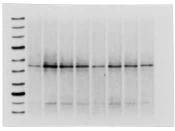	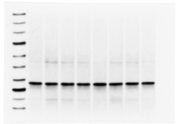
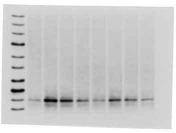	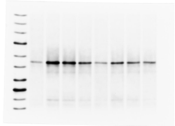	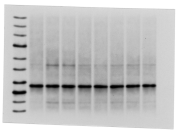
IL-6<br>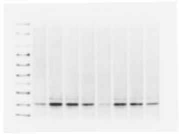	TNF-α<br>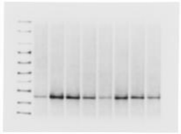	GAPDH<br>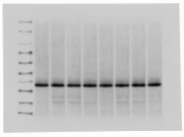
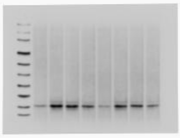	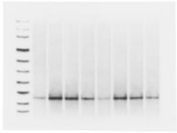	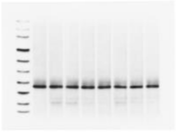
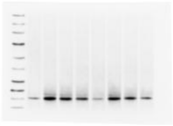	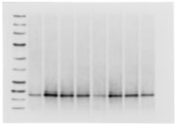	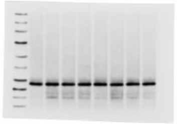
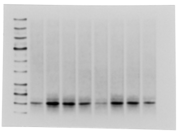	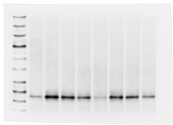	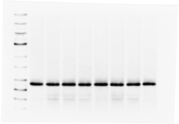
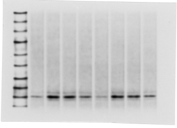	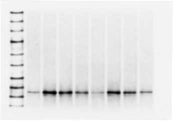	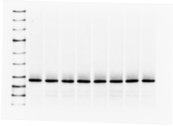

α-SMA	E-Cadherin	GAPDH
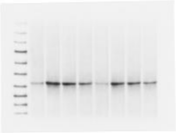	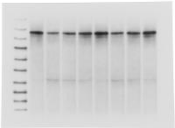	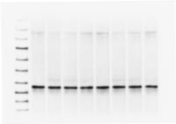
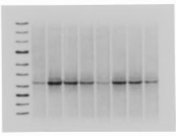	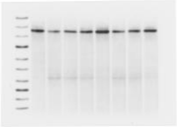	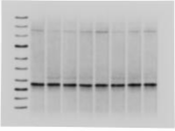
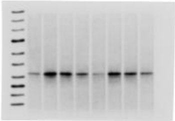	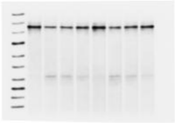	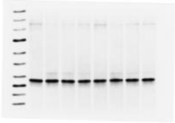
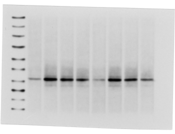	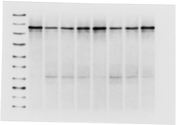	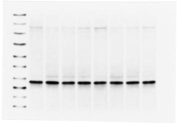
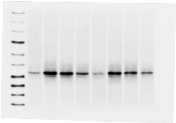	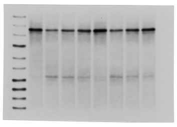	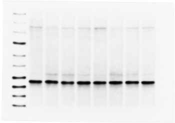

4. OE-p65 stably rotating HK2 protein

P-IκBα	IκBα	Histone H3
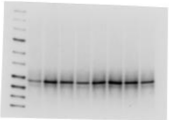	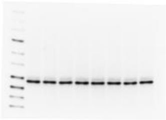	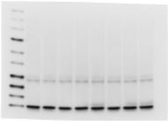

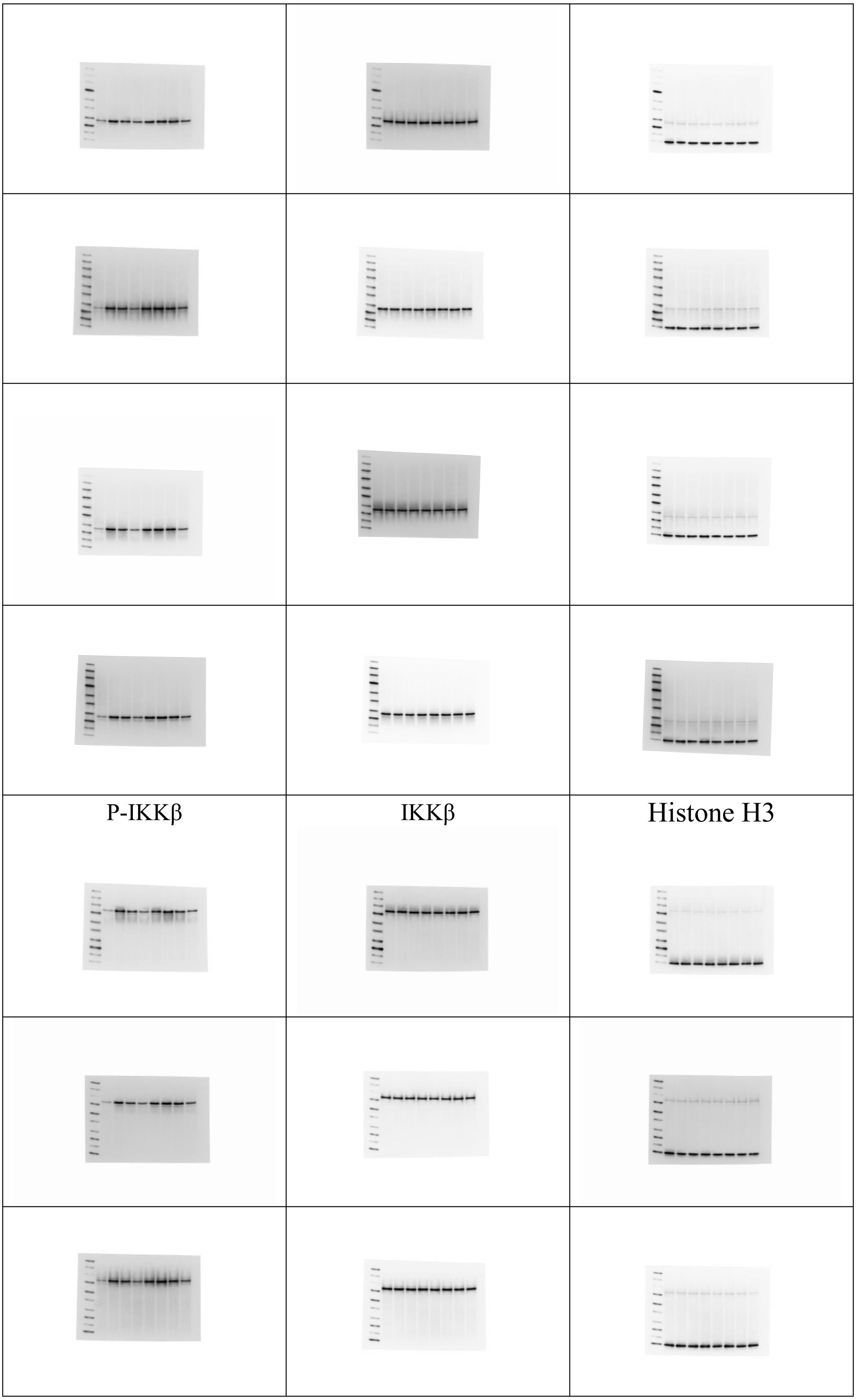
P-IKKβ
IKKβ
Histone H3

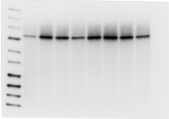	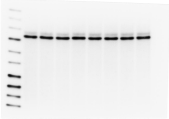	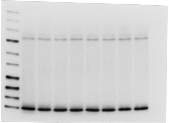
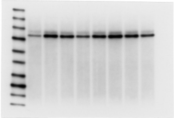	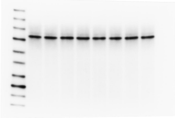	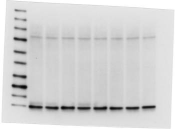
p-p65 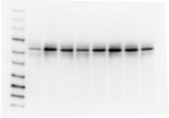	p65 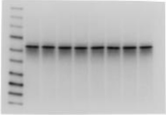	Histone H3 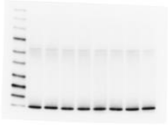
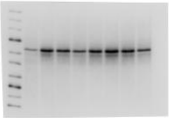	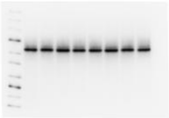	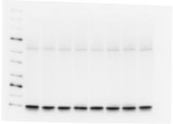
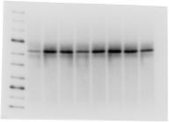	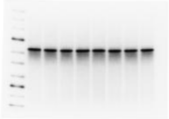	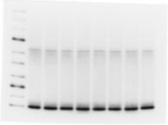
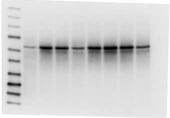	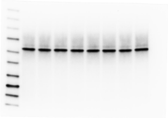	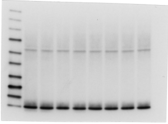
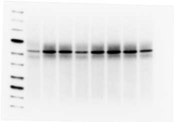	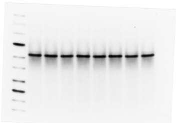	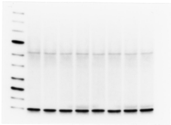

P-smad2/3	smad2/3	Histone H3
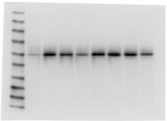	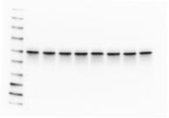	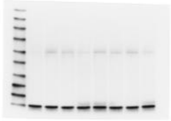
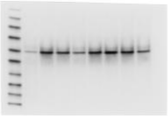	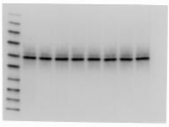	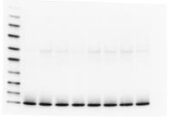
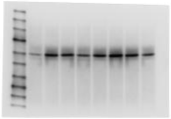	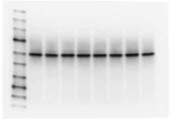	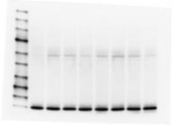
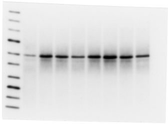	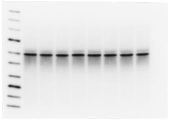	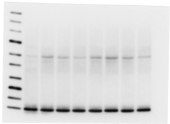
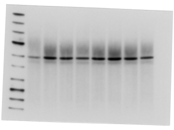	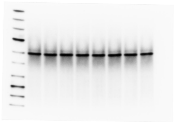	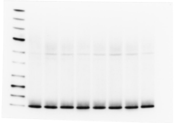
Smad4<br>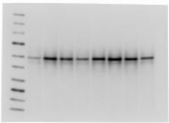	Smad7<br>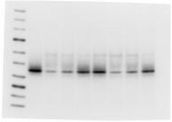	GAPDH<br>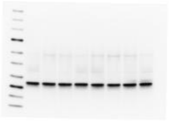
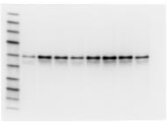	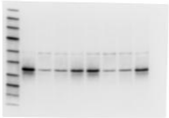	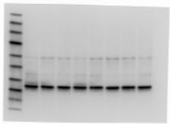

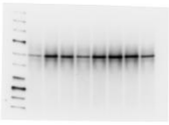	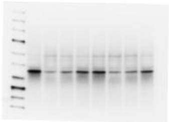	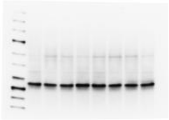
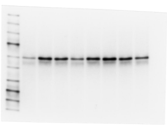	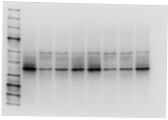	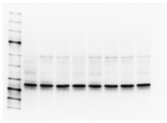
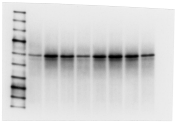	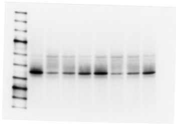	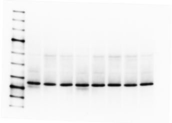
TGF-β1<br>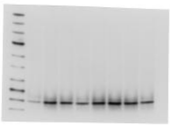	ALK5<br>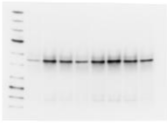	GAPDH<br>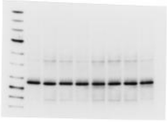
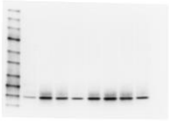	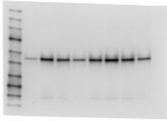	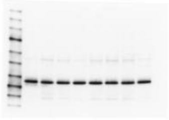
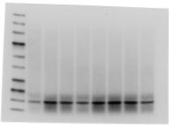	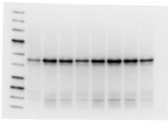	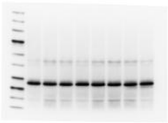
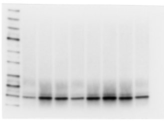	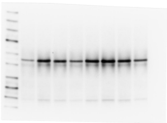	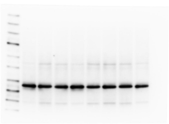

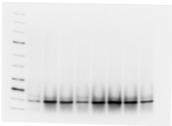	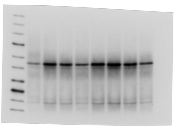	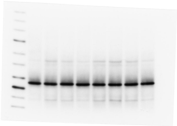
IL-6<br>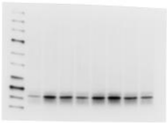	TNF-α<br>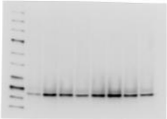	GAPDH<br>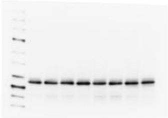
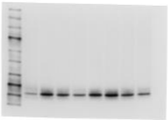	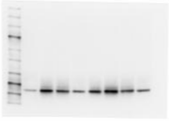	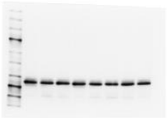
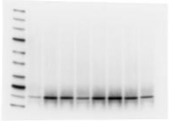	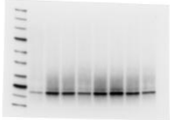	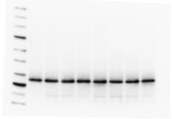
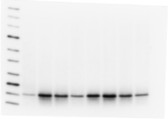	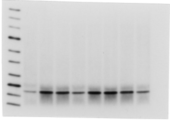	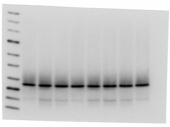
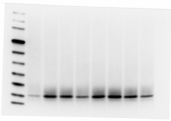	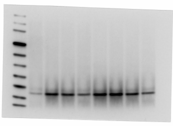	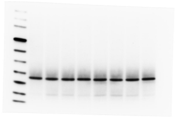
α-SMA<br>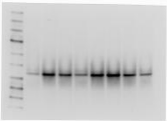	F-Cadherin<br>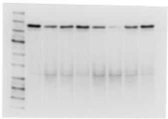	GAPDH<br>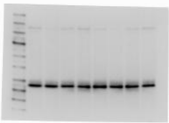

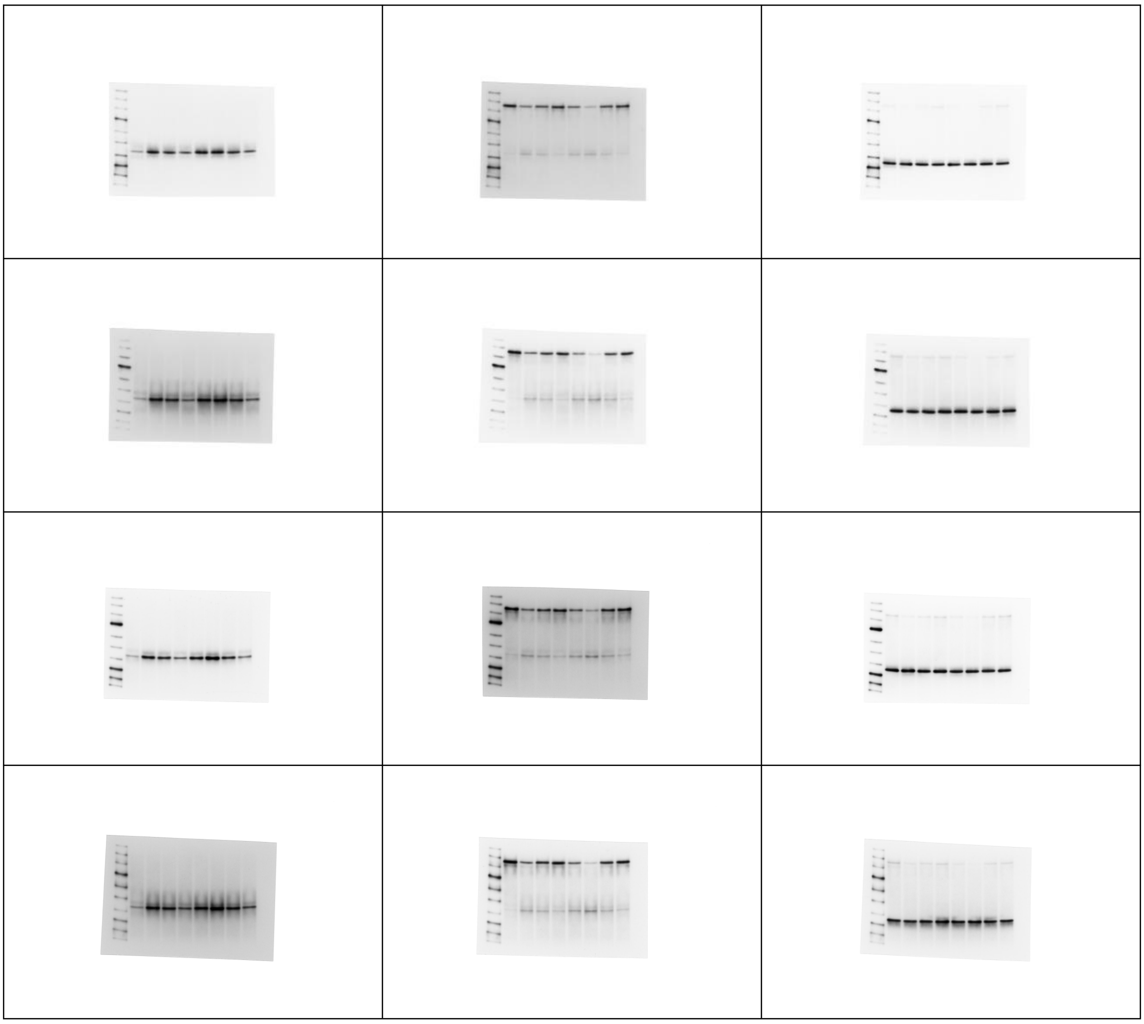

Supplement: Supplementary file 1 [file DataSheet1.pdf]
