# Supplementary material for: Vericiguat attenuates cyclosporine A-induced nephropathy by targeting the NF-κB/TGF-β1 axis: an integrated network pharmacology, Mendelian randomization, and experimental study
Source: Front Immunol. 2026 Jan 27;16:1756582. doi: 10.3389/fimmu.2025.1756582 (PMC12886507; doi:10.3389/fimmu.2025.1756582)
Supplement: Supplementary file 2 [file DataSheet2.pdf]

1. Animal kidney tissue protein

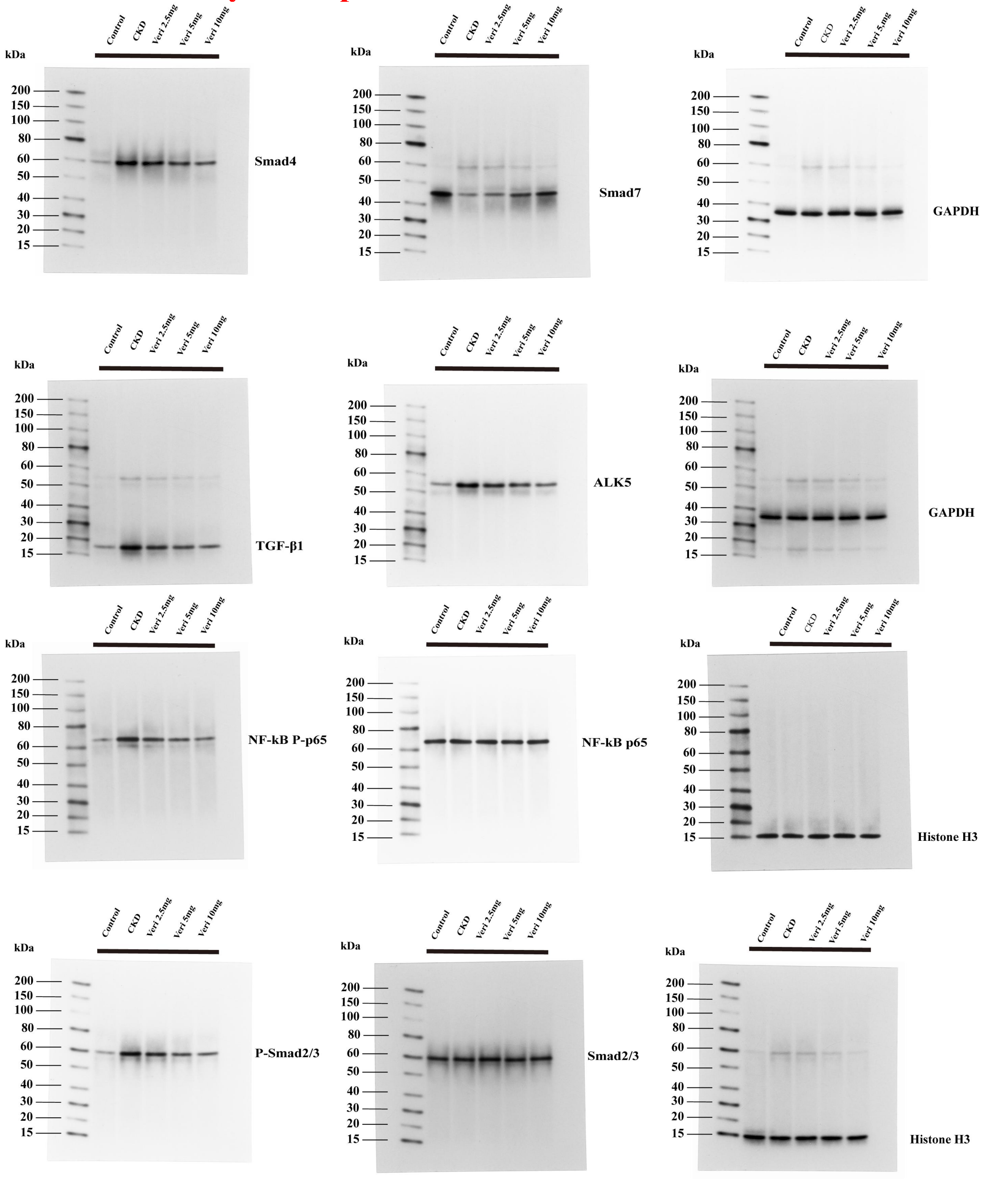

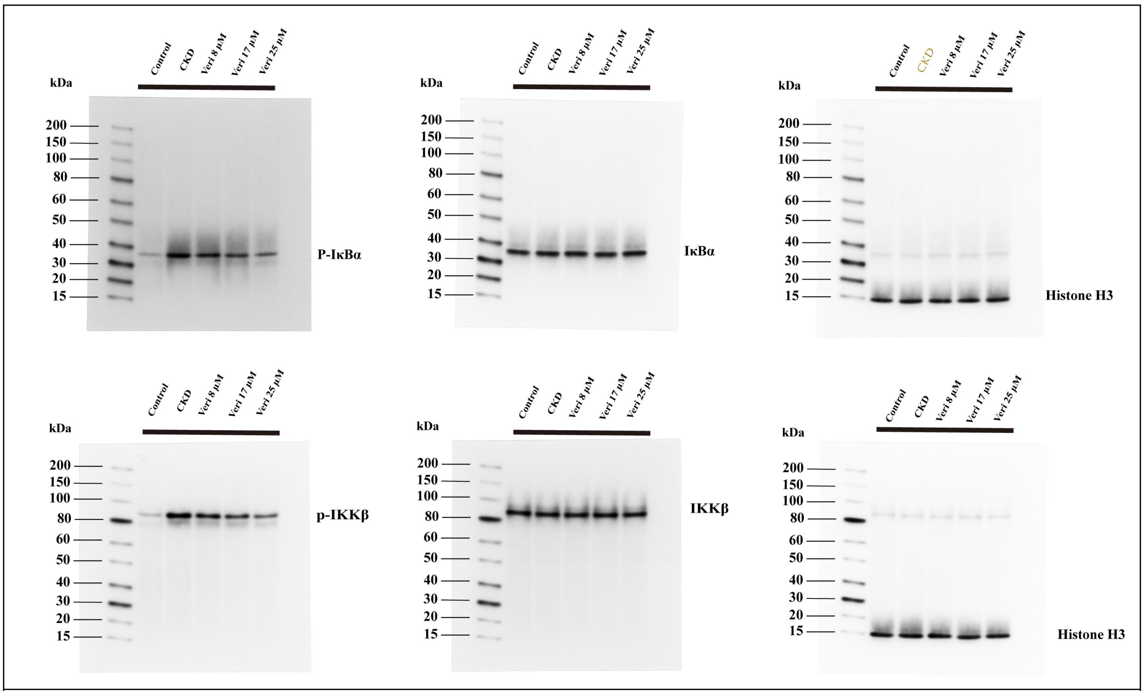

2. HK2 cell protein

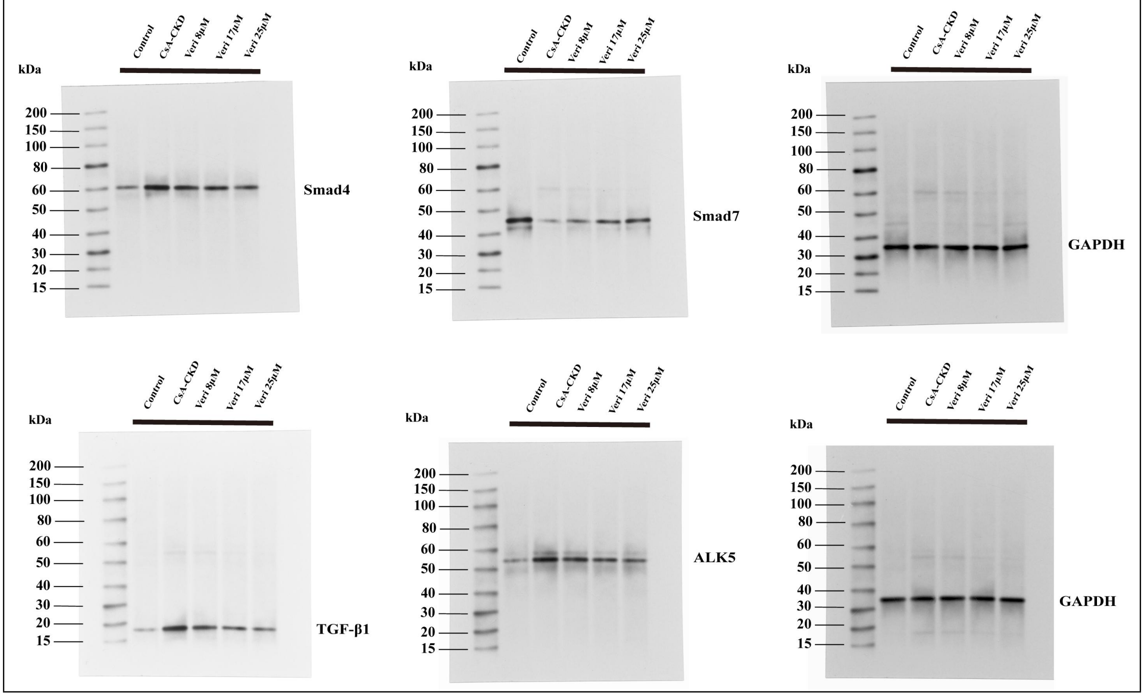

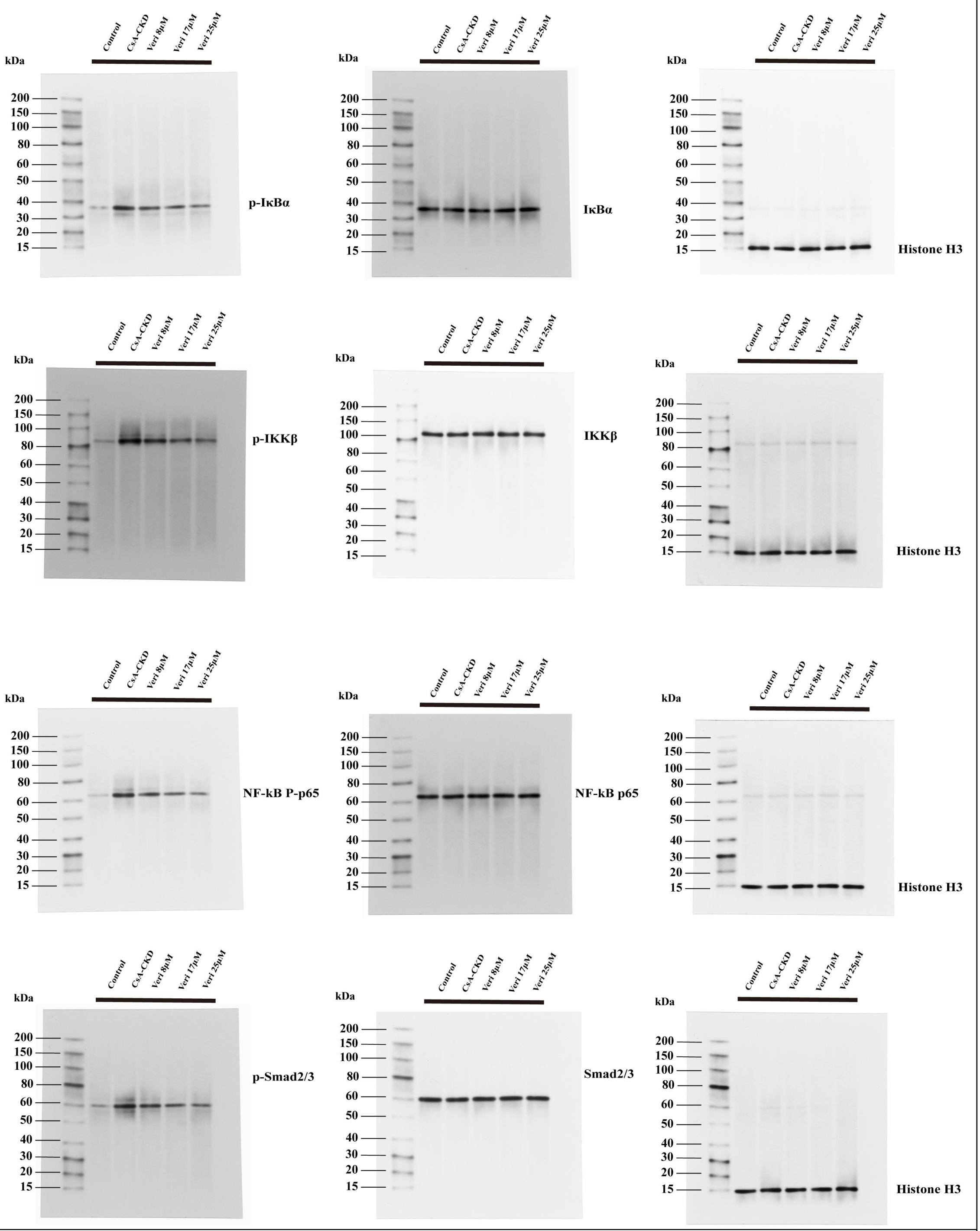

3. shRNA-p65 stably rotating HK2 protein

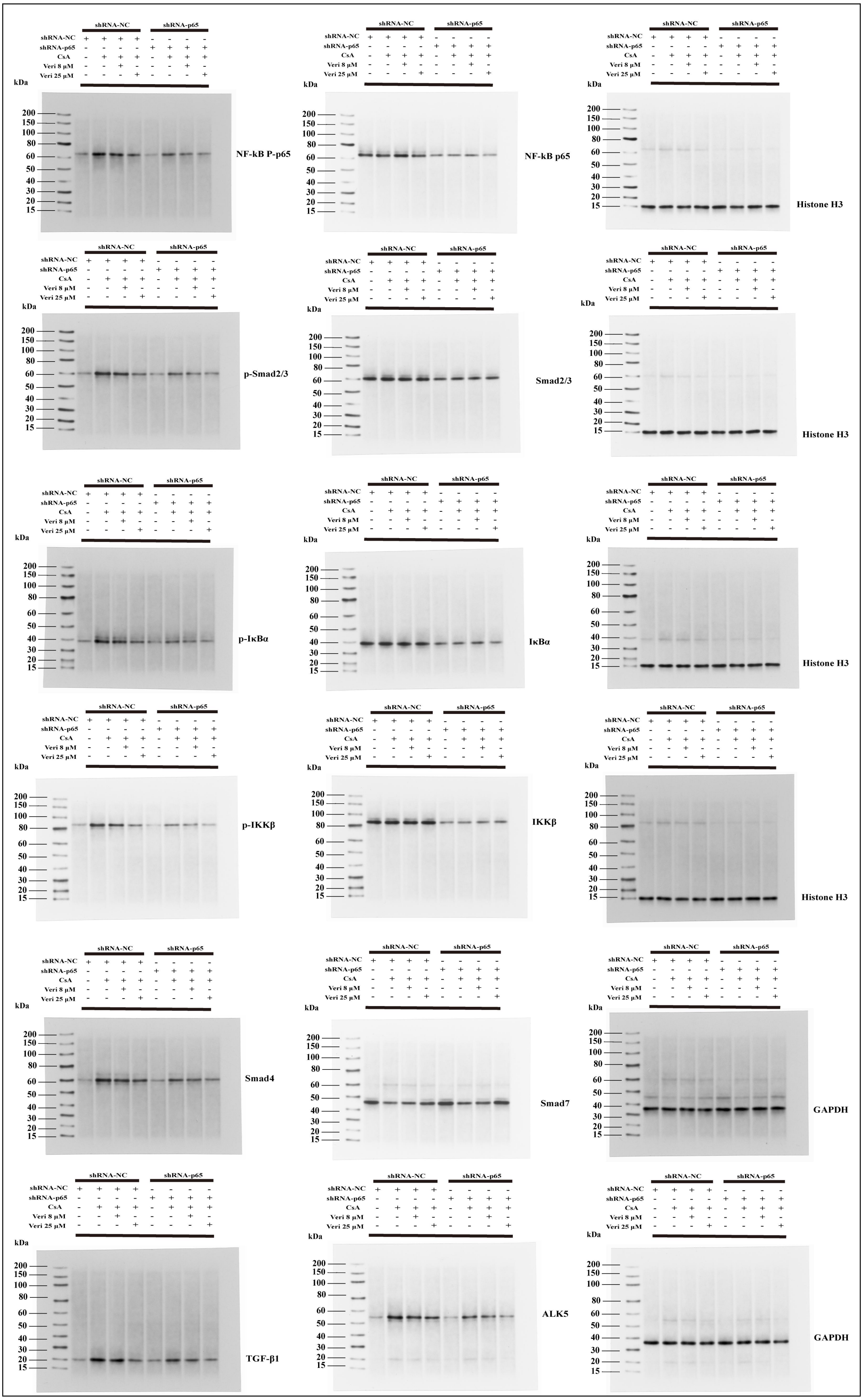
shRNA-NC
shRNA-p65
CsA
Veri 8 μM
Veri 25 μM
kDa
NF-kB P-p65
NF-kB p65
Histone H3
p-Smad2/3
Smad2/3
p-IκBα
IκBα
p-IKKβ
IKKβ
Smad4
Smad7
GAPDH
TGF-β1
ALK5

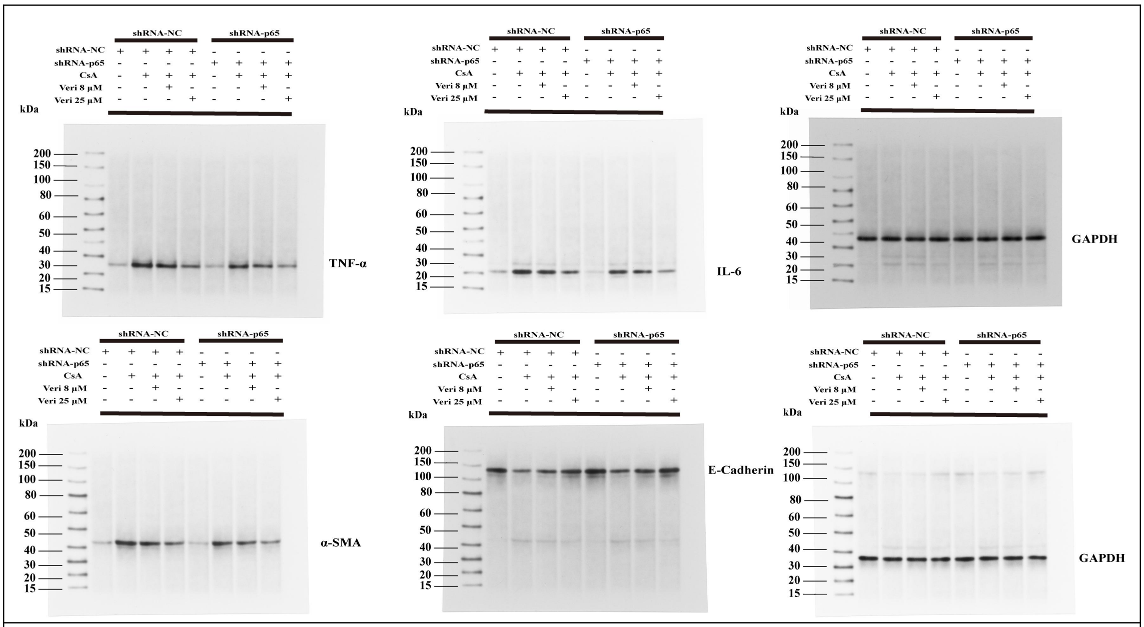

4. OE-p65 stably rotating HK2 protein

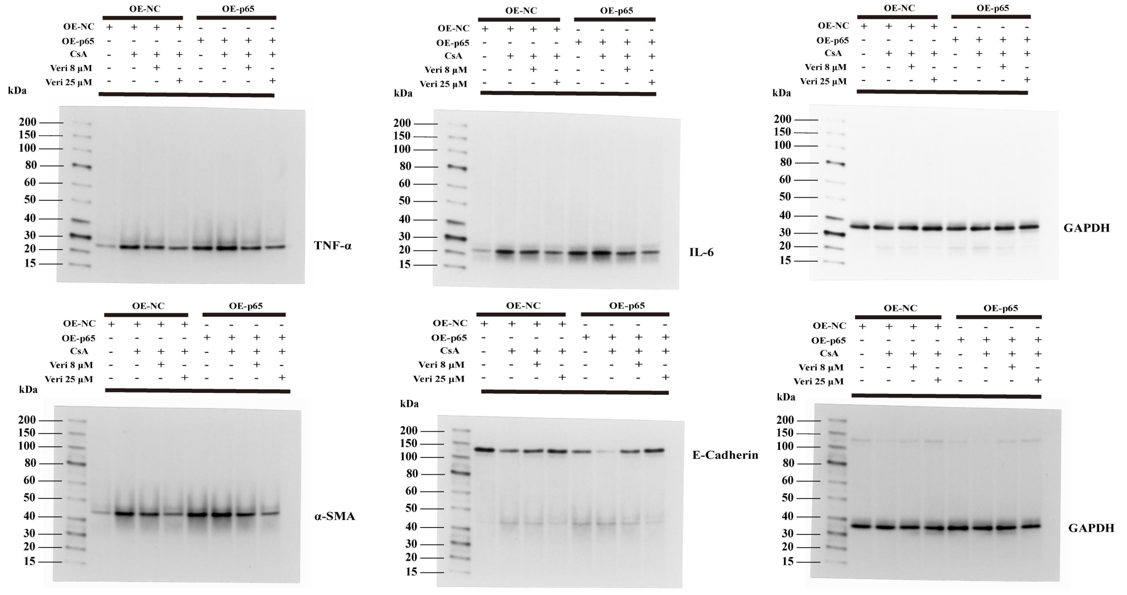

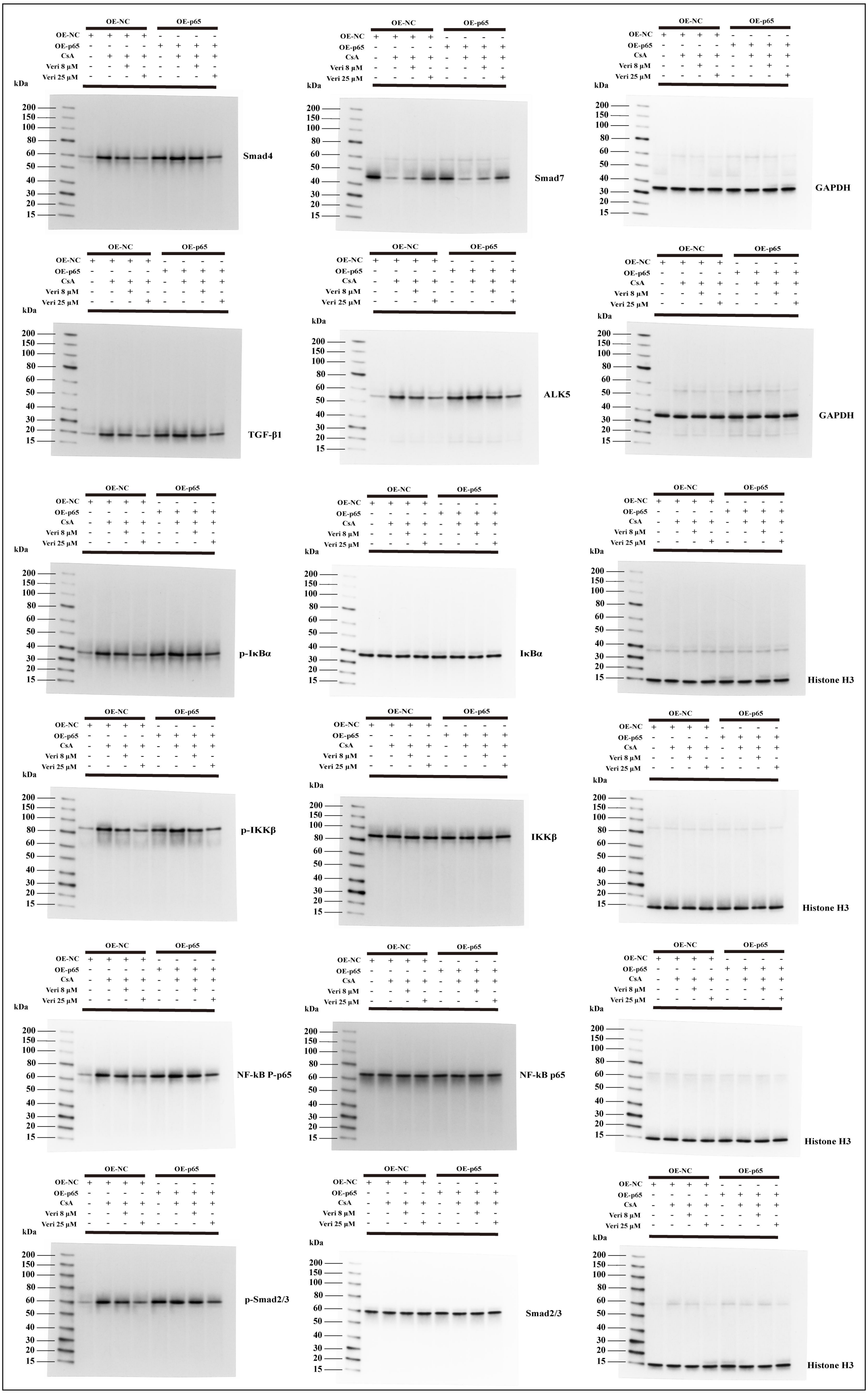
OE-NC
OE-p65
CsA
Veri 8 μM
Veri 25 μM
kDa
200
150
100
80
60
50
40
30
20
15
Smad4
Smad7
GAPDH
TGF-β1
ALK5
GAPDH
p-IκBα
IκBα
Histone H3
p-IKKβ
IKKβ
Histone H3
NF-kB P-p65
NF-kB p65
Histone H3
p-Smad2/3
Smad2/3
Histone H3
