# Supplementary material for: Vericiguat attenuates cyclosporine A-induced nephropathy by targeting the NF-κB/TGF-β1 axis: an integrated network pharmacology, Mendelian randomization, and experimental study
Source: Front Immunol. 2026 Jan 27;16:1756582. doi: 10.3389/fimmu.2025.1756582 (PMC12886507; doi:10.3389/fimmu.2025.1756582)
Supplement: Supplementary file 3 [file DataSheet3.pdf]

Supplementary material

Supplementary material associated with this article can be found, in the online version, at there:

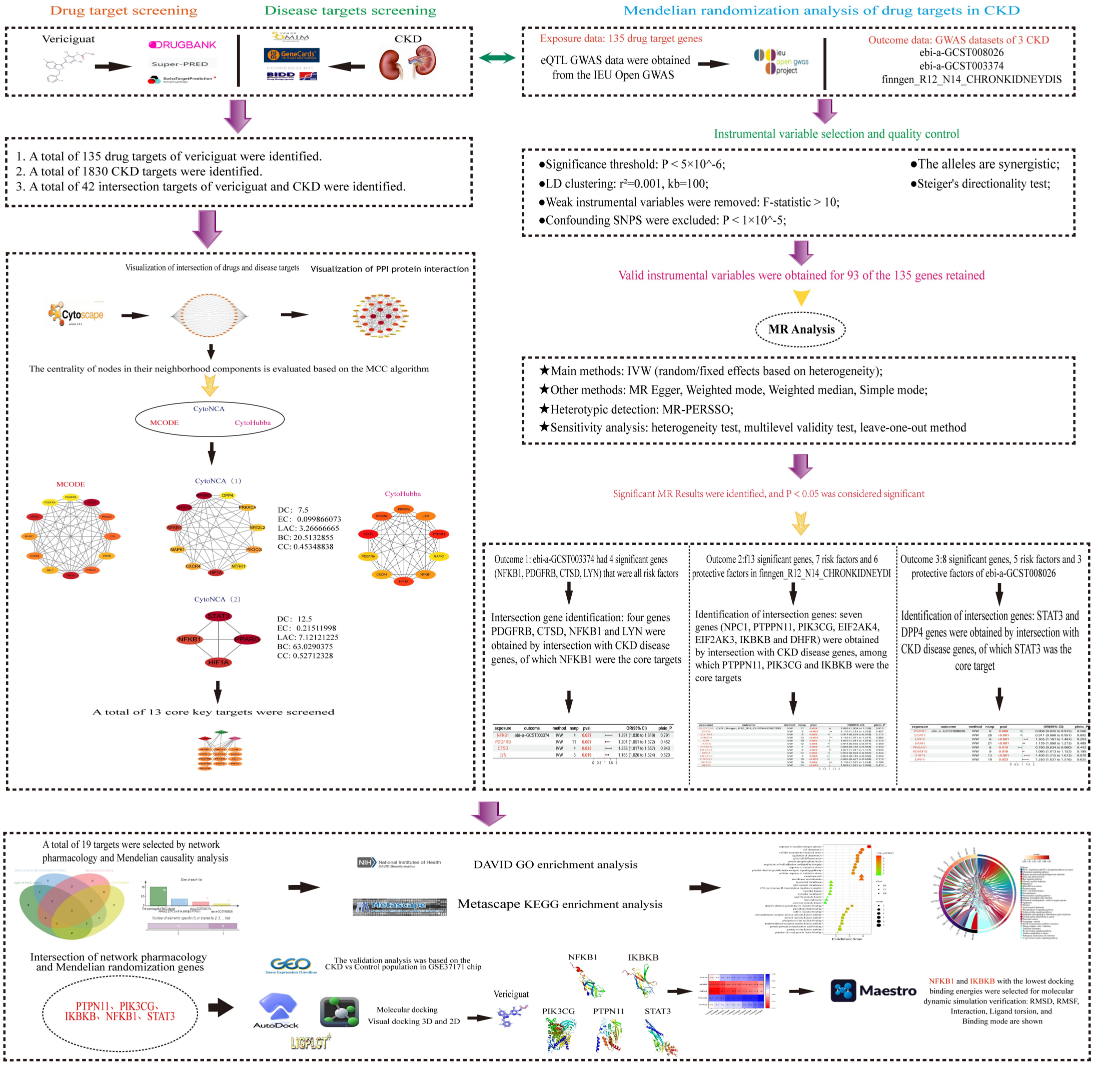

Figure 1. Schematic overview of the integrated research strategy combining network pharmacology, Mendelian randomization, and experimental validation to investigate the renoprotective mechanism of vericiguat in cyclosporine A-induced chronic kidney disease. The workflow illustrates the multi-step approach adopted in this study: (1) Identification of vericiguat and CKD overlapping targets via network pharmacology; (2) Causal inference of drug-target genes on CKD risk using Mendelian randomization analysis; (3) In vivo validation in a CsA-induced mouse model assessing renal function, histopathology, and molecular signaling; and (4) In vitro confirmation in human renal tubular epithelial (HK-2) cells under CsA stimulation, including genetic perturbation of p65. This integrative methodology bridges computational prediction with mechanistic experimentation to elucidate vericiguat's role in modulating the NF-κB/TGF-β1 axis.

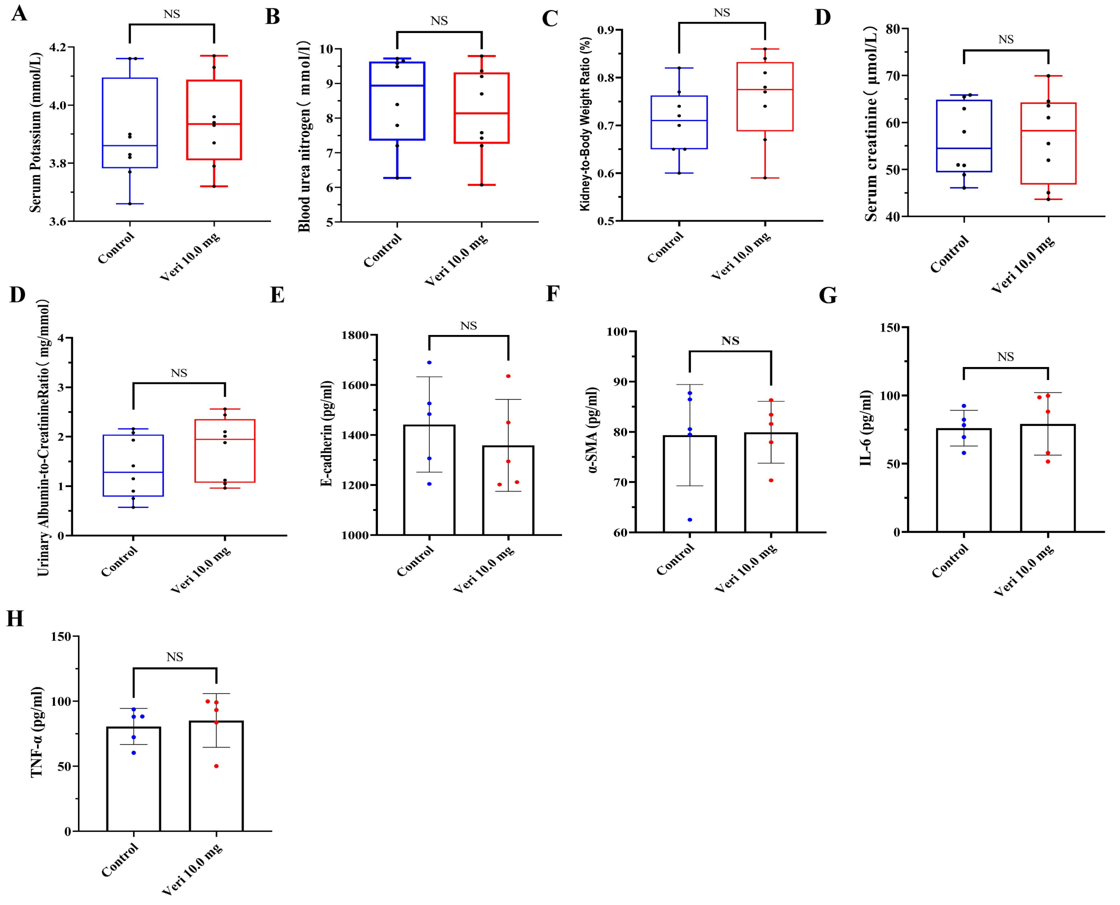

Figure 2. Vericiguat monotherapy does not induce renal toxicity or alter inflammatory/fibrotic markers in healthy mice. (A–D) Assessment of renal function and electrolyte balance in healthy mice administered Vericiguat alone (10 mg/kg/day, p.o.) for 12 weeks (n=8): (A) Serum creatinine, (B) blood urea nitrogen (BUN), (C) urinary protein-to-creatinine ratio (UPCR), and (D) serum potassium levels. (E–H) ELISA quantification of renal tissue levels of inflammatory and fibrotic markers:(E) IL-6, (F) TNF-α, (G) α-SMA, and (H) E-Cadherin. The detailed numerical data corresponding to panels (A–D) (renal function and serum potassium measurements at the 12-week endpoint) are provided in Supplementary Table 3.4. Data are presented as mean ± SD. Statistical analysis was performed using one-way ANOVA with Bonferroni post-hoc test for multiple comparisons. No significant differences were observed between the Vericiguat-alone group and the healthy control group (all P > 0.05), confirming that Vericiguat itself does not exert nephrotoxic, pro-inflammatory, or pro-fibrotic effects in the absence of CsA-induced injury. These results validate that the renoprotective effects observed in CsA-treated mice are attributable to specific modulation of disease pathways rather than confounding drug-related toxicity.

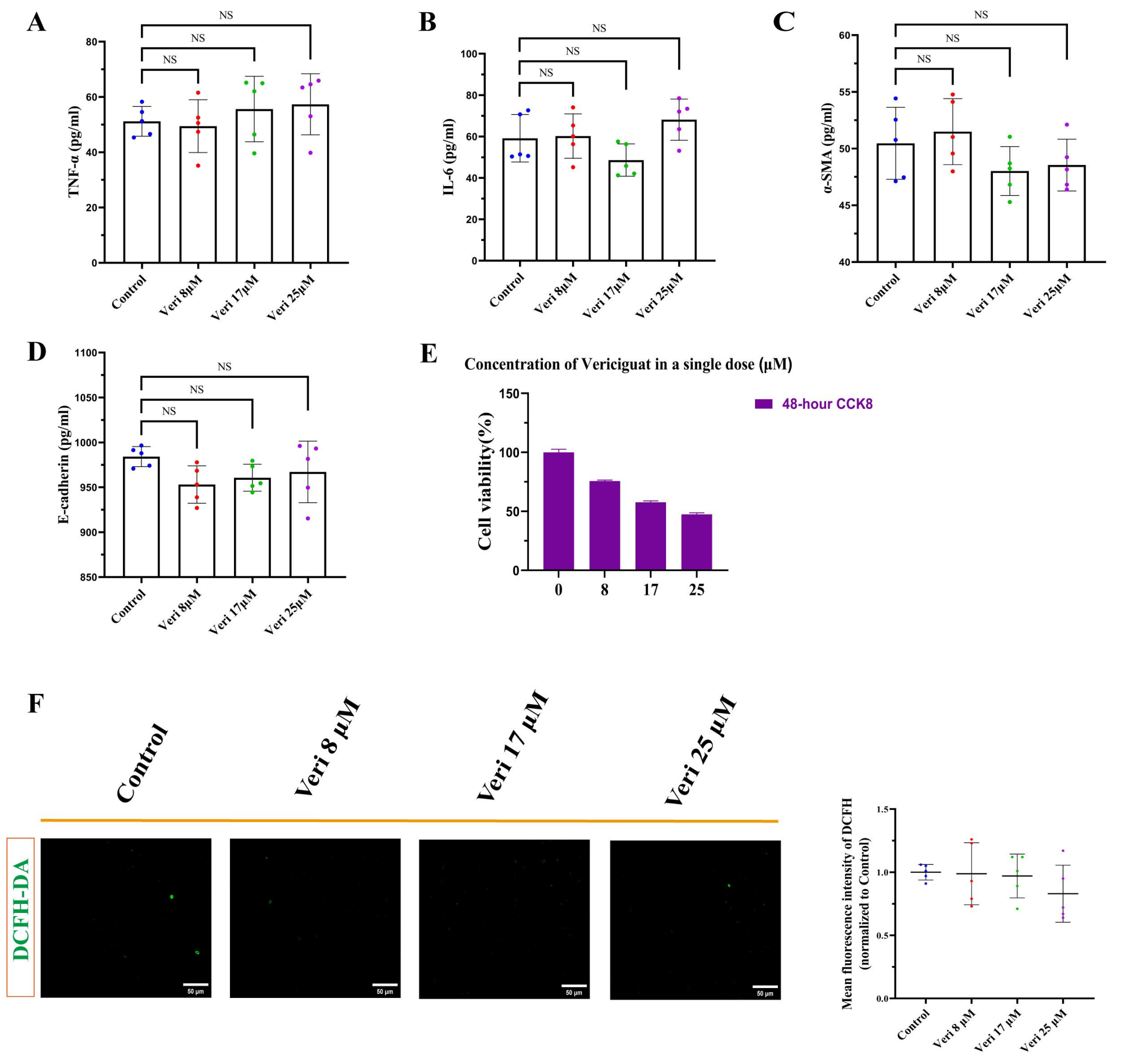

Figure 3. Vericiguat alone does not alter oxidative stress, inflammatory, or fibrotic markers in HK-2 cells (n=5, scale bar=50μM). (A–D) ELISA analysis of inflammatory and fibrotic markers in HK-2 cells following Vericiguat mon otherapy: (A) IL-6, (B) TNF-α, (C) α-SMA, and (D) E-Cadherin. (E) CCK8 was used to assess the toxicity of Vericig uat in the single-dose group at 48 hours (n=6). (F) Intracellular ROS levels measured by DCFH-DA staining in HK-2 cells treated with Vericiguat alone (8, 17, and 25 μM) for 48 h. Representative fluorescence images (scale bar: 50 μm) and quantitative analysis of mean fluorescence intensity are shown. Data are presented as mean ± SD (n = 5 and 6). Statistical analysis was performed using one-way ANOVA with Bonferroni post-hoc test. No significant differences we re observed between Vericiguat-treated groups and the control group (P > 0.05), indicating that the anti-inflammatory a nd anti-fibrotic effects of Vericiguat in CsA-stimulated cells are specific to pathway modulation rather than non-specific cytotoxicity.

Table 1-1 Outcome GWAS DATA

Consortium	ID	Trait	Population	Samples	SNPs
NA	ebi-a-GCST008026	Chronic	European	20920	17624171
NA	ebi-a-GCST003374	kidney	European	117165	2179497
finngen_R12	finngen_R12_N14_CHRONKIDNEYDIS	disease	European	493235	20463300

Notes：Consortium：database；ID：Number；Trait：name；Population：Race；Samples：the total number of samples；SNPs：total number of SNPS；Web source：Database website.

Table 1-2 MR Analysis of NFKB, STAT3, PTPN11, PIK3CG, and IKBKB with CKD

Genes	Outcome	Exposure	Method	Nsnp	B	SE	Pval	OR	OR_lCI95	OR_UCI95
NFKB1	ENSG00000168610 || eqtl-a-ENSG00000168610	CKD || ebi-a-GCST003374	MR Egger	4	0.566	1.032	0.638	1.762	0.233	13.321
			Weighted median	4	0.274	0.136	0.044	1.316	1.008	1.717
			Inverse variance weighted	4	0.256	0.115	0.027	1.291	1.030	1.619
			Simple mode	4	0.293	0.177	0.196	1.341	0.948	1.897
			Weighted mode	4	0.301	0.169	0.173	1.352	0.970	1.884
STAT3	ENSG00000168610 || eqtl-a-ENSG00000168610	CKD || ebi-a-GCST008026	MR Egger	13	0.329	0.207	0.140	1.389	0.926	2.085
			Weighted median	13	0.300	0.094	0.001	1.350	1.121	1.624
			Inverse variance weighted	13	0.336	0.072	3.246	1.400	1.215	1.613
			Simple mode	13	0.301	0.15	0.069	1.351	1.004	1.816
			Weighted mode	13	0.281	0.103	0.018	1.325	1.081	1.623
PTPN11	ENSG00000105851 || eqtl-a-ENSG00000105851	CKD || finngen_R12_N14_CHRONKIDNEYDIS	MR Egger	10	0.009	0.341	0.980	1.009	0.517	1.970
			Weighted median	10	-0.126	0.0439	0.004	0.881	0.809	0.960

Genes	Outcome	Exposure	Method	Nsnp	B	SE	Pval	OR	OR_lCI95	OR_UCI95
			Inverse variance weighted	10	-0.122	0.035	0.001	0.885	0.827	0.948
			Simple mode	10	-0.126	0.060	0.065	0.882	0.784	0.992
			Weighted mode	10	-0.128	0.054	0.042	0.880	0.792	0.978
			MR Egger	7	0.054	0.189	0.787	1.055	0.728	1.529
			Weighted median	7	-0.153	0.066	0.020	0.858	0.754	0.976
PIK3CG			Inverse variance weighted	7	-0.141	0.053	0.008	0.868	0.782	0.964
			Simple mode	7	-0.177	0.101	0.130	0.838	0.688	1.021
			Weighted mode	7	-0.150	0.076	0.096	0.861	0.741	0.999
			MR Egger	12	0.003	0.106	0.977	1.003	0.814	1.236
			Weighted median	12	-0.079	0.045	0.080	0.924	0.846	1.010
IKBKB			Inverse variance weighted	12	-0.090	0.034	0.008	0.914	0.855	0.977
			Simple mode	12	-0.073	0.070	0.321	0.929	0.809	1.067
			Weighted mode	12	-0.075	0.046	0.131	0.928	0.848	1.015

Notes：Exposure：The Exposure factor；Method：Analysis and test methods (focusing on IVW Method)；Nsnp：number of SNPS；B：beta effect size；SE：standard error of outcome；OR：hazard ratio, for dichotomous variables；OR_lci95：lower 95% confidence interval of hazard ratio；OR_uci95：Upper 95% confidence interval of hazard ratio.

Table 1-3 Heterogeneity test of exposure factors and outcomes between NFKB, STAT3, PTPN11, PIK3CG, and IKBKB and CKD

Genes	Exposure	Outcome	Method	Q	Q_df	Q_Pval
NFKB1	ENSG00000168610 || eqtl-a-ENSG00000168610	CKD || ebi-a-GCST003374	MR Egger	0.283	2	0.868
			Inverse variance weighted	0.374	3	0.946
STAT3	ENSG00000168610 || eqtl-a-ENSG00000168610	CKD || ebi-a-GCST008026	MR Egger	8.604	11	0.658
			Inverse variance weighted	8.606	12	0.736
PTPN11	ENSG00000105851 || eqtl-a-ENSG00000105851	CKD || finngen_R12_N14_CHRONKIDNEYDIS	MR Egger	3.049	8	0.931
			Inverse variance weighted	3.198	9	0.956
PIK3CG			MR Egger	0.547	5	0.990
			Inverse variance weighted	1.700	6	0.945
IKBKB			MR Egger	16.929	10	0.076
			Inverse variance weighted	18.496	11	0.0708

Notes：Exposure：The Exposure factor；Method：Analysis and test methods (focusing on IVW Method)；Q：Cochran's Q heterogeneity statistic；Q_df：degree of freedom.

Table 1-4 Exposure factor and outcome level pleiotropy tests for NFKB, STAT3, PTPN11, PIK3CG, and IKBKB and CKD

Genes	Exposure	Outcome	Egger_intercept	SE	Pval
NFKB1	ENSG00000105851 || eqtl-a-ENSG00000105851	CKD || finngen_R12_N14_CHRONKIDNEYDIS	-0.021	0.070	0.791
STAT3	ENSG00000168610 || eqtl-a-ENSG00000168610	CKD || ebi-a-GCST008026	0.001	0.032	0.970
PTPN11	ENSG00000105851 || eqtl-a-ENSG00000105851	CKD || finngen_R12_N14_CHRONKIDNEYDIS	-0.029	0.076	0.710
PIK3CG			-0.023	0.023	0.332
IKBKB			-0.019	0.020	0.359

Note: Exposure：The Exposure factor；Egger_intercept：intercept value；SE：standard error of intercept.

Table 2-1 Vericiguat was analyzed by MM-GBSA calculations with the active sites of both proteins

	△Coulomb	△Covalent	△vdW	△Lipo	△Solv GB	△Hbond	△Packing	△SelfCont	△MMGBSA dG Bind (kcal/mol)
Vericiguat & NFKB1	-13.03	2.64	-35.45	-12.69	19.98	-1.46	-4.56	0	-44.58
Vericiguat & IKKβ	-21.85	5.83	-38.44	-11.87	25.97	-2.6	-1.55	0	-44.51

△Coulomb: Coulomb energy; △Covalent: Covalent binding energy; △vdW: Van der Waals energy; △Lipo: Lipophilic energy; △Solv GB: Lipophilic energy; △Hbond: Hydrogen-bonding energy; △Packing: Pi-pi packing energy; △SelfCont: Self-contact correction; △MMGBSA dG Bind: Free energy of binding.

Table 3-1 Changes of 24-hour urinary protein in mice (mg/L/h, n=8, $\bar{X}$±SD)

Weeks	Control	CKD	Veri 2.5mg	Veri 5.0mg	Veri 10.0mg
0	39.27±9.37	32.64±7.33	27.08±6.75	34.99±9.57	32.77±9.97
2	41.36±3.47$^{\#\#}$	160.72±6.28	160.68±6.69	150.93±6.17	137.21±8.01
4	38.13±7.52$^{\#\#\#}$	230.86±13.59	228.82±13.94	228.82±13.94	237.55±8.06
6	31.76±10.12$^{\#\#\#}$	323.56±9.75$^{NS/*/**}$	299.40±8.54$^{NS/*}$	267.49±9.58^{NS}	240.60±240.6
8	35.22±8.53$^{\#\#\#}$	534.67±16.86$^{*/**/***}$	482.72±19.49$^{*/**}$	426.52±15.13*	387.58±12.62***
10	37.85±7.89$^{\#\#\#}$	832.00±16.83$^{*/**/***}$	765.27±33.11$^{*/**}$	721.97±16.47*	687.82±20.50***
12	30.90±9.98$^{\#\#\#}$	1323.53±59.99$^{**/***/***}$	1165.31±23.54$^{*/**}$	1107.58±26.89**	944.94±22.11***

Notes：Contro vs CKD: $^{\#\#}$, P＜0.01、$^{\#\#\#}$, P＜0.001; CKD vs Different dose drug groups: *, P＜0.05、**, P＜0.01、***, P＜0.001. Vericiguat 2.5mg vs Vericiguat 5mg: *, P＜0.05、**, P＜0.01、***, P＜0.001. Vericiguat 2.5mg vs Vericiguat 10mg: *, P＜0.05、**, P＜0.01、***, P＜0.001. Vericiguat 5mg vs Vericiguat 10mg: *, P＜0.05、**, P＜0.01、***, P＜0.001.（NS，P＞0.05）

Table 3-2 Changes in body weight of mice (g, n=8, $\bar{X}$±SD)

Weeks	Control	CKD	Veri 2.5mg	Veri 5.0mg	Veri 10.0mg
0	15.71±0.80	15.74±1.24	16.09±0.99	15.90±1.39	15.49±0.94
2	22.36±1.44	22.41±1.17	21.68±1.36	22.70±1.59	21.52±1.48
4	22.79±0.98	21.60±1.30	24.76±1.57	24.56±1.78	25.55±1.29
6	25.16±0.99$^{\#}$	22.64±1.02$^{NS/NS/*}$	22.58±1.92$^{NS/*}$	24.44±1.46^{NS}	25.69±1.28
8	29.89±1.46$^{\#\#}$	23.00±1.76$^{NS/*/*}$	23.64±1.33$^{*/*}$	25.44±0.99^{NS}	26.02±1.56
10	32.49±1.40$^{\#\#\#}$	22.84±1.37$^{NS/*/**}$	23.32±1.40$^{*/*}$	25.45±1.04*	27.32±1.38
12	33.81±1.48$^{\#\#\#}$	21.53±0.94$^{NS/*/**}$	22.28±1.32$^{*/**}$	25.89±0.66*	28.26±1.67

Notes：Contro vs CKD: $^{\#\#}$, P＜0.01、$^{\#\#\#}$, P＜0.001; CKD vs Different dose drug groups: *, P＜0.05、**, P＜0.01. Vericiguat 2.5mg vs Vericiguat 5mg: *, P＜0.05、**, P＜0.01. Vericiguat 2.5mg vs Vericiguat 10mg: *, P＜0.05、**, P＜0.01. Vericiguat 5mg vs Vericiguat 10mg: *, P＜0.05、**, P＜0.01.（NS，P＞0.05）

Table 3-3 Changes of blood biochemistry and renal function in mice (n=8, $\bar{X}$±SD)

	Control	CKD	Veri 2.5mg	Veri 5.0mg	Veri 10.0mg
Serum creatinine (μmol/L)	56.15±7.93$^{\#\#\#}$	394.30±34.19$^{*/**/***}$	388.70±10.33$^{*/***}$	313.70±8.65**	278.10±14.56
Blood urea nitrogen（mmol/l）	8.51±1.32$^{\#\#\#}$	100.20±4.06$^{***/***/***}$	78.14±5.70$^{NS/**}$	72.43±5.47*	51.49±5.75
Serum Potassium (mmol/L)	3.89±0.18$^{\#\#}$	5.79±0.48$^{NS/*/**}$	5.67±0.24$^{NS/**}$	4.88±0.22*	4.11±0.23
Urinary Albumin-to-CreatinineRatio（mg/mmol）	1.37±0.63$^{\#\#\#}$	89.79±6.26$^{*/**/***}$	66.04±8.28$^{NS/***}$	50.65±9.27*	29.39±7.02
Kidney-to-Body Weight Ratio (%)	0.71±0.07$^{\#\#\#}$	3.21±0.41$^{NS/*/***}$	2.91±0.26$^{*/**}$	1.91±0.29*	1.39±0.20

Notes：renal function and serum potassium measurements at the 12-week endpoint. Contro vs CKD: $^{\#\#}$, P＜0.01、$^{\#\#\#}$, P＜0.001; CKD vs Different dose drug groups: *, P＜0.05、**, P＜0.01、***, P＜0.001. Vericiguat 2.5mg vs Vericiguat 5mg: *, P＜0.05、**, P＜0.01、***, P＜0.001. Vericiguat 2.5mg vs Vericiguat 10mg: *, P＜0.05、**, P＜0.01、***, P＜0.001. Vericiguat 5mg vs Vericiguat 10mg: *, P＜0.05、**, P＜0.01、***, P＜0.001.（NS，P＞0.05）

Table 3-4 Effect of a single dose of the drug on blood biochemistry and renal function in mice (n=8, $\overline{X}$±SD)

	Control	Veri 10.0mg
Serum creatinine (μmol/L)	56.15±7.93^{NS}	56.92±9.45
Blood urea nitrogen（mmol/l）	8.51±1.32^{NS}	8.17±1.29
Serum Potassium (mmol/L)	3.89±0.18^{NS}	3.94±0.15
Urinary Albumin-to-CreatinineRatio（mg/mmol）	1.37±0.63^{NS}	1.77±0.64
Kidney-to-Body Weight Ratio (%)	0.71±0.07^{NS}	0.76±0.09

Notes：renal function and serum potassium measurements at the 12-week endpoint. Contro vs Vericiguat 10mg: ^{NS}, P>0.05.

Table 4-1 Sequences of the primers for real-time PCR

Mouse Gene	Forward	Reverse
p65	5‘- CCAGACACAGATGATCGCCAC -3’	5‘-TGGGGACAGAAGTTGAGTTTCG-3’
IKBα	5‘- AAAATCTCCAGATGCTACCCGA -3’	5‘-GGCCTCCAAACACACAGTCAT-3’
IKKβ	5‘-GAGCCTTATGAACGAGGACGAG-3’	5‘- CACTGGAAGGCTGGGACATTAG-3’
Smad2	5‘-ATGACTACACCCACTCCATTCCA-3’	5‘- AAGCCGTCTACAGTGAGCGAG-3’
Smad3	5‘-GGAATGCAGCCGTGGAACTT-3’	5‘- TTGCAGCCTGGTGGGATCTT-3’
Smad4	5‘-TATGCCCGTCTGTGGAGGTG-3’	5‘- CTTCAGTGGGTAAGGACGGCT-3’
Smad7	5‘-CAGGCATTCCTCGGAAGTCA-3’	5‘- TTGGGTATCTGGAGTAAGGAGGA-3’
TGF-β1	5‘-TAATGGTGGACCGCAACAAC-3’	5‘- CCACATGTTGCTCCACACTTGAT-3’
ALK5	5‘-AATCCATGAAGACTATCAGTTGCCT-3’	5‘- CCTTCCTGTTGGCTGAGTTGT-3’
GAPDH	5‘-AACTTTGGCATTGTGGAAGG-3’	5‘-ACACATTGGGGGTAGGAACA-3’
Human Gene		
p65	5‘- ACCGGATTGAGGAGAAACGTA -3’	5‘- TCTGCCCAGAAGGAAACACC -3’
GAPDH	5‘- GAAGGTGAAGGTCGGAGTC -3’	5‘- GAAGATGGTGATGGGATTTC -3’

Notes：Primer sequences were designed using NCBI Primer-BLAST and validated by melt curve analysis. GAPDH served as the endogenous control for normalization. Gene-specific primers targeted NF-kB pathway and TGF-β1/Smad pathway components. All primers showed 90-110% amplification efficiency in validation experiments.
